# Extracellular DNA Traps: Origin, Function and Implications for Anti-Cancer Therapies

**DOI:** 10.3389/fonc.2022.869706

**Published:** 2022-04-27

**Authors:** Medina Mamtimin, Akif Pinarci, Chao Han, Attila Braun, Hans-Joachim Anders, Thomas Gudermann, Elmina Mammadova-Bach

**Affiliations:** ^1^ Walther-Straub-Institute for Pharmacology and Toxicology, Ludwig-Maximilians-University, Munich, Germany; ^2^ Division of Nephrology, Department of Medicine IV, Ludwig-Maximilians-University Hospital, Munich, Germany; ^3^ German Center for Lung Research, Munich, Germany

**Keywords:** extracellular DNA traps, cancer, inflammation, immunity, thrombosis, anti-cancer therapies

## Abstract

Extracellular DNA may serve as marker in liquid biopsies to determine individual diagnosis and prognosis in cancer patients. Cell death or active release from various cell types, including immune cells can result in the release of DNA into the extracellular milieu. Neutrophils are important components of the innate immune system, controlling pathogens through phagocytosis and/or the release of neutrophil extracellular traps (NETs). NETs also promote tumor progression and metastasis, by modulating angiogenesis, anti-tumor immunity, blood clotting and inflammation and providing a supportive niche for metastasizing cancer cells. Besides neutrophils, other immune cells such as eosinophils, dendritic cells, monocytes/macrophages, mast cells, basophils and lymphocytes can also form extracellular traps (ETs) during cancer progression, indicating possible multiple origins of extracellular DNA in cancer. In this review, we summarize the pathomechanisms of ET formation generated by different cell types, and analyze these processes in the context of cancer. We also critically discuss potential ET-inhibiting agents, which may open new therapeutic strategies for cancer prevention and treatment.

## Introduction

Extracellular deoxyribonucleic acid (DNA) can be detected in extracellular environments, including serum, urine, spinal fluid, amniotic fluid, cerebrospinal fluid, lymph, bile and milk. In 1948, Mandel and Métais described for the first time the presence of DNA in the plasma of cancer patients (1). Extracellular DNA comprises nuclear or mitochondrial DNA associated with proteins or extracellular vesicles (2). Pioneer studies by Leon et al., described that patients with cancer have elevated levels of extracellular DNA, and its reduction following radiotherapy could significantly improve the clinical conditions (3). Follow-up studies provided evidence that extracellular DNA levels are elevated in many cancer patients, especially with invasive metastatic cancer (3–5). Liquid biopsy-based diagnostic and prognostic approaches including the analysis of circulating tumor cells, ribonucleic acids (RNAs), extracellular vesicles and extracellular DNA became powerful tools for the therapeutic management of cancer patients (6–8). However, the variability of tumor-specific markers in extracellular DNA sequences and alterations in levels of extracellular DNA in cancer patients raised several questions about their origin. Two different hypotheses explained the origin of extracellular DNA; extracellular DNA is the product of cellular breakdown or generated by an active release mechanism (9). Cellular breakdown induces DNA release from dividing cancer cells, or products of cell lysis, apoptosis or necrosis following cancer treatments (10, 11). The theory of active release mechanism was supported by studies describing neutrophil-extracellular traps (NETs) as a process of immune defense inducing extracellular DNA release together with histones, radical oxygen species (ROS), peroxidases to trap and eradicate pathogens (12). Clinical and experimental studies highlighted the pivotal role of neutrophils in inflammation, thrombosis and cancer (13). NETs were found in liquid and tissue biopsies of cancer patients (14–18). Over the last years, many studies linked the process of NETosis to oncogenic transformation, angiogenesis, cancer development and metastasis (19, 20). In different pathological contexts (thromboinflammation, atherosclerosis, systemic lupus erythematosus, infection, sepsis), it became also evident that other blood, immune and specialized cells could also generate extracellular traps (ETs) (21, 22). In this review, we provide a detailed analysis of extracellular DNA function in cancer and also discuss the different sources and origins of ETs and provide the hypotheses on their possible impact on tumor cells and tumor microenvironment.

## Neutrophil Extracellular Traps

Under physiological conditions, polynuclear neutrophils represent the main subpopulation of white blood cells, approximately 50-70% of circulating leukocytes (23). Neutrophils are produced in the bone marrow and differentiate from hematopoietic stem cell precursors (24). Their number oscillates in the peripheral blood and is regulated by the circadian rhythm (25). Neutrophils play an important effector role in innate immunity, constantly patrolling the organism against microbial infections and invading pathogens (26). Neutrophils respond to pathogens in several ways: phagocytosis (27) and release of granular contents (28) and NETs (12). Neutrophils express many inflammatory mediators, such as complement components (29), receptors for Fc fragments of immunoglobulins, integrins and cytokines, thereby regulating host defense, inflammation and cell-cell interactions (30). Neutrophils have polylobulated nuclei composed of 3-5 lobules (31), and secretory granules in the cytoplasm (32). Neutrophil granules are classified into 4 categories, based on their granule content (33); primary or azurophilic granules, containing myeloperoxidase (MPO), anti-microbial peptides (defensins), β-glucuronidase (34), lysozyme and serine proteases (neutrophil elastase (NE), cathepsins G, proteinases 3 (PR3), inducible nitric oxide synthase (iNOS) (35), secondary or specific granules containing lactoferrin, matrix metalloproteinase (MMP) 8 (36), tertiary or gelatinase granules containing MMP9 (37), LL-37 (38), NADPH oxidase and mobilizable secretory vesicles containing various surface membrane receptors (39). The granular content of neutrophils plays an important role in NETosis (12). Consistently, immature neutrophils with reduced granular content from acute myeloid leukemia patients had a lower potential to induce NETosis after phorbol 12-myristat 13-acetate (PMA) stimuli (40).

In 2004, research groups of Zychlinsky and Brinkmann demonstrated that neutrophils in response to pathogens generate extracellular fibers composed of decondensed DNA, decorated with anti-microbial peptides and other proteins from different cell compartments, and later this process was defined as NETosis (12, 41). NETosis was induced by stimulation of neutrophils with pathogens (fungi, bacteria, protozoa, parasites), bacterial lipopolysaccharide (LPS), interleukin 8 (IL8) or chemical stimulation with protein kinase C (PKC) activator PMA, indicating that NETs are involved in inflammatory and infectious processes (12, 42, 43). Endothelial cell-derived cytokines, such as IL8 also act on neutrophils, thereby inducing NET formation (44). NETs have been found in the blood of septic patients (45–47). Platelet-derived Toll-like receptor 4 (TLR4) appeared to play an essential role in the NET formation through binding to the bacterial LPS (48).

Depending on the stimulation of ET release, neutrophils become apoptotic (lethal NETosis) or can still survive (vital NETosis). The process of lethal NETosis is often induced by pharmacological, autoimmune or metabolic compounds or bacterial peptides (49–51). In contrast, vital NETosis is preferentially induced by molecules associated with pathogen-associated molecular pattern molecules (PAMPs), which are recognized by TLRs of the innate immune system and also by bacterial peptides (48, 52–54).

NET webs and granular proteins can eradicate a wide range of pathogens by ensuring their capture, providing a scaffold for protein binding, degrading pathogen toxins and by providing a high local concentration of anti-microbial molecules (43).

## Molecular Mechanisms of Neutrophil Extracellular Trap Formation

At the molecular level, NETosis is regulated by MEK (MAPK/ERK kinase) or ERK (Extracellular-signal Regulated Kinase) (55), IRAK (IL1 Receptor-Associated Kinase) (56), PKC (57), Phosphoinositide 3-kinase (PI3K) (58) and AKT (59) pathways, inducing ROS production in response to the inflammatory mediators (60, 61), PMA (62), microorganisms (63, 64) and immune complexes (62, 65, 66). Terminally differentiated neutrophils undergo NETosis followed by the reactivation of cyclin-dependent kinase 6 (CDK6). Consequently, inhibition or knock-out of CDK6 function leads to reduced ability of neutrophils to induce NETosis (67). Some of these pathways are highly dependent on the NADPH oxidase 2 (Nox2), and ROS production (59). Nox2 is a multidomain complex enzyme, and its activity is regulated by protein PKC-dependent activation of p47phox, p67phox and p21rac subunits which form complex with b558 (68, 69). ROS production in neutrophils generates an optimal pH (7.5-8.5) for NE and MPO which are essential for NETosis (70). Consistently, neutrophils isolated from MPO-deficient patients display impaired bacterial killing and NETosis upon stimulation with PMA (71). The increase in pH level stimulates ROS production and induces histone H4 cleavage (70). In PMA-stimulated neutrophils hypochlorous acid (HOCl) disassembles the azurosome, leading to the release of NE into the cytoplasm (72). Later, NE degrades F-actin and translocates into the nucleus and breaks histone H1 (73). NE and MPO facilitate chromatin decondensation and the loss of lobular structure of the nucleus. Following this process, the nuclear envelope disassembles into vesicles thereby mixing both the cytoplasm and nucleoplasm. In the cytoplasm, decondensed chromatin binds granular and cytoplasmic anti-microbial proteins such as NE and MPO, before rupturing the cytoplasmic membrane for NET formation (49, 74). Interestingly, NET formation upon stimulation with PMA or crystals (nano- and microparticles) can also involve receptor-interacting serine/threonine-protein kinases (RIPK1 and RIPK3) and mixed lineage kinase domain-like pseudokinase (MLKL)-dependent pathway of necroptosis (75–77).

NETs can also form independently of Nox-signaling. This occurs through an influx of extracellular calcium (Ca^2+^) through Ca^2+^ ionophores, such as ionomycin and A32178 which are secreted by the gram-positive bacteria (78–80). Although Nox-induced ROS production is not involved in this type of NETosis, Ca^2+^ ionophores can induce ROS production using an alternative pathway in the mitochondria (81). Nox-independent NETosis needs potassium (K^+^) influx through the activation of small-conductance Ca^2+^-activated K^+^ SK3 channels. In this pathway, ERK and Akt signaling are activated at low or moderate levels, compared to Nox-dependent NETosis, and similar levels of p38 activation were found in both pathways (82).

## Tumor-Associated NETs

**Figure 1:**
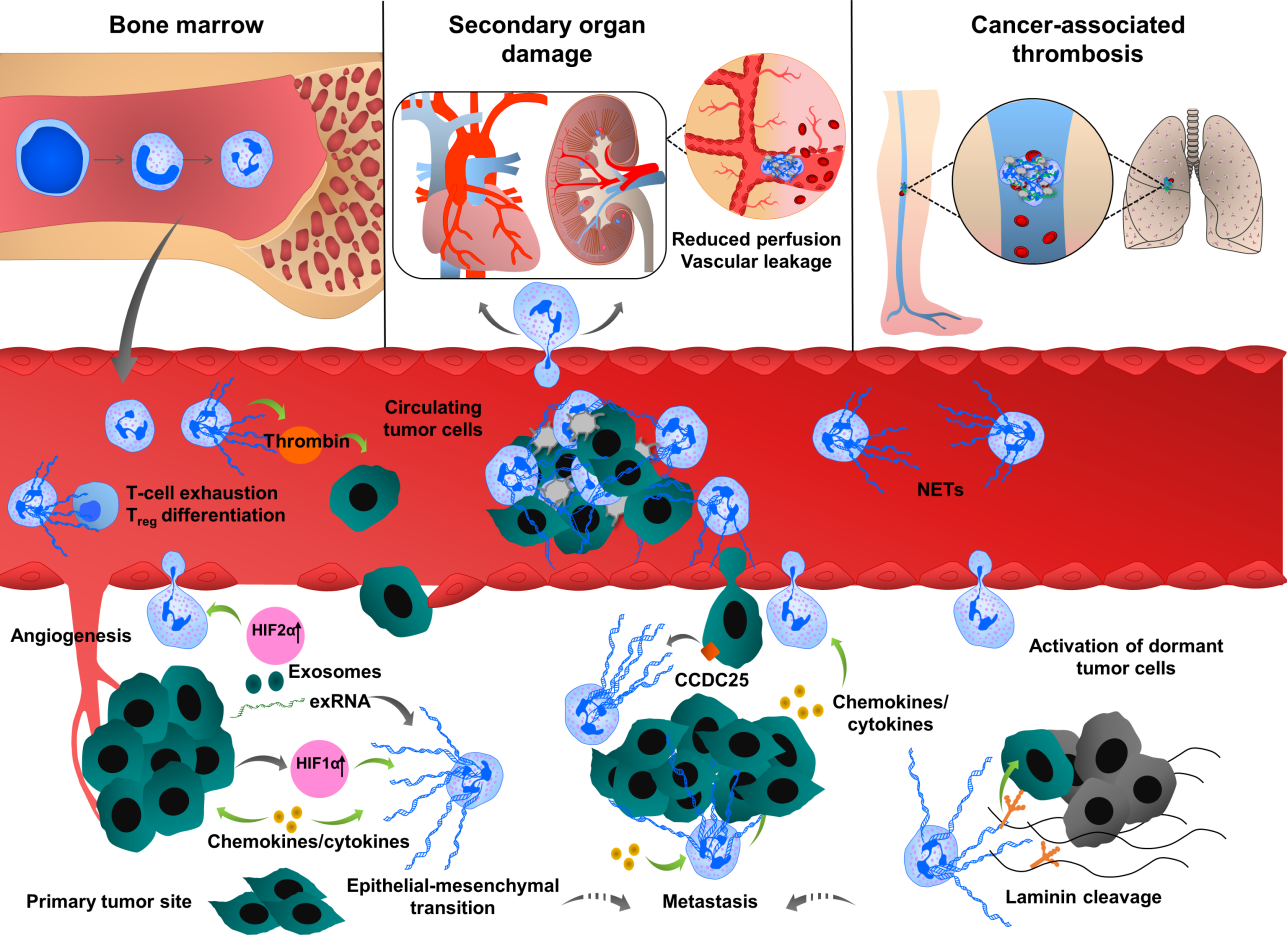
Multiple roles of neutrophil extracellular traps (NETs) in tumor progression and metastasis. Neutrophils are mobilized from bone marrow, enter into the circulation and migrate towards proangiogenic and proinflammatory gradients. Neutrophils are recruited to the primary tumor site through various cytokines and chemokines such as CXCL1, IL6 or CCL3, ultimately leading to neutrophil activation and NET release. Cancer cell-derived exRNA can also induce NETs which in turn amplify the release of exRNA. In growing tumors, NETs enhance cancer progression by enhancing thrombin activity, increasing the expression of stem cell markers and inflammatory chemokines and cytokines and promoting epithelial-mesenchymal transition. NET formation is also enhanced by the uptake of exosomes transporting oncogenic mutations to the tumor sites. NETs regulate cancer cell migration and tumor growth by directly interacting with T cells, inducing the exhaustion of cytotoxic T cells and differentiation of naïve T cells into regulatory T cells, thereby promoting an immunosuppressive environment. During their transit in the circulatory system, cancer cells are captured by the chromatin web network of NETs and this physical and functional interaction provides shielding thereby protecting cancer cells from cytotoxic effects of immune cells. NETs also provide an “anchor” to the cancer cells, facilitating their adhesion and extravasation into the secondary tumor sites to form distant metastasis. CCDC25 is expressed by cancer cells and can serve as a NET-DNA receptor that senses NETs and recruits invasive cancer cells to the metastatic sites. During inflammation, NETs can activate dormant tumor cells and stimulate them to migrate and form metastasis by cleaving basement membrane components (laminins). NETs also induce thromboinflammation leading to ischemia and injury in organs, such as the heart and kidney. Cancer cell-derived G-CSF predisposes circulating neutrophils to form NETs through the recruitment of blood platelets. Interactions between platelets and neutrophils play an important role in cancer progression and metastasis by inducing platelet activation and NETosis and consequently enhancing tumor-associated coagulation and thrombosis.

NET formation was detected in different phases of tumor progression and metastasis (14, 17, 83–85), (**Figure 1**). At the early phase of cancer, NETosis supports the epithelial-mesenchymal transition. Treatment of gastric and breast cancer cells with NETs induces an aggressive mesenchymal phenotype, thereby increasing cancer progression (86, 87). NETs induce gene expression of cancer stem cell marker CD24, and proinflammatory factors, such as IL1β, IL6, IL8, CXC motif chemokine receptor 1 (CXCR1), MMP2, MMP9 in cocultured luminal breast cancer cells (86). NETs also promote epithelial-mesenchymal transition in pancreatic ductal adenocarcinoma (PDAC). In clinical settings, increased levels of NETs were correlated with epithelial-mesenchymal transition markers in patients diagnosed with PDAC (88). At a later phase, the primary tumor starts to express many factors to stimulate NETosis. Systemic inflammation and hypoxia in the tumor and tumor microenvironment are important factors to induce neutrophil infiltration and NETosis (89–91). Hypoxia increases the levels of β2 integrin on the neutrophil surface in a hypoxia-inducible factor-1α (HIF1α)-dependent manner, and consequently, pharmacological blockade or knock-down of HIF1α in neutrophils inhibits NET formation (92, 93). HIF-2α also contributes to the recruitment of neutrophils to colon tumors, enhancing colon cancer progression through enhancing CXCL1 chemokine expression (94). Several other chemokines and cytokines are involved in the regulation of cancer-associated NETosis, regulating diverse signaling pathways. In human and mouse breast cancer, recent studies reported the role of tumor cell-secreted protease cathepsin C-mediated signaling in neutrophil recruitment and NET formation. In this pathological context, cathepsin C activates neutrophil membrane-bound proteinase 3 (PR3), thereby facilitating IL1β and Nuclear Factor kappa-light-chain-enhancer of activated B cells (NF-kb) activation, which in turn enhances neutrophil recruitment through the upregulation of IL6 and C-C Motif Chemokine Ligand 3 (CCL3) (95). Cancer cells also release exosomes to stimulate neutrophil chemotaxis and NET formation. Colon cancer cells transfer mutant KRAS to the neutrophils through exosomes, thereby promoting NETosis through the upregulation of IL8 which subsequently induces tumor growth, invasion and migration (96). It has been shown that neutrophils isolated from peripheral blood of mice bearing chronic myeloid lymphoma, lung and breast carcinoma tumors are more prone to generate NETs compared to the neutrophils isolated from healthy mice (97). In cancer models, neutrophil depletion and or DNAse I injection restored vascular perfusion and prevented vascular leakage (98). NETs were also shown to enhance endogenous effector functions of thrombin in plasma, thereby inducing cancer cell migration, invasion and angiogenesis (16, 99–101).

NET formation was also detected in the metastatic niche and plays an important role in different steps of metastasis, including tumor cell adhesion (19, 102, 103), dissemination (14) and extravasation at the distant organs. Several proteases and adhesion molecules are present on NETs and facilitate tumor cell extravasation and metastasis (14, 104). It was proposed that NETs have a strong ability to trap circulating tumor cells, thereby protecting them from immune system-mediated destruction and promoting tumor cell dissemination and adhesion at distant organs (105, 106). The premetastatic niche formation in the omentum is supported by increased neutrophil mobilization and NET formation, creating a conducive environment for the seeding of ovarian cancer cells (20). In an orthotopic model of ovarian cancer, depletion of IL8, granulocyte colony-stimulating factor (G-CSF), CXCL chemokine growth regulated oncogenes (GROα/CXCL1 and GROβ/CXCL2) in primary tumor cells incompletely decreased NET formation and chemotaxis, thereby inhibiting subsequent omental metastasis (20). NETs were also shown to enhance cancer metastasis by activating tumor-intrinsic TLR4/9-cyclooxygenase 2 (COX2) inflammatory pathways (107). Altogether these results suggest that cytokines cooperate with many factors to optimally regulate neutrophil recruitment and NET formation, which in turn enhance the inflammatory landscape of tumor, thereby contributing to tumor metastasis.

The metastasized liver tissues isolated from breast or colon cancer contain a high number of NETs. If NETs are detected in the serum of cancer patients, this could be a predicting factor for the occurrence of liver metastases at very early stages. NETs can attract cancer cells from established distant metastases. This cellular motility was mediated by the cancer cell-resident transmembrane NET-DNA receptor coiled-coil domain containing 25 (CCDC25) which activates the integrin-linked kinase (ILK)-β-parvin pathway and thus senses extracellular DNA release (18).

NETs are also involved in dormant cell reactivation thereby increasing metastatic events in distant organs (108). During chronic pulmonary inflammation, NETs awake dormant breast cancer cells and promote metastasis. Degradation of thrombospondin 1 (TSP1) and remodeling laminin-based extracellular matrix are important steps to awake the dormant cells. Consistently, activation of laminin receptor integrin α3β1 and transcriptional regulator yes-associated protein (YAP) signaling is required for NET-dependent activation of dormant tumor cells. Furthermore, integrin β1 is involved in the activation of FAK-ERK-MLC2-YAP signaling pathway, which also contributes to tumor survival and growth (108).

Cancer cells can also induce NETosis through other alternative mechanisms. Lewis lung carcinoma (LLC) cancer cells release a high amount of RNAs, which accumulate in the extracellular space and activate epithelial cells, thereby inducing NETosis mediated by proinflammatory cytokines, such as IL1β. NETs reduce the lung epithelial barrier, induce necrosis and the release of extracellular RNAs (17).

NETs can directly interact with T cells and suppress the anti-tumor immunity through metabolic and functional exhaustion, emphasizing the deleterious effect of NETs during all the evolutionary stages of the tumor process, including tumor growth, angiogenesis and tumor metastasis. Blockade of NETosis in combination with programmed death-ligand 1 (PD-L1) immune checkpoint inhibitors enhance the response rates of colorectal cancer metastasis by improving the function of exhausted CD8+ cells (109). NETs also modulate regulatory gene profiles in naïve CD4+ T cells, promoting their differentiation into regulatory T cells (Tregs). This crosstalk between NETs and Tregs was shown to contribute to liver carcinogenesis in non-alcoholic steatohepatitis (110). NETs are also observed in bladder tumors of patients who did not respond to radiotherapy and persistent disease post-radiotherapy, wherein an elevated neutrophil-CD8+ ratio was associated with worse overall survival (111).

## Cancer-Associated Thromboinflammation and NETosis

NETs provide a physical scaffold for thrombus formation by capturing platelets and red blood cells. Platelets are associated with NETs through binding of von-Willebrand Factor (vWF), fibronectin or immobilized fibrinogen (112). Interestingly, DNA was detected on the platelet surface of patients with systemic lupus erythematosus (113), indicating that platelets can directly bind DNA with histones in NETs, linking immune response to thrombosis. Growing tumors activate platelets by inducing uptake of tissue factor (TF)-derived extracellular vesicles (114, 115). Upon platelet activation, P-selectin is exposed to the surface which interacts with neutrophil-derived P-selectin glycoprotein ligand 1 (PSGL1), thereby promoting neutrophil-platelet interaction, subsequent neutrophil activation and NETosis (116). Thrombin-activated platelets primed neutrophils to NETosis in different *in vitro* and *in vivo* experimental conditions (116–118). Similar effects were observed when neutrophils were incubated with soluble P-selectin (116). In contrast, genetic or pharmacological blockade of P-selectin decreases NETosis (116). In clinical studies, increased P-selectin exposure on the activated platelet surface and increased soluble form of P-selectin are associated with venous thromboembolism (VTE) in cancer patients (119). Clark et al. showed that platelet-derived TLR4 induced platelet activation, platelet-neutrophil interaction and NETosis in the murine sepsis model (48). Platelet-derived high mobility group box 1 (HMGB1) can also activate neutrophil-resident TLR4 or binds to the receptor for advanced glycation end products (RAGE) on neutrophils, thereby inducing NETosis (118, 120). Furthermore, collagen and thrombin-activated platelets could also stimulate NETosis through HMGB1 (118). Thrombin-stimulated platelets also trigger MLKL-dependent necroptosis of neutrophils accompanied by NET release (121).

In the late stages of the breast carcinoma model, NETosis occurred concomitantly with the appearance of venous thrombi in the lung (97). Although this phenotype can be multifactorial, it is also closely linked to the role of neutrophils and platelets in the tumor microenvironment. Cancer predisposes neutrophils to generate NETs thus increasing platelet reactivity and hypercoagulability, thereby promoting primary tumor growth and stimulating tumor metastasis (97, 122, 123). NET formation is systematically correlated with the hypercoagulability state of cancer and thrombotic complications (16, 124, 125). During cancer progression, circulating DNA possibly induces the generation of thrombin, thereby activating the coagulation cascade (126). In an orthotopic mouse model of PDAC and human patients with PDAC, NET formation induces hypercoagulability by enhancing platelet aggregation responses through RAGE, DNA and TF release. Neutrophils isolated from RAGE-deficient mice had a lower ability to form NETs and circulating biomarkers of tumors and NETs were strongly reduced (127). Pancreatic cancer cells can stimulate NETosis through direct interactions with neutrophils or by priming platelets (128). Although blood clotting factors regulate neutrophil function (129), hypercoagulation was associated with the appearance of N2 protumoral neutrophils undergoing NETosis (130).

Apc^Min/+^ (multiple intestinal neoplasia) mouse has a point mutation at the adenomatous polyposis coli (Apc) gene, and it is considered to be a model for human familial adenomatous polyposis (131). In this intestinal tumorigenesis model, hypercoagulation was associated with neutrophil recruitment and NETosis and these observed effects were dependent on the engagement of the complement 3a receptor (C3aR) (130). In other transgenic mouse tumor models (RIP1-Tag2 insulinoma and MMTV-PyMT breast cancer models), neutrophil recruitment and vascular leakage were observed in the kidney. Furthermore, platelet-neutrophil conjugates were accumulated in the kidney of tumor-bearing mice, which consequently generated NETs. The accumulation of NETs in the vasculature increased the levels of proinflammatory molecules, such as intercellular adhesion molecule 1 (ICAM1), vascular cell adhesion molecule 1 (VCAM1), E-selectin, IL1β, IL6 and CXCL1 (98).

Neutrophils of patients with myeloproliferative neoplasms characterized with a constitutively activating mutation of janus kinase 2 (JAK2) are also primed to generate NETs. Inhibition of constitutively active JAK2 could abolish NET formation and decreased thrombosis, suggesting an important role of platelet-associated NET formation in cancer-associated thrombosis (132). Tumor cells can synthesize G-CSF which stimulates the proliferation of circulating neutrophils, and consequently increases NET formation in the growing tumors (97, 133). High levels of G-CSF and NET-associated thrombi were found in patients with ischemic stroke and underlying cancer (134), indicating the link between systemic NET formation and arterial thrombosis. Heparin-induced thrombocytopenia (HIT) immune complexes induce NETosis *via* interaction with Fcγ receptor FcγRIIa on neutrophils and through neutrophil-platelet association (135). On another hand, neutrophil FcγRs can reprogram neutrophils into antigen cross-presenting cells thereby inducing acquired anti-tumor immunity (136).

Recent studies implicated neutrophils and NETs as central players in coagulation, organ injury and thromboinflammation that were detected in severe cases of severe acute respiratory syndrome coronavirus 2 (SARS-CoV2) infection (137). SARS-CoV2 was able to induce ROS and IL8 secretion and activate NETosis in human neutrophils (138). The angiotensin-converting enzyme (ACE2) and active transmembrane serine protease 2 (TMPRSS2) are also involved in this process (137).

## Eosinophil Extracellular Traps

**Figure 2:**
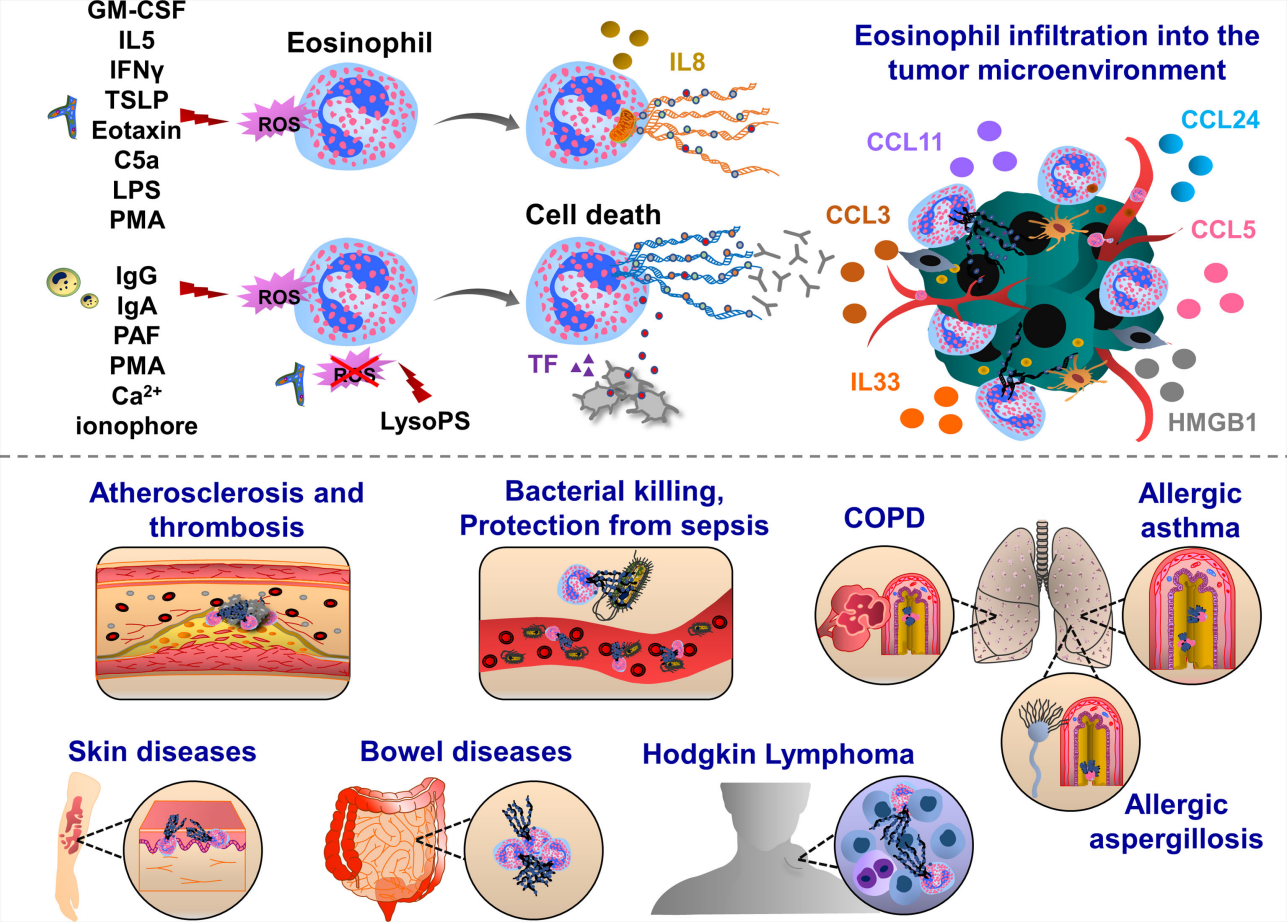
Pathophysiological functions of eosinophil extracellular traps (EETs). Upon IFNγ, GM-CSF or IL5 priming, eosinophils are activated by C5a, LPS, eotaxin/CCL11, PMA, Th2 alarmin or pathogens which trigger oxidative burst and the release of mitochondrial DNA into the extracellular environment. This process can be mediated by ROS-dependent and cell death-independent pathways. In response to IgG, IgA antibodies, PAF, Ca^2+^ ionophore, PMA and gram-positive bacteria *Staphylococcus aureus* eosinophils form ETs, which ultimately induce cell death in Nox-dependent manner. Along with the chromatin, various proteins are released from activated eosinophils such as citrullinated histone 3 (orange), major basic protein (MBP, green), eosinophil cationic protein (ECP, grey) and eosinophil peroxidase (EPX, red). EETs were observed in patients with respiratory diseases, such as eosinophilic asthma, COPD and allergic aspergillosis. Eosinophil EPX triggers the production of sputum anti-EPX and anti-nuclear autoantibodies in patients with severe eosinophilic asthma, inducing resistance to the anti-asthmatic treatments. In skin diseases, EET function was often associated with host defense thereby preventing bacterial dissemination and sepsis. EETs were also observed in ruptured arterial thrombi and atherosclerotic plaques. Upon interaction with blood platelets, eosinophils form EETs and eosinophil-specific MBP released together with chromatin web-like structures activate platelets, thereby inducing the formation of thrombi. Eosinophils infiltrate various tumor types and influence tumor growth and metastasis through the interactions with endothelial cells, macrophages, fibroblasts and T cells. EETs together with NETs have been found in patients with Hodgkin’s Lymphoma displaying fibrotic and thromboinflammatory tumor microenvironment.

Eosinophil extracellular trap (EET) formation was detected in different human diseases (**Figure 2**). EETs were observed in chronic obstructive pulmonary disease (COPD) sputum (139), and also in skin biopsies from patients with skin diseases such as Wells syndrome and bullous pemphigoids (140). In mouse models of atherosclerosis, eosinophils enhanced thrombus stability during arterial thrombosis (141). EET formation was detected in ruptured human atherosclerotic plaques and arterial thrombi (142). EETs were also observed in bronchial sections of a patient with allergic bronchopulmonary aspergillosis, which displayed eosinophil infiltrates in the mucus together with chromatolysis (143). Depending on the pathological conditions, EET formation is stimulated by different factors, released by pathogens, immune cells or cancer cells. In 2008, Yousefi et al., demonstrated that *in vitro* stimulation of eosinophils with LPS, C5a and or eotaxin/CCL11, by interferon gamma (IFNγ) and IL5-priming, induces the release EETs in a ROS-dependent manner. Interestingly, the majority of exposed EET DNAs are of mitochondrial origin (144). *In vitro* treatment of human eosinophils with thymic stromal lymphopoietin (TSLP) derived from epithelial cells induces the release of mitochondrial DNAs as well, and this process did not trigger cell death and was also dependent of Nox and β2 integrin functions (145). When eosinophils were primed with GM-CSF and activated with C5a, LPS or PMA, mitochondrial DNAs in EET were also observed, again excluding nuclear DNA and cell death in this process (146). However, EETs could be formed in the presence of cell death as well, involving extruded nuclear DNA and histones, indicating an alternative mechanism of EET formation (147). When eosinophils are exposed to *Staphylococcus aureus*, cells undergo nuclear disruption and cell death, leading to the release of nuclear DNAs and chromatin (145, 148). A similar process was observed when human eosinophils were stimulated with immunoglobulin IgG, IgA, a lipid mediator - platelet-activating factor (PAF), Ca^2+^ ionophore or PMA. In these experimental conditions, EETs were associated with histones and nuclear DNA. The release of nuclear EETs is mainly triggered by Nox-induced ROS production (147). However, depending on the experimental conditions, a ROS-independent mechanism was also observed when EET formation was induced by lysophosphatidylserine (LysoPS) through peptidyl arginine deiminase (PAD4)-mediated histone citrullination (149). Fungal species could also induce EET formation independently of ROS production, which occurred through CD11b binding and activation of Syk tyrosine kinase (143).

Eosinophils are specialized cells of the immune system, playing effector functions in allergic diseases, such as asthma (150). The percentage of EET-generating eosinophils was negatively correlated with lung function (151). Eosinophils express many receptors, adhesion molecules and integrins that allow their transit from the bone marrow to the blood (152–155). Eosinophil peroxidase activates and recruits dendritic cells to lymph nodes (156). The increased levels of eosinophil peroxidase and membrane-bound eosinophil granules in asthmatic patients lead to sputum rich in autoantibodies, such as anti-eosinophil peroxidase IgG, anti-nuclear, anti-double-stranded DNA and anti-histone antibodies (157). In allergic asthmatic diseases, peripheral blood eosinophils generate more EETs, when cells were challenged with LPS or IL5 *in vitro* (151). Challenging IL5 transgenic mice in a model of post-caecal ligation and intestinal puncture strongly enhanced eosinophil infiltration and EETs were observed in the intestinal tissues, protecting mice against sepsis (144). The authors found that in the colon and caecal tissues of mice and patients with Crohns disease, schistosomiasis and spirochetosis, extracellular DNA fibers were decorated with granular proteins such as major basic protein (MBP) and eosinophil cationic protein (ECP) (144). Besides these direct contacts, eosinophil MBP also enhances platelet activation inducing the release of bioactive molecules from α and δ granules or delivering activated TF, thereby contributing to the thrombus formation (141, 158, 159). Platelet-eosinophil interaction can induce EETs, triggered by IL5 release (141). EETs have also proinflammatory effects, subsequently activating epithelial cells to release proinflammatory cytokines such as IL6 and IL8 (151). In response to the opsonized *Escherichia coli*, activated eosinophils can release EETs, which had a strong bactericidal effect through a phagocytosis-independent mechanism (144).

Eosinophils and EETs were detected in the tumor tissues of patients with Hodgkin’s lymphoma (160). These patients had also increased expression of protease-activated receptor 2 (PAR-2) and nuclear p-ERK staining in cancer cells, which was detected together with abundant NETosis, fibrosis and TF-positive endothelium, pointing out the presence of tumor-associated inflammation and procoagulant phenotype (160). Eosinophils are also enriched in the circulating blood and tumor tissues in patients with other cancer types, such as colorectal, breast, ovarian, cervical, oral squamous and prostate cancer (161, 162). Eosinophils can transmigrate into the tumor microenvironment, following the interactions with endothelial cell-resident VCAM1 and ICAM1 (163). Cellular interactions of cancer cell-derived CCL24 and macrophage, fibroblast and eosinophil-derived CCL11 promote eosinophil recruitment to the tumor microenvironment (164–166). Cancer cell-derived chemokines (CCL3, CCL5) further support eosinophil migration (167, 168). Eosinophil-resident ST2, RAGE and TLR4 support migration towards the response to tumor necrotic cell alarmin mediators, IL33 and HMGB1 (163, 169–171). Furthermore, microbiota-released factors induce infiltration of eosinophils into the tumor microenvironment (172).

In summary, these results suggest that EETs play an important role in the activation and regulation of innate and adaptive immunity and are also involved in thromboinflammation. Based on EET DNA staining with eosinophil-specific markers, future studies are necessary to distinguish different sources of EETs. Precise, clinically relevant diagnostic tools will help to understand the phenotypic landscape of different cancers that are particularly enriched with eosinophils and propose more adequate therapeutic modalities.

## Dendritic Cell Extracellular Traps

**Figure 3:**
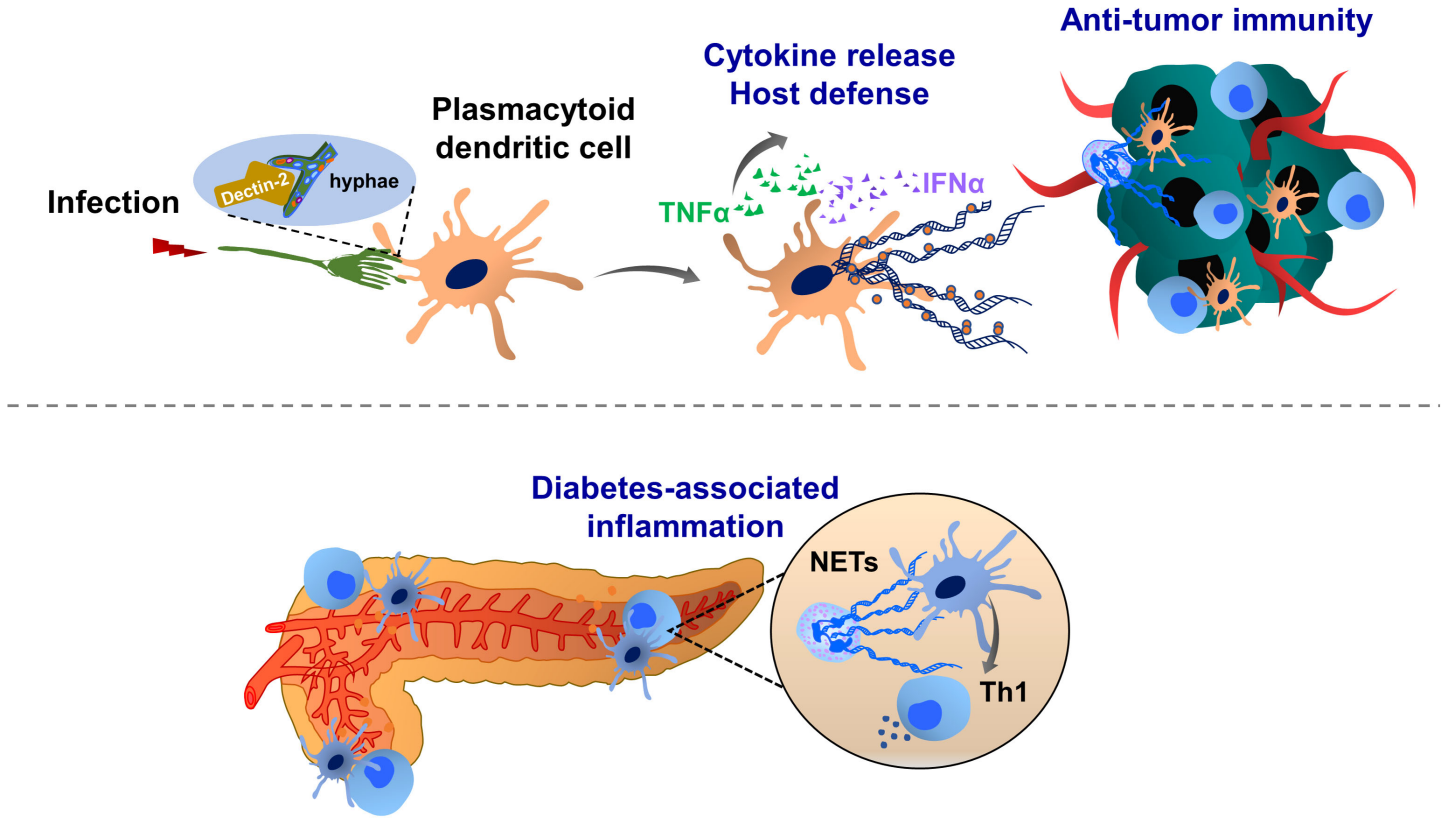
Molecular mechanisms of dendritic cell extracellular trap (DCET) formation and potential implications in cancer. A subset of dendritic cells, plasmacytoid dendritic cell-resident Dectin-2 interacts with the filamentous structure of pathogens (hyphae of *Aspergillus fumigatus*), thereby inducing ETs. These DCETs induce the release of cytokines such as TNFα and IFNα, eradicating pathogens. NETs may also activate dendritic cells, thereby triggering the production of IFNγ, which contributes to the pathogenesis of autoimmune diseases (diabetes). T cell priming by dendritic cells may contribute to the immunosuppression in the tumor microenvironment. The role of DCET in cancer remains elusive.

Dendritic cells can also form ETs (**Figure 3**). It has been shown that a subset of dendritic cells, such as plasmacytoid dendritic cells, can recognize the hyphae of *Aspergillus fumigatus* through Dectin-2 and this interaction induces ET formation (DCETs) with anti-fungal activity and release of cytokines such as TNFα and IFNα. DCETs contain nuclear DNA with citrullinated histone H3, which shows similar structures as NETs (173). Interestingly, NETs can activate dendritic cells and trigger IFNγ production, driving autoimmune pathologies (173–175). In diabetes and cancer, dendritic cells also prime T cell immunity (175, 176). However, only limited information is available to dissect the role of DCETs in this pathology. Therefore, further studies are necessary on whether dendritic cells may influence cancer progression by forming DCETs and acting on T cell-mediated immunosuppression.

## Monocyte and Macrophage Extracellular Traps

Monocytes and macrophages are critical components of the innate immune system, and play a key role in many pathological contexts, accumulating rapidly in the inflamed tissues (177). Monocyte and macrophage-extracellular traps (MoETs and METs) were visualized first time using scanning electron microscopy and immunofluorescence staining, detecting DNA fibers with specific dyes, such as DAPI, Hoechst, SYTOX, PicoGreen or TOPRO (49, 178, 179). ETs are generated from human peripheral blood monocytes (180, 181), human primary macrophages (182), human primary microglia and BV2 microglia (183), human placental macrophages (184), RAW 264.7 murine and U937 human monocyte-macrophage cells (50), THP-1 macrophage-like cells (184, 185), human glomerular macrophages (186), mouse J774A.1 macrophage-like cells (187), bovine (178, 179, 188) and caprine (189) monocytes. ETs in these cell types are composed of nuclear origin DNA fibers with MPO, citrullinated histone H3, elastase, MMP9, MMP12 and lysozyme (142, 180, 182, 187, 190). *Besnoitia besnoiti* is a cyst-forming apicomplexan protozoan parasite that causes bovine besnoitiosis which is traditionally endemic in Africa and Asia and also spreads in Europe. METs were detected when bovine or other mammalian species were exposed to pathogens tachyzoites of *Besnoitia besnoiti* (188). A highly pathogenic coccidian parasite *Eimeria ninakohlyakimovae* causes severe hemorrhagic typhlocolitis and *in vitro* exposure of caprine monocytes to sporozoites, sporocysts or oocysts could also induce MoETs (189). Non-infected monocytes derived from human peripheral blood can also form ETs.

**Figure 4:**
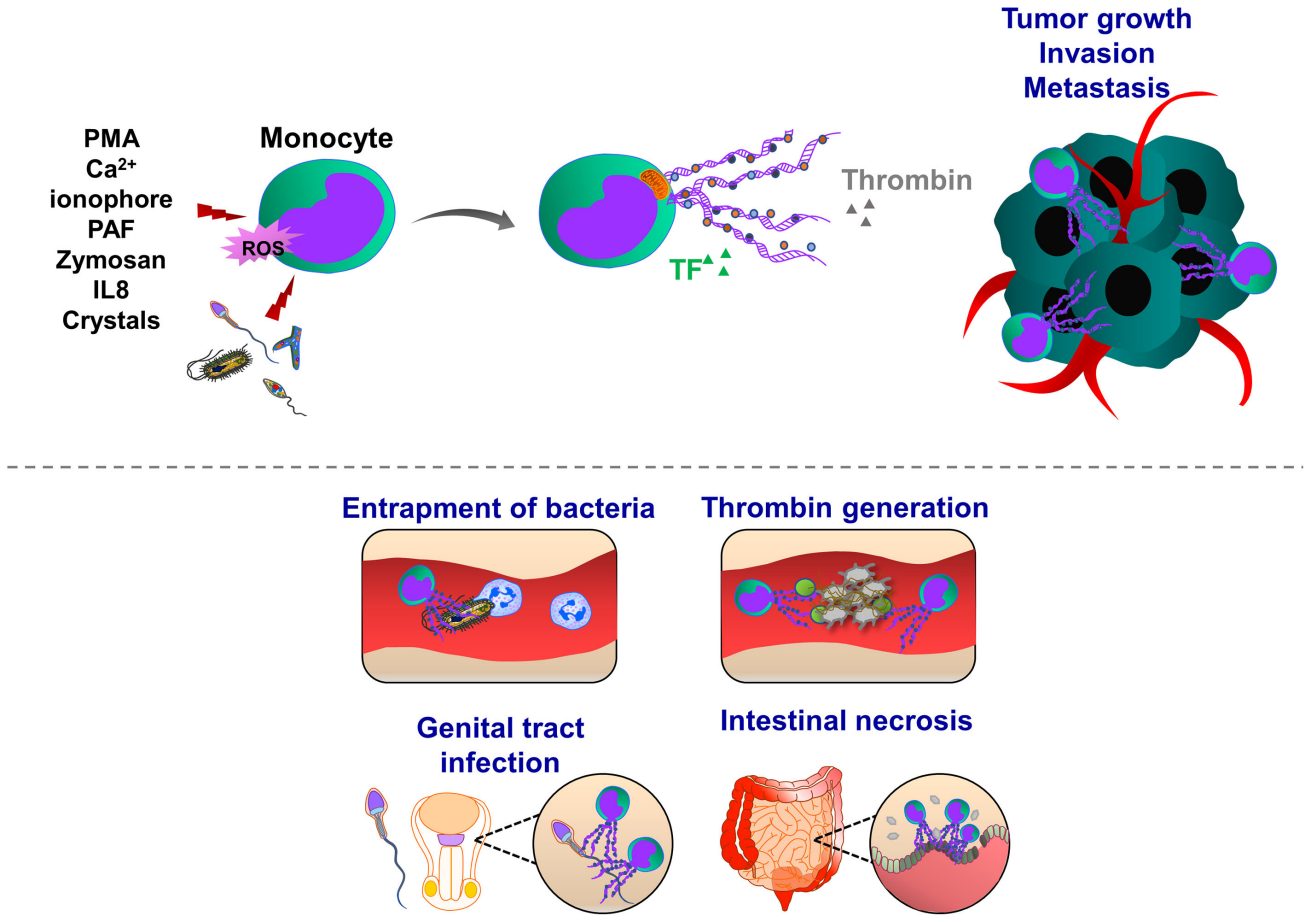
Pathophysiological functions of monocyte extracellular traps (MoETs). During inflammation, ETs can be induced in activated monocytes, which occurs in Nox-dependent manner. Monocyte can release DNA from the nucleus and mitochondria, containing similar ET components such as histone 3, MPO, lactoferrin and elastase. During infectious and inflammatory processes, MoETs entrap pathogens, stimulate phagocytosis and also accelerate the thrombin generation, thereby enhancing procoagulant phenotype. During male genital tract infections and inflammation, spermatozoa induce ET formation in monocytes, which in turn inhibit their motility and reproductive system function. Crystal-induced MoETs have been suggested to contribute to a dysfunction of the intestinal barrier and intestinal epithelial cell necrosis ultimately leading to systemic inflammation.

**Figure 5:**
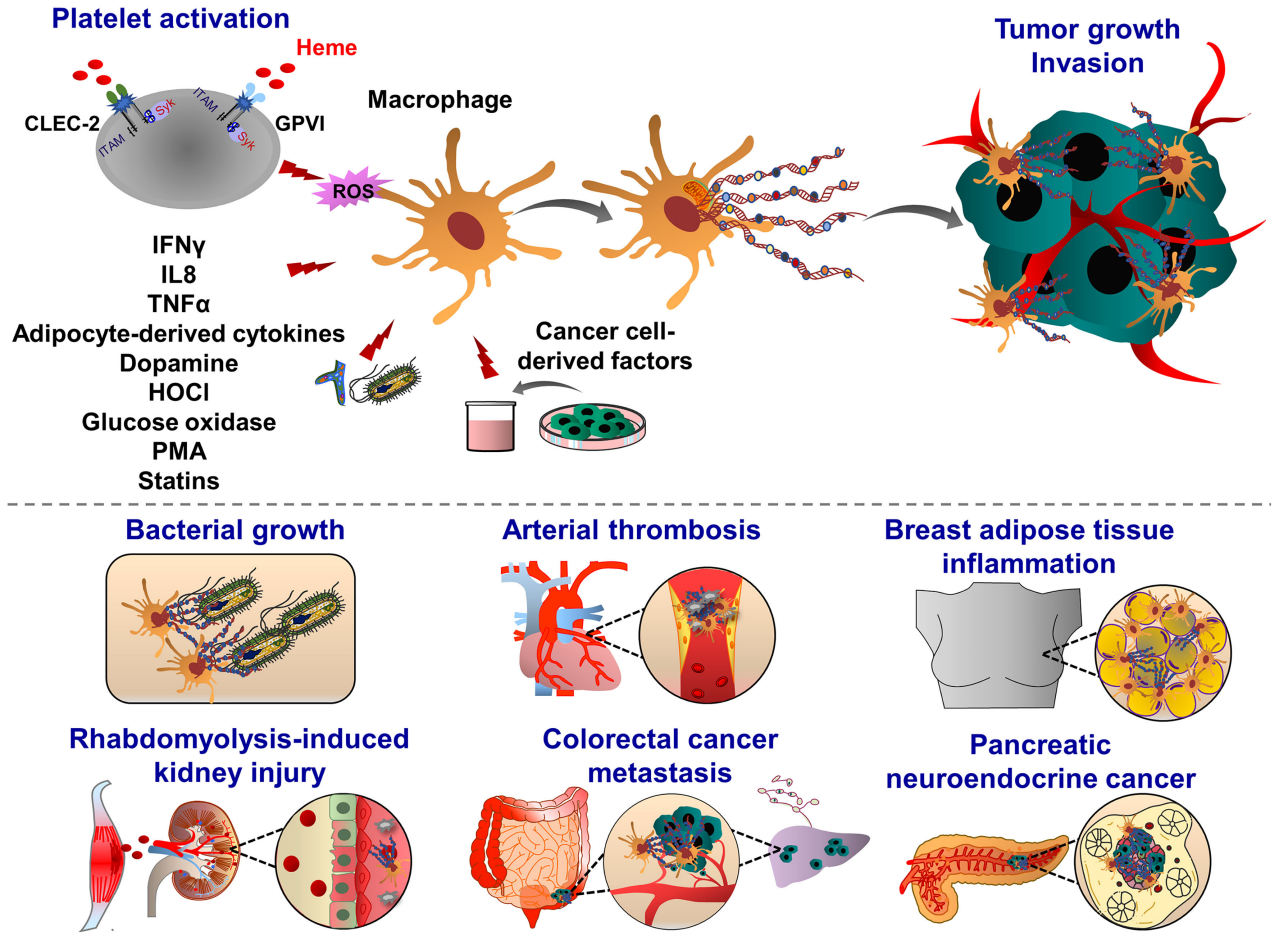
Pathophysiological functions of macrophage extracellular traps (METs). Macrophages emit ETs following exposure to the pathogens (yeast, bacteria) and inflammatory mediators (glucose oxidase, dopamine, i.e. IFNγ, IL8, TNFα and HOCl). During organ injury, heme-activated platelets induce METosis by increasing the levels of ROS and histone citrullination. Heme binds to platelet receptors CLEC-2 and GPVI, and activates the (hem)ITAM-signaling pathways, triggered by Syk kinase and PLCγ activation, which ultimately promote METosis. METs are composed of mitochondrial or nuclear DNA and different proteins, amongst them are citrullinated histone 3, MPO, elastase, MMP-9, MMP-12 and lysozyme. Although METs display various bactericidal proteins, exposure to bacterial pathogens such as *Mycobacterium massiliense* triggers MET release and capture of bacteria, METs can also enhance bacterial growth. METs are also involved in the progression of coronary atherosclerosis and thrombosis as they are abundant components of late or organized thrombi and may contribute to the thrombus growth along with ETs released from other immune cells. Proinflammatory cytokines derived from adipocytes may also induce MET formation, indicating the potential implication of METs in obesity. METs are also found in solid tumors, such as pancreatic neuroendocrine and colon cancer. Tumor cell-derived growth factors and cytokines prime and activate macrophages to release ETs. In their turn, METs interact with cancer cells, further increasing their motile, migratory and invasive potential.

Similar to the induction of NETosis, ET formation in monocytes can be triggered by PMA, A23187, PAF, or zymosan (180), (**Figure 4**). MoETs contained MPO, lactoferrin, citrullinated histone H3, and elastase. The mitochondrial and nuclear origin of DNAs was confirmed with PCR and immunofluorescence staining of ETs. Although blockade of Nox activity in monocytes could inhibit MoETosis, this process was not affected upon treatment with MPO inhibitor 4-aminobenzoic acid hydrazide (ABAH), indicating that MoETosis is ROS-dependent, but MPO-independent in this experimental condition (180). In another study, exposure of macrophages to the yeast and bacteria-induced MET formation in J774A.1 mouse macrophages or primary mouse peritoneal macrophages, such an effect was not observed upon treatment with PMA, H_2_O_2_ and IFNγ, indicating an alternative way of ROS-independent METosis (187), (**Figure 5**). However, others contrarily showed that the proinflammatory substances stimulate ROS, which subsequently induces the formation of METs (178, 182). Heme is one of the strong inducers of ROS production in immune cells (191). Elevated heme production and METs were frequently detected in patients with liver and kidney ischemic injury. In mice challenged with rhabdomyolysis-induced kidney injury, heme-activated platelets could induce METosis by increasing ROS production and histone citrullination (185). A follow-up study showed that hemin interacts with platelet-resident C-type lectin-like receptor 2 (CLEC-2) and Glycoprotein VI (GPVI), thereby inducing platelet activation and consequent MET formation (192, 193). Hemin interaction with platelets could enhance the enzymatic activity of Syk kinase and phospholipase Cγ (PLCγ). This concept was proved by using knockout mice with CLEC-2 or FcRγ deficiency in which attenuated renal dysfunction, tubular injury, and reduced METosis were observed, highlighting an important role of platelet (hem)ITAM-signaling in METosis (193). In atherothrombotic plaques isolated from patients with coronary thrombosis, both METs and NETs were detected. METs were more robust in intact lipid plaques and associated thrombi. Although NETs were also detected at the early step of thrombosis, METs were observed at the advanced stage in the organized thrombi (142). METs can generate thrombin and increase procoagulant activity, implying an important thrombogenic function (180).

Adipose tissues isolated from obese patients contain a high number of macrophages which are infiltrating around dead adipocytes and forming a macrophage trap-like structure (194, 195). This tissue structure is frequently associated with increased levels of inflammatory cytokines, such as tumor necrosis factor α (TNFα), IL1β, and COX2 (196, 197). Exposure of RAW 264.7 macrophages to TNFα increased the levels of PAD2 and extracellular chromatin scaffold formation, indicating that inflammatory mediators released from adipocytes may stimulate METosis in the mammary fat pad environment. Interestingly, NET-specific PAD4 was absent in METs in the mammary fat pad (198). Macrophage activation is often correlated with a bad prognosis in many cancer types, including breast cancer, implying inflammation, accelerated tumor progression and metastasis (199). Furthermore, adipose tissue inflammation and obesity are also associated with an increased risk of breast cancer recurrence. MET formation may possibly correlate with these pathological signs and the severity of breast cancer. Recently Xu et al., identified several sources of NETs and METs in tumor tissues isolated from patients with pancreatic neuroendocrine cancer (200). The patients with high levels of NETs and METs have a postoperative cancer recurrence (200), indicating that these ETs may generate anti-cancer resistance mechanisms, leading to the cancer relapse.

Recent studies demonstrated METs could enhance *in vitro* invasion of HCT16 and SW480 colon cancer cells (201). Interestingly, exposure of macrophages to the conditioned cancer cell culture medium induced MET formation in a PAD2-dependent manner, indicating a positive feedback mechanism between MET and colon cancer cells. After PAD2 inhibitor treatment, the reduced MET formation was observed and consequently, the number of liver metastases was also decreased in mice, highlighting the contribution of METs to the tumor metastasis (201). In line with this, increased levels of tumor-associated METs were observed in human colon cancer tissues, predicting the poorest prognosis for colon cancer patients (201). Further studies are required to investigate how METs may induce motility, migration and invasion of colon cancer cells thereby leading to tumor metastasis.

Besides several experimental pieces of evidence showed that METosis has similar features as NETosis (49, 180, 182, 202, 203). Pathogens (bacteria, protozoa, fungi) and also spermatozoa, induce both MoET and NET formations, triggered by IL8-mediated activation of monocyte or neutrophils, respectively (12, 187, 202–204). In line with this, exposure of intestinal cells to the crystals of sevelamer, polystyrene sulfonate or cholestyramine could induce dysfunction of the epithelial cell barrier, associated with MoETosis and NETosis (205). Imbalanced gut microbiota and disrupted epithelial barrier represent an early subclinical phase of colitis-associated cancer (206). It could be interesting to evaluate whether the presence of MoETs or METs in these pathological conditions may represent a prognostic and diagnostic marker, thereby helping an earlier intervention.

## Mast Cell Extracellular Traps

**Figure 6:**
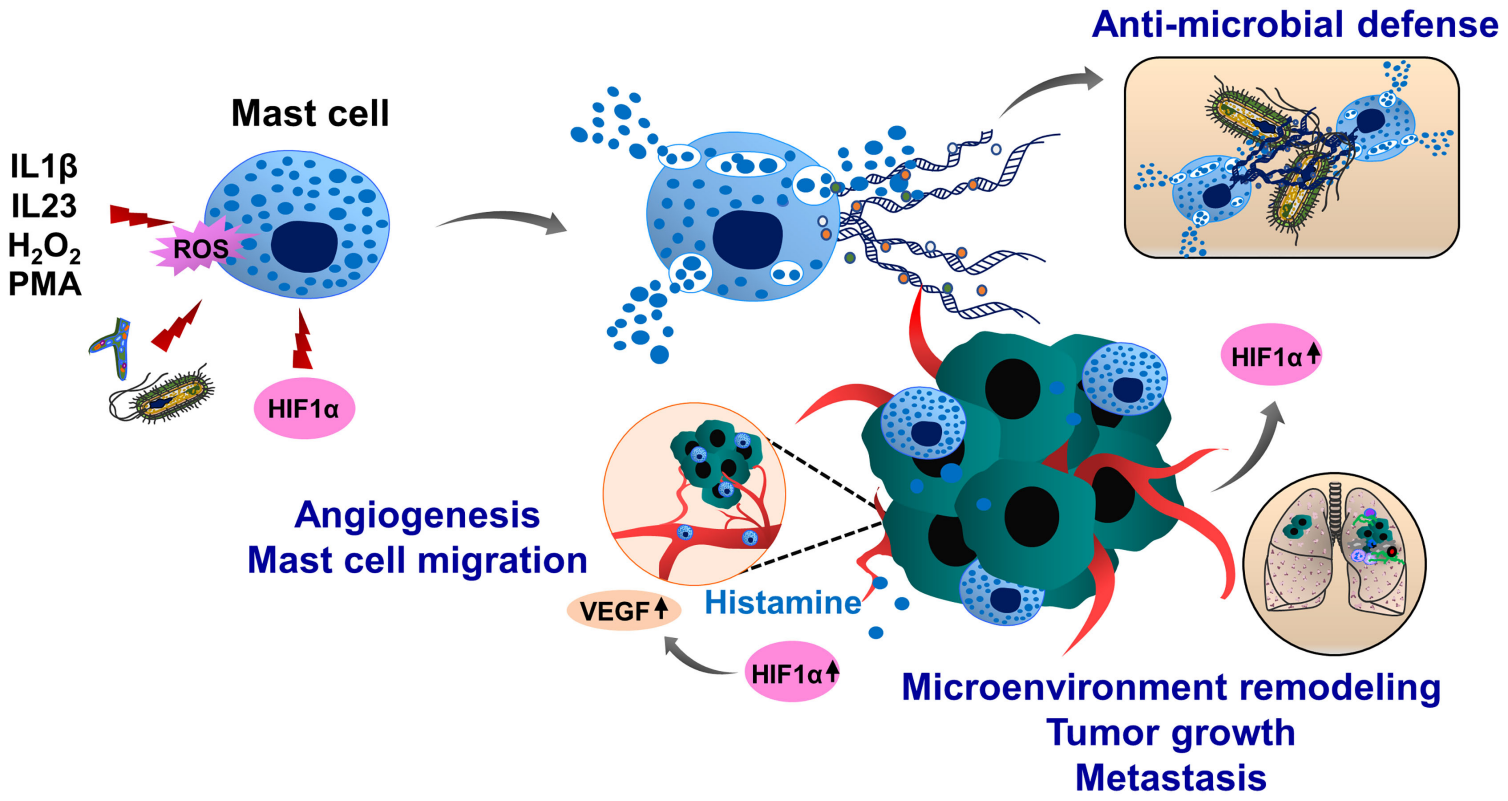
Molecular mechanisms of mast cell extracellular trap (MCET) formation and potential implication in cancer. Another type of myeloid cells, mast cells also form ETs (MCETs). This response can be induced by the pathogens (bacteria, fungi), PMA, H_2_O_2_, cytokines and chemokines and occurs in a ROS-dependent manner. Although MCETs contain DNA and histones (orange), these ETs also entail granule derived tryptase (green) and anti-microbial peptide CRAMP/LL-37 (grey). Potentially, MCETs could play a role in cancer, as mast cells infiltrate the tumor microenvironment and promote invasion and metastasis of tumors. Furthermore, enhanced histamine levels activate and increase mast cell HIF1α and VEGF activity, contributing to tumor angiogenesis. HIF1α has been reported to enhance MCET formation in response to appropriate stimuli. In line with this assumption, hypoxic conditions in the tumor microenvironment could increase HIF1α levels in mast cells, thereby contributing to the mast cell activation and MCET formation and possibly contributing to the tumor progression and metastasis.

Mast cells have limited phagocytic activity compared to other immune cell types, therefore, the anti-microbial and anti-bacterial activity of these cells is mainly ensured by degranulation and release of anti-microbial peptides, such as defensins, proteases and cathelicidins (207, 208). Following exposure to pathogens, mast cells degranulate and release mast cell-extracellular traps (MCETs) in a ROS-dependent manner (209). MCETs are composed of classical components of ETs, such as DNA and histones and had inhibitory effects on bacterial growth. In contrast to other ETs, MCETs contain unique components such as mast-cell granule proteins tryptase and cathelicidin-related anti-microbial peptide (CRAMP/LL-37), (**Figure 6**). Therefore, effective MCET degradation was possible using the mixture of DNAse I and tryptase-degrading enzymes (209). Interestingly, HIF1α can induce MCET formation thereby enhancing the anti-microbial activity of mast cells (210). During tumor growth, mast cells infiltrate into the growing tumors and remodel the tumor microenvironment by regulating immune and inflammatory reactions. In the melanoma cancer model, HIF1α together with histamine induces mast cell migration by increasing vascular endothelial growth factor (VEGF) production and consequent tumor angiogenesis (211). Tumor-infiltrating mast cells also potentiate tumor cell invasion and metastasis by interacting with cells in the tumor stroma (212–214). However, it is an open question whether mast cells can generate MCETs in response to the tumor microenvironment and how this process may influence cancer progression and metastasis.

## Basophil Extracellular Traps

**Figure 7:**
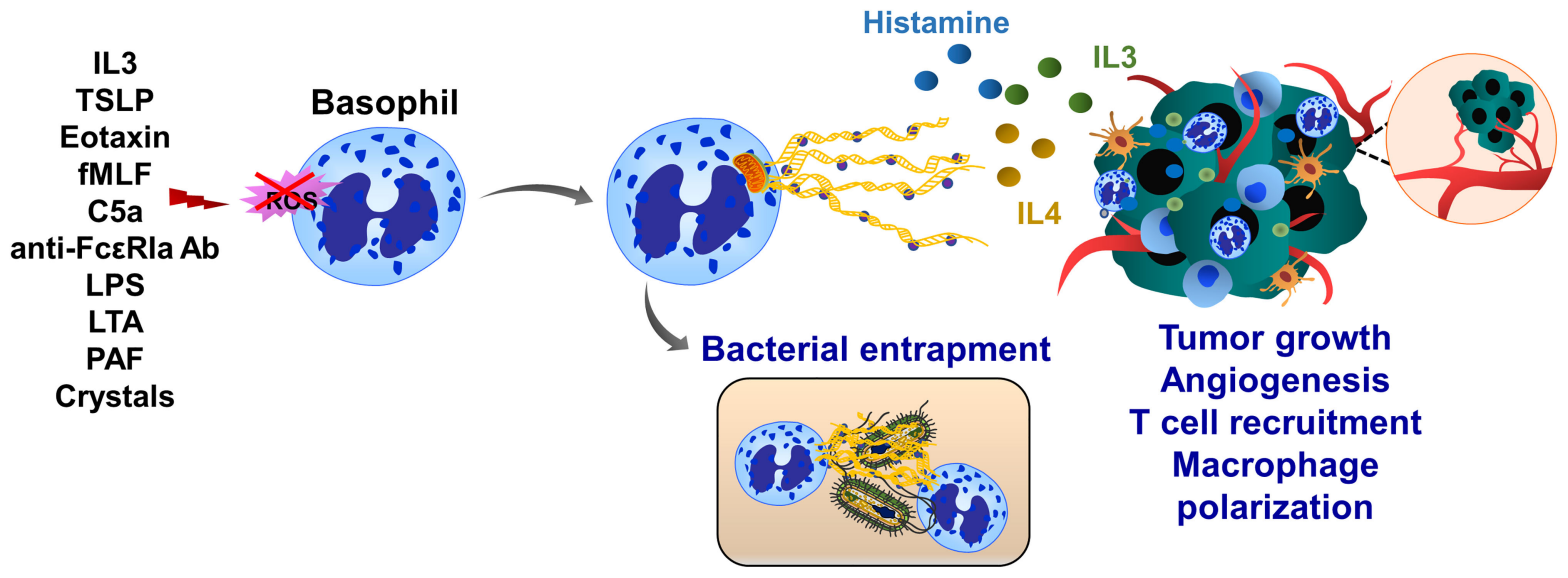
Basophil extracellular traps (BaETs). Basophils synthesize several proinflammatory and proangiogenic factors such as VEGF, angiopoietin and cysteinyl leukotriene C. Basophils also produce inflammatory cytokines, such as IL3 and IL4 upon activation with cancer cells. Following activation with complement factor 5a receptor or FcγRI basophils release ROS and form ETs, which are composed of mitochondrial DNA and generated in a Nox-independent manner. Besides inflammation, basophils regulate T cell recruitment and anti-tumor immunity. Future studies are required to address the role of BaETs in several steps of tumor progression, including primary tumor growth, angiogenesis and tumor metastasis.

Basophils are associated with inflammation, infection, immune defense and allergic response. Human basophils synthesize several proinflammatory and proangiogenic factors such as VEGF, angiopoietin and cysteinyl leukotriene C (215). Basophils also release histamine and produce IL4 and IL13 when cocultured with A549 lung carcinoma cells (216). Basophils produce ROS and form ETs upon IL3 priming and activation of complement factor 5a receptor or FcγRI (217). Although basophil extracellular traps (BaETs) contain mitochondrial DNA but not nuclear DNA, ET formation in basophils occurs in a Nox-independent manner (218), (**Figure 7**). Basophils are present in the tumor microenvironment of human pancreatic and lung cancers and can induce inflammation-related skin tumor growth (219). Lung-resident basophils contribute to pulmonary development and promote M2 polarization of local macrophages (220). Besides their protumor functions, basophils located in melanoma cancer elicit anti-tumor properties by promoting tumor rejection *via* chemotaxis and infiltration of CD8+ T cells (221). Although these studies linked basophils to cancer development, the molecular mechanisms of BaET formation in cancer tissues and the consequent impact on tumor cell function have not been elucidated.

## T Cell Extracellular Traps

**Figure 8:**
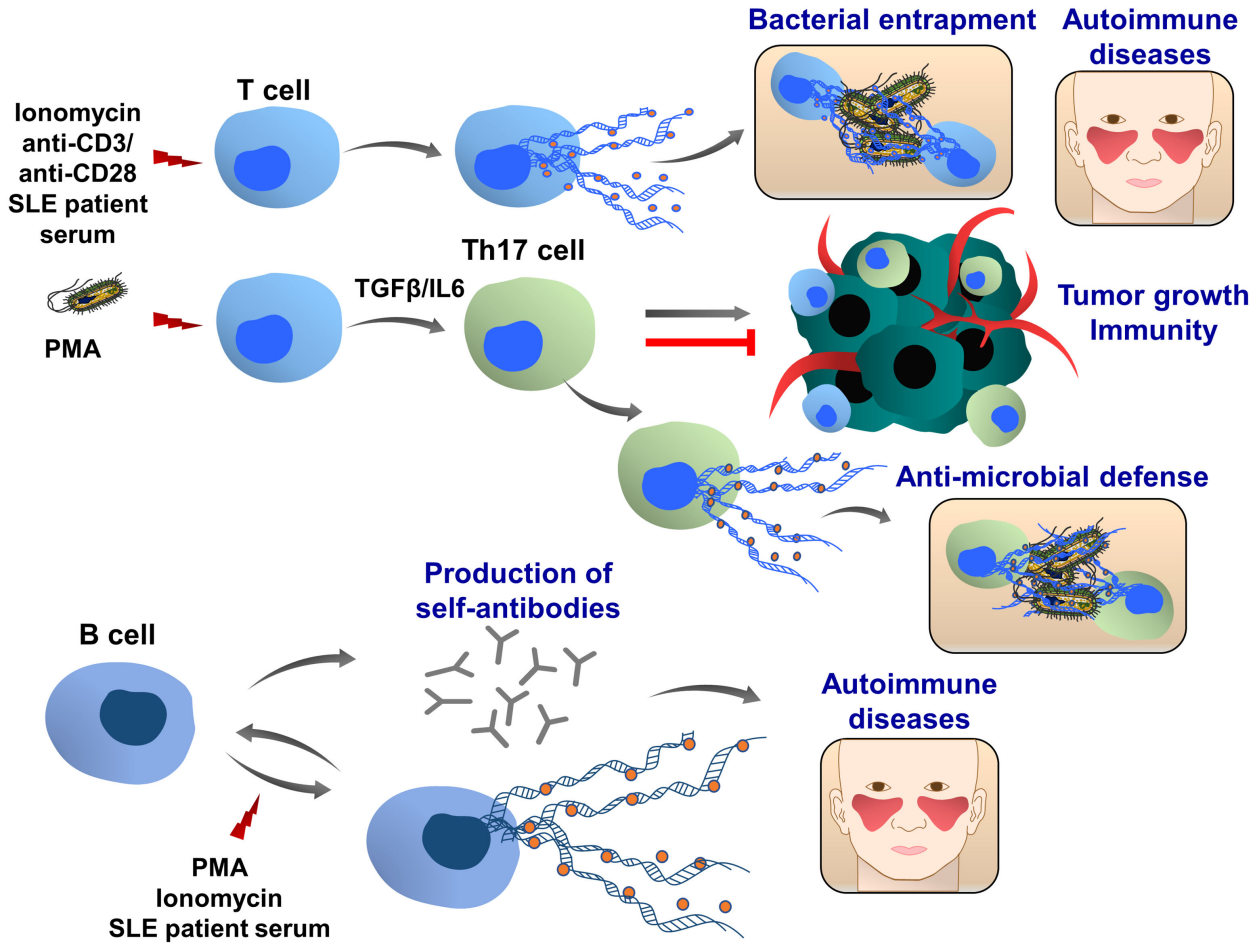
T and B cell extracellular traps (TCETs and BCETs). Under certain experimental and pathophysiological conditions, ie stimulation with ionomycin or systemic lupus erythematosus patient serum, T cells can release ETs. A similar phenomenon was observed in CD8+ cells following the stimulation with anti-CD3/anti-CD28 antibodies, engaging T cell receptors. In presence of TGFβ and IL6, the naïve CD4+ T cells differentiate to the IL17 producing T cells (Th17 cells), which are associated with chronic inflammation and autoimmune diseases. In response to bacterial infection, this T cell population releases ETs, which are composed of DNA, histones and bactericidal proteins, leading to the entrapment of bacteria. Depending on the pathophysiological conditions Th17 cells can either promote or attenuate tumor development and metastasis. Further studies are required to understand whether cancer cells and tumor microenvironment may induce TET formation, which in turn can modulate tumor growth, metastasis and cancer immunity. B cells can release extracellular traps upon stimulation with PMA and ionomycin. BCETs were also observed after treatment with serum isolated from a systemic lupus erythematosus patient, indicating that soluble factors in the serum induce the DNA release and possibly BCETs could be involved in the pathogenesis of the disease. BCETs may serve as self-antigens that are recognized by other B cells, followed by autoantibody production and disease progression. Their role in cancer remains elusive.

Th17 cells belong to the CD4+ T-cell subset characterized by the production of IL17 and are considered an important mediator of inflammation, tissue homeostasis and cancer development (222, 223). Depending on their sensitivity to the microenvironmental stimuli, including cytokines and transcription factors, Th17 cells either enhance tumor growth and metastasis or promote anti-tumor immunity (224, 225). Like neutrophils, Th17 cells also play an important role in host defense against bacteria and pathogens (226). Recently, T cell extracellular trap (TCET) formation was observed, which was induced in this subset of activated T cells, releasing histone-rich TCETs in conjunction with anti-microbial proteins, thus trapping and killing bacteria (227), (**Figure 8**). When peripheral blood T cells were isolated from healthy individuals and stimulated with the serum of patients with systemic lupus erythematosus, ET formation was observed (228), as well as after stimulation with anti-CD3/ anti-CD28 of CD8+ cells (229). Future studies are important to evaluate whether T cells can also form TCETs in response to tumor cells and tumor microenvironment and how TCETs may influence tumor growth, progression and tumor immunity.

## B Cell Extracellular Traps

Only very limited results showed that B cells can also release extracellular traps (BCETs), (**Figure 8**). Similar to TCETs, B cells were stimulated with the serum from patients with systemic lupus erythematosus and BCET formation was detected (228). It was hypothesized that BCETs could be a constant source of self-antigens for autoreactive B cells stimulating the production of antibodies (230).

## Other Sources of Extracellular DNA

### Endothelial Cell-Derived Extracellular DNA

DNA structures extruded from endothelial cells were observed during arterio-arterial embolization, a pathological condition occurring following cholesterol crystal-induced embolism in the kidney (231). Cholesterol crystal embolism is mobilized from an atherosclerotic plaque, followed by vessel obstruction, ischemia and organ failure (232). Studies by Shi et al., showed that injection of cholesterol crystals into the artery of the mouse kidney generates a thromboinflammatory environment with the presence of intravascular thrombi, composed of platelets, fibrin, neutrophils and extracellular DNA (231). Using *in vitro* cell culture experiments, exposure of neutrophils to cholesterol crystals or the supernatant of cholesterol crystal-activated platelets induced neutrophil necrosis and the release of chromatin and DNA to the cell culture supernatant (231). Interestingly, exposure to increasing doses of cholesterol crystals also induced necrosis of glomerular endothelial cells and consequent DNA release (231).

The vasculature of metastatic organs is frequently damaged and metastases can induce cell death (233). Necroptic cell death and subsequent DNA release occur in endothelial cells, involving RIPK1, RIPK3 and MLKL cell death signaling pathways. Tumor cell-induced endothelial necroptosis was shown as an important mediator of tumor cell extravasation and subsequent tumor metastasis (234). Further experiments need to be performed whether under certain conditions endothelial cells may also undergo ETosis.

### Platelet-Derived Extracellular DNA

Platelets lack nuclear DNA and the amount of mitochondrial DNA is very limited, due to the few numbers of mitochondria per platelet (235). Theoretically, accumulated platelets at the injury sites may release mitochondrial DNA upon platelet activation (236, 237). This extracellular DNA may be contributed to immune cell-derived ETs, and further amplify cancer-associated thrombosis, thromboinflammation and tumor progression. Further studies are important to establish the role of platelet-derived ETs in these processes.

### Cardiomyocyte-Derived Extracellular DNA

Cancer is associated with cachexia, vascular and metabolic dysregulation of the heart (238, 239). Cardiomyocytes possibly are a major source of extracellular DNA in patients with myocardial infarction (240, 241). Microvesicles and exosomes released from cardiomyocytes also contain extracellular DNA (242). Due to the limited experimental evidence, further studies are necessary to investigate the role of cardiomyocyte-derived extracellular DNA, analyze metabolic and DNA contents in patients with cancer and establish the contribution of ETs in myocardial infarction and cancer-associated heart dysfunction.

### Tumor Cell-Derived Extracellular DNA and Horizontal Transfer of DNA

The blood plasma levels of extracellular DNAs are increased in human patients with breast, melanoma, pancreatic and colon cancers, which are directly extruded by cancer cells (4, 10, 243). Circulating extracellular DNA can interact with several molecules, exposed on the surface of blood cells, leading to the penetration of DNA (244, 245). Histones and complement factors directly bind and capture DNA (246–248). DNA can also be transferred to the exosomes and microparticles and secreted to the circulation. Indeed, circulating microvesicles isolated from the blood cancer patients contain fragments of mutated genes, such as phosphatase and tensin homolog (PTEN), p53 and KRAS (249–251). Cai and colleagues found that BCR/ABL hybrid genes can be transferred from chronic myeloid leukemia cells to the HEK293 and neutrophils, increasing DNA coding mRNA and protein levels (252). Similar results were observed with vascular smooth muscle cells and leukocyte-derived extracellular vesicles delivering the angiotensin receptor type 1 (AT1R) gene DNA to HEK293 cells and sex-determining region Y (SRY) DNA into the endothelial cells (253, 254).

### Endogenous DNAse

DNAse enzymes are divided into two major families, DNAse I and DNAse II. Although DNAse I is found in exocrine gland secretions and blood, DNAse II derives from lysosomes/phagolysosomes (255). Regarding the sources of circulating DNA, it was assumed that tumor cells in cancer patients shed and release DNA into the bloodstream and this correlated with the pathogenesis of the disease (5, 256). In line with this, DNAse I levels in cancer patients are elevated during remission, and after successful interventions and decreased during cancer progression and metastasis. Furthermore, failure of DNAse levels to increase in response to treatment was correlated with poor prognosis (257, 258). However, DNAse activity in the blood was found to differ between healthy subjects and cancer patients and also varies between cancer types and stages of cancer (257–261). Indeed, decreased DNAse activity was found in patients with malignant lymphoma, gastrointestinal and prostate cancer (260, 262, 263), while the levels of DNAse activity were higher in breast cancer patients compared to the control (264). The physiological relevance of DNAse function in NETosis was proved in knockout mouse models. Mice with DNAse I and DNAse I like-3 enzyme deficiencies developed NETosis with intravascular clots and obstructed blood vessels which resulted in tissue damages of vital organs, such as the lung, liver and kidney (265). In humans, genetic mutations of DNAse are associated with autoimmune diseases such as systemic lupus erythematosus (266). DNAse X/Apo10 antibodies were found in patients with oral squamous cell carcinoma, indicating gene inactivation of DNA X in this type of tumor (267). A therapeutic strategy based on the delivery of transgenic vectors expressing DNAses was proposed to target DNA destruction or apoptosis. In 2011, Karli Rosner suggested an anti-cancer therapeutic approach based on human recombinant DNAse I. According to his approach, the replacement of apoptosis-activated endogenous DNAses with human recombinant DNAse I might help to bypass cancer defense mechanisms, increasing the killing efficiency of chemo and radiotherapy-resistant tumor cells (268). Since the inactivation of endogenous DNAse X gene was found in many tumor cells types, the strategies to restore the levels of DNAse X in cancer cells could be an important targeted therapy (267, 269). Delivery of vectors encoding several DNAses under one common promoter into the cancer cells could successfully induce apoptosis (268, 269).

Based on these findings, gene therapy was developed in a mouse model of colorectal cancer in which an adeno-associated virus (AAV) vector was used to express DNAse I in the liver, thereby suppressing the development of hepatic metastases. After AAV-DNAse I treatment, NETosis was inhibited in the tumor tissues with restored local immune responses by increasing the percentage of CD8+ T cells (270).

### Exogenous DNAse I

Recombinant DNAse I has been successfully used as an anti-cancer agent and studied as a prognostic/diagnostic marker during cancer therapy. In 1961, de Lamirande determined the effect of DNAse and RNAse in mice bearing Ehrlich ascites carcinoma for the first time (271). After tumor cell implantation, daily injection of DNAse I could increase the survival rate of treated mice, but RNAse treatment did not affect mouse survival. A hypothesis was proposed, which included the uptake of DNAse into cancer cells, followed by necrosis and digestion of nuclear DNA (271). In other studies, the daily injection of RNAse and DNAse alone or in combination could enhance nuclease activity of blood plasma of tumor-bearing mice, and decrease the levels of extracellular DNA, back to the levels of control animals. Degradation of DNAs in the blood plasma was associated with reduced metastasis of LLC and hepatoma A–1 (HA-1) cancer cells (272–274). In the model of LLC, exogenous DNAse treatment not only inhibited metastasis but also increased DNAse activity in the blood, destroying extracellular DNA in the circulation of tumor-bearing mice by targeting tumor-associated DNA fragments such as short and long interspersed retrotransposable elements (SINEs and LINEs) and also oncogenic sequences (274, 275). Furthermore, daily intramuscular injection of bovine pancreatic DNAse I in LLC tumor model could also strongly decrease metastasis (276). In mouse models of melanoma, lymphosarcoma or pancreatic cancer, DNAse I treatment had also strong anti-tumor and anti-metastatic effects by destroying extracellular DNA (275, 277, 278). Bovine pancreatic DNAse also displayed anti-metastatic effects inhibiting the number of lymph nodes and lung metastasis in mouse models of leukemia and lymphoma cancers. Although bovine pancreatic DNAse I could inhibit the proliferation of several cancer cell types (Calu-1, SK-MES-1, HeLa, HEp-2 and L-929), it did not affect the peripheral blood mononuclear cells and fibroblasts (279). Combined treatment of DNAse I with proteases such as papain, trypsin or chymotrypsin led to a significant decrease of DNA content in the blood serum of rats, and no anti-tumor effects were observed in mice treated with proteases alone (280).

Pancreatic cancers belong to the group of diseases which affect both the endocrine and exocrine functions of the pancreas (281). The tumor microenvironment is instrumental in pancreatic tumor growth and metastasis. Although some mechanisms reflect tumor cell-autonomous processes, most require the interaction of tumor cells with tumor microenvironment, including endothelial cells, fibroblasts, and immune cells (282). In addition, chronic inflammation, thromboembolism and hypercoagulability are known as key features of PDAC (283, 284). Interestingly, DNAse I treatment of pancreatic cancer cells could strongly decrease tumor cell adhesion and migration, although tumor cell proliferation was not affected. In the orthotopic pancreatic cancer model, DNAse I treatment also strongly inhibited tumor burden and tumor metastasis to the liver and diaphragm, confirming the important pathological role of extracellular DNA in pancreatic cancer. Elevated CXCL8 secretion was detected in the medium of pancreatic cancer cell lines derived from liver metastases, in comparison with immortalized pancreatic ductal epithelial cells. Furthermore, the treatment of pancreatic cancer cells with recombinant CXCL8 could strongly increase extracellular DNA production (285). CXCL8 also induces ET formation in neutrophils, thereby enhancing cancer malignancy (14, 44, 96). DNAse I treatment strongly reduces ETs, and also the percentage of polymorphonuclear neutrophils that released observable ETs (286). Pancreatic tumor-bearing mice had also increased levels of NETs, and more rapid thrombotic occlusion in the injury model of jugular vein. DNAse I did not affect thrombotic occlusion in control mice, but protected tumor-bearing mice from enhanced venous thrombosis (287). These results suggest that enhanced NETosis contributes to thrombosis in pancreatic cancer.

Interestingly, DNAse I can also inhibit thrombosis independently of neutrophils. In the mouse model of cholesterol crystal embolism, *in vivo* depletion of circulating neutrophils in the peripheral blood did not influence the severity of disease, but DNAse I treatment significantly inhibited the numbers of obstructed vessels, decreased ischemic organ failure and kidney infarction. Preincubation of washed platelets with DNAse I inhibited platelet activation, P-selectin exposure, aggregation response to collagen, collagen-related peptide or thrombin. In addition, DNAse I-treated platelets formed less fibrin. DNAse I treatment also reduces the levels of secreted adenosine triphosphate (ATP) in human and mouse platelets, which strongly inhibits platelet aggregation, and ATP-dependent neutrophil activation (231, 288). Earlier, it was proposed that neutrophils are required for thrombosis in the laser-induced arterial injury model (289). Although DNAse I treatment induced the hydrolysis of ATP and adenosine diphosphate (ADP), decreasing fibrin formation and inhibiting thrombosis, scanning electron microscopy did not reveal classical NET structure in this thrombosis model (288).

Polyphosphate (polyP) is synthesized enzymatically from ATP and this metabolic conversion is fully reversible. PolyP is stored in dense granules of platelets, and secreted upon platelet activation. Extracellular polyP accelerates the coagulation cascade by factor V activation, promotes factor XI activation through thrombin and blocks the anti-coagulant activity of tissue factor inhibitor (TFI), thereby enhancing blood clotting (290). Interestingly, DNAse I could decrease ATP and polyP levels *in vitro* (231, 288, 291), indicating that DNAse I may inhibit ATP metabolism, or enhance ATP degradation or conversion of ATP to adenosine monophosphate (AMP). Altogether, these results suggest that the anti-thrombotic effects of DNAse I treatment in platelets may occur in an ATP/polyP-dependent manner.

Several experimental studies using cancer and thrombosis mouse models suggested that targeting extracellular DNA with DNAse I may offer a potential anti-cancer and anti-thrombotic strategy (278, 280, 292). However, only limited clinical studies with DNAse I treatment have been reported so far. In patients with cystic fibrosis, nebulized recombinant human DNAse treatment could reduce sputum viscosity and improve pulmonary function (293, 294). Therefore, recombinant human DNAse treatment is recommended in patients with cystic fibrosis and also in patients with other moderate or severe suppurative lung diseases. Further investigation is necessary whether DNAse I treatment may be effective in cancer or cancer-associated thromboinflammation.

## Other Pharmacological Approaches

### Aspirin

Aberrant arachidonic acid metabolism is involved in the inflammatory and carcinogenic processes (295). Aspirin (acetylsalicylic acid) irreversibly acetylates and thus inhibits the enzymatic activity COXs, thereby blocking the conversion of arachidonic acid to thromboxane A2 (TxA2) (296). In mouse models, aspirin treatment prevents NET-induced injury of the lung endothelium by inhibiting platelet activation and NETosis (297). A higher bacteria count in the blood was detected in aspirin-treated mice after infection, indicating that aspirin may interfere with NET functionality. However, this action of aspirin may be independent of platelet-resident COX activity, since aspirin-treated neutrophils had impaired NETosis (297, 298).

### Prostaglandin E2

Prostaglandin E2 (PGE2) is a prostanoid fatty acid metabolic product of arachidonic acid. PGE2 inhibits PMA-induced NETosis through prostanoid receptors of EP2 and EP4 (299). Studies by Domingo-Gonzalez et al., showed that murine bone marrow transplant neutrophils which overexpress COX2 induce defective bacteria clearance (300). When these neutrophils were stimulated with PMA or rapamycin, NETosis was strongly reduced compared to control. After bone marrow transfer, NET formation was rescued using COX inhibitors. The same effect was achieved *via* EP2 receptor antagonist (PF-04418948) or EP4 antagonist (AE3-208) in neutrophils from bone marrow transplant mice and hematopoietic stem cell transplant patients (300).

In mice and healthy donors, NETosis was also inhibited by exogenously injected PGE2 which was dependent on the cAMP-PKA pathway (299, 300). Consistently, incubation of neutrophils with cAMP analog dibutyryl-cAMP, rolipram or butaprost could also inhibit NETosis (299).

### Chloroquine

Chloroquine and hydroxychloroquine are anti-malarial drugs, which appeared as promising treatments also for cancer (301). Chloroquine inhibits autophagy in different cell types including neutrophils (127). Several groups have shown that autophagy promotes NETosis (62, 118, 302–304). However, studies using pharmacological inhibitors of autophagosome acidification and neutrophil- and eosinophil-autophagy‐related 5 (ATG5) conditional knock-out mice could not confirm these results (146). Chloroquine treatment reduces the severity of acute pancreatitis in mice, thereby improving survival (305). In cell culture assays, chloroquine could not diminish NETosis, indicating an indirect mechanism (306). Hydroxychloroquine is also known as an anti-inflammatory drug, which can block TLR/COX2 pathway-dependent NET formation and consequent metastasis in hepatocellular carcinoma (2, 107, 307). In the mouse model of PDAC, chloroquine treatment reversed hypercoagulability by reducing NET-mediated platelet aggregation and the release of circulating TF. Patients treated with hydroxychloroquine on a randomized protocol of preoperative chemotherapy showed a reduction in pre-operative VTE rate (127). Although several clinical trials showed the benefits of chloroquine as an anti-tumor drug (308), the precise molecular mechanisms of chloroquine-mediated effects has not been established. It was proposed that chloroquine may influence autophagy (309). Chloroquine in combination with other chemotherapeutic drugs could increase the efficiency of drug treatment, although it can accelerate chemotherapy-associated organ injury (301). Therefore, it is important to further investigate the effects of chloroquine on cancer-induced NETosis, thromboinflammation and organ injury.

### Staphylokinase

Bacterial infection of host tissues activates neutrophils and induces NET formation, thereby activating the innate immune system, including macrophage phagocytosis. Interestingly, *Staphylococcus aureus* can escape from NETs, thus converting NETs to deoxyadenosine, thereby inducing immune cell death by caspase-3-mediated mechanism. *Staphylococcus aureus* can secret nuclease and adenosine synthase which modifies the structure of NETs, thereby destroying the NET-mediated immune defense system (310). *Staphylococcus aureus* also produces a plasminogen activator staphylokinase, which is a fibrin-specific thrombolytic biomolecule (311). Staphylokinase was proposed for the therapy of stroke and myocardial infarction. However, it has a short life-time in the blood, which limits the clinical application. Strategies based on the PEGylation (attachment of polyethylene glycol) may prolong the half-life time of staphylokinase, thereby improving its bioactivity in disease conditions (312).

### Peptidyl Arginine Deiminase Inhibitors

Cl-amidine and F-amidine target all peptidyl arginine deiminase (PAD) isoforms were actively applied in many preclinical models to study NETosis. Various tumors are associated with the overexpression of PAD and increased citrullination. In 1958, Rogers and Simmonds were the first to describe protein citrullination in an animal protein as the process of converting peptidyl arginine into peptidyl citrulline. Since citrulline cannot be encoded *in vivo*, it only occurs after translation (313). Peptidyl arginine deiminases (PADs, also called PADIs) are an enzyme family which can convert protein arginine residues to citrulline in a Ca^2+^-dependent manner. This enzyme family comprises 5 isoforms (including PAD1, 2, 3, 4 and 6) which are highly conserved, have tissue-specific distribution and target substrates respectively (314).

PAD2-mediated histone citrullination is proposed as a potential therapeutic target for prostate and colon cancer (201, 315). PAD2 also regulates genes expression related to lactation through histone citrullination (316). The gonadotropin-releasing hormone (GnRH) agonist can stimulate PAD2-mediated histone H3 citrullination which epigenetically regulates the expression of gonadotropin genes such as luteinizing hormone β (LHβ) and follicle-stimulating hormone β (FSHβ) in gonadotropes (317). Recent studies have identified that PAD2 inhibition can reduce inflammatory cytokine production and NET formation in endotoxemia (318). PAD4-mediated citrullination promotes chromatin decondensation and DNA fragmentation, thereby affecting chromatin structure. PAD4 is critical for NET-mediated anti-microbial function (319). Furthermore, PAD4 can also regulate the transcriptional activity of p53 in tumor progression (320). Additionally, PAD4 promotes the metastasis of gastric tumors by regulating the expression of CXCR2, keratin K14 (KRT14) and TNFβ, which can accelerate angiogenesis, cell proliferation, migration and tumor immune microenvironment establishment (321). Inhibition of PAD4-mediated NETosis was also possible using an antagonist miR-155, which inhibits PAD4 mRNA synthesis and NET formation in response to PMA (322). In the experimental model of systemic lupus erythematosus, Cl-amidine treatment strongly inhibits NET-induced vascular damages, endothelial dysfunction and kidney injury. Inhibition of PAD4 also strongly decreased the expression of IFNγ, reduced proteinuria and immune complex attachment to the kidney tissues and in addition, protected from skin disease (323). Interestingly, PAD4-deficient mice had accelerated diabetic wound healing compared to wild-type mice (324). Although these irreversible inhibitors inactivate Ca^2+^-bound PAD4, they lack specificity and also interact with other isoforms of the PAD-family. Lewis et al., generated two reversible inhibitors GSK199 and GSK484 which are highly specific for PAD4 and can inhibit NETosis in murine and human neutrophils (325). Removal of NETs with DNAse I or pharmacological inhibition of PAD4 with GSK484 inhibitor prevent cancer-associated kidney injury in mice (326). However, in recent studies, GSK484 also enhanced irradiation-induced damages in triple-negative breast cancer cells, which subsequently had inhibitory effects on cell proliferation, migration and invasion (327). In mouse models of sepsis, deficiency of PAD4 or DNAse I treatment strongly reduced intravascular thrombin activity, inhibited platelet aggregation and improved microvascular perfusion (328). Patients with acute thrombotic microangiopathies displayed low plasma levels of DNAse I compared to the healthy subjects (329). In mouse models of HIT, genetic deficiency or GSK484-mediated inhibition of PAD4 abolishes thrombus formation (135). *In vitro*, DNAse I/GSK484 strongly inhibited the epithelial-mesenchymal transition-promoting ability of NETs in gastric cell cultures (87), indicating multiple effects of exogenous DNAse I in cancer.

Other effects may also result from PAD-mediated inhibition of ET formation. PAD1 and PAD3 target keratin K1, filaggrin and myelin, thus playing a specific role in epidermis differentiation (330). PAD enzymes are also positively associated with diffuse inflammation in the brain (331). In macrophages, PAD2 becomes activated due to increased levels of Ca^2+^ and can induce apoptosis by citrullinating vimentin (332). PAD2 citrullinates many proteins such as actin and vimentin in dendritic cells and dendritic cell-derived osteoclasts and in brain tissues (333, 334). Furthermore, overexpression of PAD2 in T cell line was shown to induce vimentin citrullination and apoptosis (335). Recently, PAD3 was found to be necessary for apoptosis-inducing factor (AIF)-mediated apoptosis in human neural stem cells (336). In comparison to other PAD family members, PAD4 has more catalytic substrates. PAD4 is involved in cell apoptosis and differentiation and deiminates nonhistone proteins such as p300, nucleophosmin (NPM1), an inhibitor of growth protein 4 (ING4) and Lamin C, which are involved in cell apoptosis or DNA damage (337). Moreover, PAD4-mediated citrullination participates in the regulation of human 40S ribosomal protein S2 (RPS2) and ribosome assembly (338). PAD4 targets collagen and decreases the adhesion of synovial fibroblasts and mesenchymal stem cells (339). DNA methyltransferase DNMT3A can be citrullinated by PAD4, which provides a novel mechanism for controlling *de novo* DNA methylation (340).

### Cyclosporine A

Cyclosporine A suppresses immunocompetent T cells reversibly and is applied for the treatment of autoimmune diseases such as rheumatoid arthritis, and further viral, fungal and parasitical infections (341). Cyclosporine A binds to cyclophilin, thereby downregulating the nuclear factor of activated T cells (NFAT) signaling, thus further inhibiting the calcineurin pathway (342). Efficient induction of NETosis requires cytoplasmic Ca^2+^ increase, linking the cyclosporine A-induced calcineurin pathway to NETosis. IL8-induced NETosis is reduced by combining treatment of ascomycin and cyclosporine A (343), suggesting a possibility to develop a therapeutic approach of NETosis.

### Heparin

Heparin is an anti-coagulant, extensively used in different therapies for the prevention of blood clotting during heart surgery, kidney dialysis, as well as for the treatment of VTE, heart attacks and angina (344). Heparin also inhibits many hallmarks of cancer, such as cancer cell survival, angiogenesis and migration (345). Moreover, heparin treatment can induce HIT, which is a life-threatening process, based on a severe immune reaction to heparin, characterized by thrombocytopenia and severe thrombosis. In patients with HIT, antibodies are produced against heparin-platelet factor 4 (PF4) complexes. Interestingly, this immune complex can directly activate neutrophils and enhance NET formation, which is sufficient for the development of thrombosis (135). PF4 binds to NETs, which renders NETs more compact. The complex then binds HIT antibodies, thereby protecting NETs from DNAse degradation (346). In the mouse model of HIT, inhibition of NET formation through PAD4 inactivation can reduce venous thrombus formation but not thrombocytopenia (346), suggesting that other alternative molecular mechanisms are involved in this process. HIT-induced NETosis is further enhanced by ROS production and NE. Interestingly, heparin derivatives, such as low molecular weight heparin, fondaparinux and heparan sulfate cannot induce profound NETosis (347). Altogether these results suggest that heparin induces neutrophil activation and NETosis contributes to venous thrombosis in HIT, which is triggered by PF4-NET-HIT antibody complexes.

The effects of unfractionated heparin, low-molecular-weight heparin (LMWH), e.g., parnaparin and non-anti-coagulant heparin were studied in histone-induced diseases. Heparin was able to protect mice and rats from organ and tissue damage, as well as death by antagonizing histones in the blood (348–351). In a mouse model of sepsis, heparin pretreatment could significantly decrease the level of NETs in serum and lung tissues (352). NET formation promotes cancer cell migration, invasion and angiogenesis, which were inhibited by heparin or other histone-binding agents (16). NETs also contributed to a variety of cancer or cancer-associated thrombosis (97). LMWH is currently the preferred treatment for prophylaxis and cancer-associated thrombosis (353). However, more experimental evidence is necessary to understand the effects of heparin in NET formation of cancer patients. Heparin derivatives may be a promising tool to cure diseases with high levels of plasma histones, thereby potentially inhibiting NETosis without dramatic changes in hemostasis.

### Metformin

Metformin was originally used in diabetic patients to normalize blood glucose levels. The anti-diabetic effects of metformin are due to the inhibition of hepatic gluconeogenesis, which is possibly associated with an insulin-mediated increase in glucose uptake in skeletal muscle cells (354). Metformin acts by inducing adenosine monophosphate-activated kinase (AMPK), an enzyme regulating energy metabolism through activation of glucose or oxidation of fatty acids (355). High glucose and hyperglycemia increase the release of NETs and circulating markers of NETosis, respectively (356). Although metformin inhibited NETosis *in vitro* by reducing proteinase-3, histones and extracellular DNA, it did not affect insulin synthesis. In neutrophils, metformin prevented membrane translocation of PKCβII and activation of Nox, thereby decreasing NETosis in response to PMA and Ca^2+^. In line with this, metformin also decreased NET components in the plasma of patients with type 2 diabetes before and after treatment with insulin or dapagliflozin (357).

Circulating neutrophil levels are often increased in patients with a polycystic ovarian syndrome which is associated with an increased risk to develop ovarian cancer (358–360). Ibanez et al., reported that metformin can reduce neutrophil count in polycystic ovarian disease (360). These studies support the idea that metformin can prevent the increased neutrophil levels and NETosis that are associated with aggressive forms of ovarian cancer.

NET-independent mechanisms of metformin have also been observed. Metformin could impair tumor growth when administered during fasting-induced hypoglycemia. The anti-tumor effects of metformin were mediated by glycogen synthase kinase 3β (GSK3β) activation and PP2A-B56δ complex formation (361). Metformin inhibited the growth of a variety of breast cancer cells by inducing cell cycle arrest and apoptosis (362). Similar to other cell lines, metformin also induces AMPK activation, reduced the phosphorylation of epidermal growth factor receptor (EGFR), mitogen activated protein kinases (MAPKs) and Src and lowered the levels of cyclins D1 and E in breast cancer cells (362). Metformin also inhibits signal transducer and activator of transcription 3 (STAT3) activation and thereby reduced cell proliferation (363). Furthermore, metformin also activates p53 by activating AMPK, ultimately stopping the cell cycle (357, 364).

Metformin inhibits the proliferation of breast cancer cells with aberrant expression of human epidermal growth factor receptor 2 (HER2). Translational suppression of HER2 expression was observed after metformin treatment and this effect was triggered by the inhibition of the mTOR-S6K1 signaling pathway (365). Besides breast cancer, metformin can inhibit the proliferation of prostate, endometrial and brain cancer cells. Similar effects induced by metformin, inducing cell cycle arrest and suppressing the mTOR signaling pathway (366–368). *In vivo* experimental conditions, metformin treatment significantly reduced the primary tumor size of mammary adenocarcinomas and prolonged the lifespan of MMTV–Her2/Neu mice (369). Heterozygote mice of the tumor suppressor gene PTEN develop tumors in different organs, and metformin delayed tumor onset by 25% (370).

Many inhibitory effects of metformin on tumor growth through AMPK and mTOR signalings were confirmed using different mouse models of cancer (371–375). Metformin was also effective in reducing the growth of intestinal polyps in tumor suppressor Apc-mutant mice (376) by reducing mTOR/S6K/S6 signaling in the epithelium of the intestine. Of note, in this intestinal tumorigenesis model, tumor growth was shown to be associated with increased neutrophil infiltration and NETosis (130), raising the possibility that metformin may also inhibit NET-dependent tumor growth.

Although numerous preclinical, clinical and epidemiological studies proposed that metformin treatment inhibits tumor growth compared to other hypoglycemic treatments, it is still an open question whether metformin can be a potential candidate for the treatments of cancers predisposing tumor microenvironment to the release of ETs.

### Thrombomodulin/Activated Protein C Complex

Thrombomodulin is an endothelial receptor, playing an important role in vascular homeostasis and regulation of coagulation. Thrombomodulin forms a complex with thrombin, thereby inactivating the coagulant activity of thrombin which activates protein C and thrombin activatable fibrinolysis inhibitor (TAFI) (377). Besides thrombin, thrombomodulin also regulates the inactivation of complement 3b (378). Recombinant thrombomodulin is given to the patients with disseminated intravascular coagulation, thereby protecting them from tissue injury (379). Recombinant thrombomodulin neutralizes damage-associated molecular patterns (DAMPs), including histones and HMGB1, inhibits aberrant activation of the complement system, protecting the endothelium (380). Using *in vitro* platelet-neutrophil coculture models, Shimomura et al., demonstrated that recombinant thrombomodulin may inhibit LPS-induced NETosis (381). Later, Helms et al. found that treatment of rats with recombinant thrombomodulin during septic shock limits excessive neutrophil activation and rescues a balanced coagulation and immunothrombosis response (382). This promising therapeutic tool would be important to follow in the future using mouse models of cancer.

Activated protein C (APC) is a serine protease with anti-coagulant and anti-inflammatory effector functions. Activation of the blood coagulation cascade by TF induces thrombin generation and the formation of a fibrin network. In addition, thrombin binds to thrombomodulin, and activates protein C in complex with endothelial protein C receptor (EPCR). Zymogen protein C is cleaved by thrombin to generate functionally active APC. After protein cleavage, APC forms a complex with protein S, and inactivates coagulation factors (Va, VIIIa) and as a negative feedback loop, inhibits thrombin generation (383). Therefore, long-term APC treatment could potentially increase the risk of bleeding complications (384). Besides this function, APC binds and activates PAR1 thereby enhancing vascular barrier integrity through sphingosine-1-phosphate receptor 1 (S1P1)-VE-cadherin signaling (385, 386).

The anti-inflammatory effects of APC involve the inhibition of neutrophil activation, NET formation and cell death. APC can effectively inhibit PI3K-PKC-dependent NET formation and this process is strongly dependent on the functional crosstalk between the macrophage-1 antigen (Mac-1), EPCR, and protease-activated receptor 3 (PAR3). APC can cleave PAR3 at a different site than thrombin, thereby inhibiting NET formation. Consequently, antibodies of EPCR, PAR3 and Mac-1 can reverse APC-mediated inhibition of NETosis (387).

Due to the multiple roles of APC in hemostasis and inflammation, it is difficult to predict the positive or negative effects of APC treatment in cancer progression. Increased levels of APC in the blood may limit metastasis by protecting the vascular barrier through VE-cadherin, but it may stimulate the metastatic potential of cancer cells (386). It has been shown that APC signaling enhances cancer cell migration, invasion and angiogenesis and also inhibits apoptosis (386, 388, 389). APC treatment could enhance breast cancer cell invasion in a dose-dependent manner (389). Cancer patients require long-term APC treatment which may induce severe bleeding complications (386), due to the hemostatic effects of APC on thrombin generation and factor Va/VIIIa functions. Although APC has a strong anti-inflammatory potential for the treatment of human patients, it is necessary to test recombinant mutant forms of APC with selective anti-inflammatory function in experimental models of cancer, without affecting thrombin generation and hemostasis.

### Diphenyleneiodonium Chloride

Diphenyleneiodonium chloride (DPIC) is a hypoglycemic agent, identified as an inhibitor of NADH/Nox with highly potent anti-microbial activity against *Mycobacterium tuberculosis* and *Staphylococcus aureus* (390, 391). However, DPIC can also inhibit nitric oxide synthase (392), xanthine oxidase (393) and NADPH cytochrome P450 oxidoreductase (394), thereby inhibiting ROS production. Furthermore, DPIC inhibits oxidative phosphorylation (OXPHOS) and consequently reduces ATP production, thereby switching energy production to the lactic acid energy system (390). DPIC treatment strongly inhibits mitochondria function, thus leading to metabolic senescence (395). Interestingly, the effects of short-term DPIC treatment on cancer cells were independent of p53. However, long-term treatment showed that p53 expression facilitates a prolonged cell cycle arrest and protects cancer cells from apoptosis, while p53 deficiency could induce apoptosis with poly ADP-ribose polymerase (PARP) cleavage and DNA fragmentation in cancer cells (396). Altogether, these results suggest that DPIC treatment can reduces tumor growth by the inhibition of cancer cell proliferation and activation of the immune system through factors secreted by senescent cancer cells.

DPIC inhibits extracellular DNA release in PMA-stimulated neutrophils, although the degree of DPIC-inhibited NET formation was strongly dependent on the dose of external stimuli (49, 80). In different lung epithelial cells, NET formation is significantly increased by the secretion of CXCL8, IL8 and IL6 and this process was inhibited by DPIC (397). In the model of *in vitro* cigarette smoke extract-induced NETosis, DPIC treatment also inhibited this process (398). In mice, tobacco smoke increases lung metastasis by sustaining lung inflammation and thereby inducing NETosis which subsequently awake dormant cancer cells (108). Further investigation is necessary to show the effects of DPIC in long-term treated tumors and tumor microenvironment *in vivo*, focusing on the context of mitochondria dysfunction and senescence, as well as the distribution of NETs in cancer and lung metastasis.

### High Mobility Group box-1 Antagonists

High mobility group box-1 (HMGB1) is a nonhistone chromatin-associated protein, and as a nuclear cofactor in transcription regulation, interacting with many transcription factors and histones, supporting gene expression in the cells (399). However, HMGB1 is also secreted into the extracellular milieu, thereby initiating several interactions with receptors on the cell surface or with extruded DNA, triggering various signaling mechanisms and NETosis (399, 400). HMGB1 has a cytokine-like activity, thus regulating immune cell functions, including chemotaxis and immune modulation (401). In monocyte/macrophage-infiltrating disease conditions, HMGB1 facilitates macrophage reprogramming towards a proinflammatory phenotype through TLR4 activation (402). Interestingly, exposure to HMGB1 strongly increases the amount of extracellular DNA and citrullinated histone 3 in wild-type neutrophils, however, this effect was not observed in TLR4-deficient neutrophils (403). In mouse models treated with LPS, HMGB1 antibody treatment could decrease the levels of citrullination of histone 3 (403). Altogether, these results suggest that HMGB1 is a potential target for the development of anti-inflammatory therapies against TLR4-mediated NETosis.

Interestingly, metformin as a potential inhibitor of NETosis directly binds to the C-terminal tail of HMGB1 (357, 404). In the acute liver damage model, HMGB1 was released from damaged liver cells and metformin treatment could inhibit this process, protecting the liver cells (404). In another mouse model, metformin can significantly inhibit HMGB1 secretion and consequently reduce LPS-induced macrophage inflammatory responses, thereby improving the survival of endotoxemic mice (405). Altogether, these results suggest that metformin would be a potential drug to inhibit HMGB1-induced inflammation and NETosis.

Platelets are the major reservoirs of HMGB1, and it is released by activated platelets (400). HMGB1 binds TLR4 receptors on the platelet surface, thereby inducing recruitment of myeloid differentiation primary response 88 (Myd88) and guanylyl cyclase to the plasma membrane, leading to the activation of cGMP-dependent protein kinase I (400, 406). In a mouse model lacking HMGB1 in platelets, decreased thrombosis, lung inflammation and NETosis were observed indicating pleiotropic effects of HMGB1 in thromboinflammation (406).

Cancer cell-derived HMGB1 can modulate platelet-resident TLR4 receptors, thereby increasing platelet-dependent tumor metastasis. Although NETosis was not addressed in this study, blocking HMGB1 function in tumor cells was effective to inhibit tumor metastasis (407). HMGB1 is also expressed in keratinocytes. HMGB1-deficient keratinocytes displayed a marked reduction in NET formation, and subsequently delaying wound healing and promoting tumorigenesis in mice (408). Using anti-HMGB1 antibody treatment, HMGB1-mediated NETosis was strongly inhibited (403). It is tempting to investigate whether HGMB1 may also trigger DNA release from other inflammatory immune cells in proinflammatory tumor microenvironment.

### Purinergic P2Y12 Receptor Blockers

Purinergic P2Y12 receptor (P2Y12) blockers (clopidogrel, ticagrelor, cangrelor, prasugrel) are widely used in patients with cerebrovascular, coronary artery, cerebrovascular and peripheral vascular diseases (409). The thienopyridine-derived metabolite irreversibly inhibits the binding of ADP to the receptor P2Y12, resulting in decreased platelet activation and aggregation responses, and reducing inside-out activation of platelet integrin αIIbβ3 integrin (410). Neutrophil-mediated platelet activation was suggested to be dependent on ADP (411, 412), therefore ADP blockers consequently attenuate platelet-neutrophil interactions and NETosis. In a mouse model of cholesterol crystal embolism, extracellular DNA has been exposed from NETs and damaged endothelium and activated platelets from emboli and vascular occlusion, leading to tissue infarction and kidney injury (231). In this model, P2Y12 blockade similarly to the DNAse I treatment strongly inhibited platelet function, consequently inhibiting extracellular DNA release and associated ischemia and organ injury (231).

In ST-elevation myocardial infarction (STEMI), the interaction between platelets and neutrophils results in the secretion of polyP in the presence of thrombin (413). In the infarct-related arteries, platelets release polyP, stimulating neutrophils to form thrombogenic/TF-bearing NETs (414, 415). Although ticagrelor significantly inhibited the NETotic effect of coronary stents *in vitro*, this did not rely on the P2Y12 receptor. These results indicated that ticagrelor may have pleiotropic effects on NETosis independently of platelets (415). The pancreatic cancer microenvironment is highly rich in tumor-associated neutrophils, platelets and NETs (416). Clopidogrel was shown to inhibit cancer growth and metastasis in PANC02 pancreatic cancer model (417). In the future, it will be important to analyse the effect of P2Y12 blockers on cancer-associated neutrophil activation and NETosis.

### Disulfiram

Disulfiram inhibits aldehyde dehydrogenase and is used to treat alcohol dependence (418). Disulfiram is also a potent inhibitor of gasdermin D in mouse and human macrophages and neutrophils (419, 420). Gasdermin D is a pore-forming protein playing a pivotal role in inflammatory cell death (419). In macrophages, inflammasome activation by canonical and or non-canonical pathways induces the cleavage of gasdermin D, which translocates to the plasma membrane thereby forming pores and inducing pyroptosis (421). In neutrophils, cytoplasmic caspase was shown to be directly activated by LPS or gram-negative bacteria independently of TLR4 (422). A recent study by Silva et al., showed that during sepsis caspase-11 activation induces gasdermin D cleavage, resulting in NET formation (420). Besides these mechanisms, gasdermin D cleavage is also generated by NE, which is released from neutrophils upon activation (423). Interestingly, inhibition of gasdermin D with disulfiram abolished NET formation reducing multiple organ dysfunction and sepsis-associated lethality (420). These studies indicate that disulfiram could be an important therapeutic agent to target gasdermin D, thereby preventing organ injury.

### Diethylcarbamazine

Diethylcarbamazine (DEC) is a derivate of piperazine, used as an anti-parasitic drug (424). Although at low doses DEC improves cytokine production, a high dose of this drug increases the respiratory burst in neutrophils (425). *In vivo*, DEC reduces the inflammatory granuloma formation in a bacterial infection model (426). DEC also decreases NET formation of neutrophils isolated from healthy subjects upon *in vitro* activation with PMA (427). In a follow-up study, DEC in both healthy donors and diabetes mellitus type 2 patients displayed an immunomodulatory effect inhibiting and delaying the tendency toward NET formation by their neutrophils (428). DEC in addition to inhibiting NETosis, also inhibits COX2, NF-kB activation, iNOS, TNFα and IL1β (429), indicating that the effects of DEC can be associated with many immunomodulatory pathways.

### Glucuronoxylomannan

Glucuronoxylomannan (GXM), a polysaccharide, represents the main capsular content of the opportunistic yeast *Cryptococcus neoformans*, which has potent immunosuppressive properties. In a mouse model of rheumatoid arthritis triggered by collagen type II, GXM could improve the disease severity, by downregulating the cytokine and growth factor (TNFα, IL1β, IL6 and TGFβ) levels, thereby inhibiting Th17 cell differentiation and subsequent IL17 secretion (430). Furthermore, Rocha et al., showed that GXM treatment could abolish NET formation, independently of the agonist and stimuli (431). Future studies are required to validate whether GXM could be a potential therapeutic tool in ET-mediated thromboinflammation and cancer triggered by various cell types.

### Anti-Citrullinated Antibodies

Anti-citrullinated protein antibodies (ACPAs), produced against citrullinated proteins, are diagnostic and prognostic markers of rheumatoid arthritis (432). Recent studies also provided evidence for circulating autoantibodies against citrullinated tumor-associated proteins in breast cancer patients (433). Anti-citrullinated proteins specifically targeting citrulline at histone 2A and 4 positions were proposed as a direct approach to inhibit murine and human NET formation (434).

## Conclusion

Research studies during the last decade provided important progress on better understanding of the pathophysiological role of ETs (**Table 1**). Neutrophils release ETs in response to proinflammatory stimuli and tumor cell and tumor microenvironment. Cancer-mediated NETosis also induce thrombosis, which leads to multiple organ failure. Dissolution of NET structures by DNAse I may represent benefits, but side-effects of such treatment may also result in secondary immune responses and procoagulant environment triggered by disseminated NET fragments circulating in the body. Increasing experimental and clinical evidence indicates the multiple sources of ETs in different pathological contexts, such as intestinal inflammation, sepsis, thrombosis, autoimmune diseases and diabetes. The proinflammatory and proangiogenic landscape of tumor microenvironment can potentially trigger activatory signaling pathways of ET formation, in different immune cells, including eosinophils, dendritic cells, monocytes, macrophages, basophils and lymphocytes. So far, only limited experimental and clinical evidence is available to link non-neutrophil ETs to the cancer progression and response to the anti-cancer therapies. Therefore, studies evaluating localization of extracellular DNA and traps, including immunohistological detection of colocalized cell-lineage-derived proteins, citrullinated histones, detection of extracellular DNA and traps in serum and blood samples using flow cytometry are of paramount importance. The analysis of citrullinome signature associated with immune response and response to anti-cancer treatments may offer potential diagnostic and prognostic approaches. Understanding the underlying mechanisms of ETosis in cancer and grasping the impact of nucleases, anti-thrombotic, anti-diabetic, anti-malaria and immunosuppressive drugs on ETs may help to interconnect treatment strategies between several disease contexts and propose new therapeutic modalities for the prevention and treatment of cancer.

**Table 1:** Pathophysiological role of ETs in cancer.

Biological effect	ET type	Cancer model	Underlying mechanism	Ref.
**Tumor growth**	NETs	Colorectal cancer *In vitro:* DKs-8, DKO-1 cells *In vivo:* Apc-KRAS^G12D^ mouse model	Cancer cells transfer KRAS mutations through exosomes to neutrophils and induce neutrophil recruitment and NETosis via upregulation of IL8, promoting cancer cell proliferation.	(96)
		Colorectal cancer *In vitro:* MC38 cells *In vivo:* syngeneic subcutaneous MC38 cancer model	NET-associated PD-L1 induces T cell exhaustion and enhances tumor growth.	(109)
		Hepatocellular carcinoma *In vivo:* DEN-HFCD, STAM mouse models	NETs enhance differentiation of regulatory T cells by promoting mitochondrial oxidative phosphorylation in naive CD4+ T cells via TLR4, amplifying tumor burden.	(110)
**Migration, Invasion;** **EMT**	NETs	Breast cancer *In vitro:* MCF7 cells	NETs enhance the expression of EMT markers ZEB1, Snail and fibronectin, cancer stem cell marker CD44, proinflammatory mediators, such as IL1β, IL6, IL8, CXCR1, MMP2 and MMP9.	(86)
		Gastric cancer *In vitro:* AGS cells	NETs enhance cancer cell migration and induce EMT; downregulation of E-cadherin and upregulation of vimentin expression.	(87)
		Pancreatic cancer *In vitro:* BxPC3, MIA, PaCa2, PANC1 cells *In vivo:* subcutaneous MIA and PaCa2 xenograft cancer models *Ex vivo:* human PDAC	Release of IL1β during NETosis activates EGFR/ERK pathway, leading to the EMT; downregulation of E-cadherin and upregulation of Snail, N-cadherin and vimentin expression.	(88)
		Colorectal cancer *In vitro:* DKs-8, DKO-1 cells *In vivo:* Apc-KRAS^G12D^ mouse model	KRAS mutant exosomes from tumor cells induce NETosis via IL8, leading to the enhanced cancer cell migration and invasion.	(96)
		Breast cancer *In vitro:* 4T1, 4T07, BT-549 and C3(1)-Tag cells	Cancer cell-derived G-CSF primes neutrophils, resulting in lytic NETosis; cathepsin G enhances NET-mediated cancer cell invasion among other NET-associated proteins.	(14)
		Pancreatic cancer *In vitro:* AsPC-1 cells	NETs induce cancer cell migration via TLR2 and TLR4.	(16)
	METs	Colon cancer *In vitro:* HCT116 and SW480 cells *Ex vivo:* human colon cancer	Cancer cells promote MET formation via PAD2; METs interact with tumor cells and enhance tumor cell invasion.	(201)
**Metastasis**	NETs	Breast cancer *In vitro:* 4T1 series, AT3, MDA-MB-231 and sublines *In vivo:* syngeneic orthotopic (4T1 series, AT3), xenograft (MDA-MB-231 and sublines) cancer models *Ex vivo:* human breast cancer	Tumor-derived cathepsin C (CTSC) triggers CTSC-PR3-IL1β axis in neutrophils, upregulating IL6 and CCL3 synthesis. CTSC-PR3-IL1β induces ROS production and NET formation which degrade thrombospondin-1, thereby supporting metastatic growth of lung cancer cells.	(95)
		Breast cancer *In vivo:* 4T1 experimental and spontaneous breast cancer metastasis models	NETs enhance lung metastasis.	(14)
		Breast cancer and colon cancer *In vitro:* MDA-MB-231, MCF-7 and HCT116 cells *In vivo:* syngeneic (4T1) and xenograft (MDA-MB-231) orthotopic and intrasplenic (MMTV-PyMT mice and E0771 cells) cancer models *Ex vivo:* human breast and colon cancer	CCDC25 on cancer cell surface acts as a sensor and binding partner for NET-DNA; binding leads to activation of ILK–β-parvin–RAC1–CDC42 cascade, cytoskeleton remodeling and formation of distant metastases.	(18)
		Breast cancer *In vitro:* D2.0R, MCF7 cells *In vivo:* syngeneic (D2.0R) and xenograft (MCF7) experimental breast cancer metastasis models	NET-associated NE and MMP9 cleave laminin and degrade thrombospondin-1 leading to the activation of integrin α3β1 and FAK/ERK/MLCK/YAP signaling, resulting in reactivation of dormant cancer cells during tumor metastasis.	(108)
		Colon, melanoma, lung and breast cancer *In vitro:* primary melanoma and LS174T, HT29 cells *In vivo:* syngeneic subcutaneous (4T1, LLC and HT29) and intradermic (B16OVA and 4T1) cancer models	Cancer cells trigger NETosis by CXCR1 and CXCR2 activation; NETs protect tumor cells from contact with cytotoxic T cells and NK cells, promoting cancer cell dissemination and lung metastasis.	(105)
		Lung cancer *In vitro:* A549 cells *In vivo:* experimental liver metastasis of A549 cells (intrasplenic injection into caecal ligation and puncture-induced sepsis model)	Tumor- and NET-derived β1-integrin mediates adhesion of NETs to circulating tumor cells, facilitating cancer cell adhesion to the liver sinusoids.	(102)
		Ovarian cancer *In vitro:* ES2 and ID8 cells *In vivo*: syngeneic (ID8) and xenograft (ES2), (intrabursal and intraperitoneal injection) cancer models	Cancer-derived cytokines (IL8, G-CSF, GROα, GROβ) promote NETosis; NETs accumulate in premetastatic niche and enhance the formation of omental metastases.	(20)
	METs	Colon cancer *In vivo:* MC38 experimental colon cancer metastasis model *Ex vivo:* human colon cancer	Cancer cells promote MET formation via PAD2, enhancing the formation of liver metastases.	(201)
**Cancer-associated thrombosis**	NETs	Chronic myelogenous leukemia (CML), breast and colon cancer *In vivo*: syngeneic orthotopic breast (4T1) and subcutaneous lung (LLC) and CML mouse models	Cancer cells predispose neutrophils to form NETs via G-CSF, promoting microthrombosis in the lung.	(97)
		Breast cancer *In vivo:* syngeneic orthotopic breast (4T1 and 67NR) models	Cancer-derived G-CSF induces neutrophilia and NETosis, leading to the prothrombotic phenotype.	(113)
		Glioma *Ex vivo:* human glioma	Platelets of late-stage glioma patients induce NETosis via P-Selectin and NETs promote hypercoagulant state and thrombogenicity in endothelial cells.	(125)
		Myeloproliferative neoplasms (MPN) *In vivo:* Jak2^V617F^ mouse model *Ex vivo:* human MPN	*Jak2* ^V617F^ mutation stimulates NET formation and thrombosis in a PAD4-dependent manner.	(132)
		Pancreatic cancer *In vitro:* AsPC-1 cells *Ex vivo:* pancreatic and biliary cancer	Tumor cells induce NET generation in a cAMP- and thrombin-dependent, and ROS-independent manner; NETs enhance thrombin generation.	(16)
		Pancreatic cancer *Ex vivo:* orthotopic (Panc02) cancer model, human pancreatic cancer	NETs induce RAGE-dependent platelet aggregation and increase TF expression, thereby enhancing coagulation.	(127)
		Pancreatic cancer *In vitro:* AsPC-1 cells	Platelets primed by tumor cells induce rapid NET generation; NETs trap platelets and stimulate thrombus formation under shear conditions.	(128)
		Small intestine cancer *In vivo* and *ex vivo:* Apc^Min/+^ mouse model	Inflammation-associated complement activation via neutrophil C3aR induces NETosis, hypercoagulation, and N2 neutrophil polarization in small intestine.	(130)
		*Ex vivo:* human solid cancers Prostate, liver, lung, bladder and breast	Malignant tumors enhance NETosis via G-CSF, inducing microthrombosis and the occurrence of ischemic stroke with elevated troponin levels.	(134)
**Secondary organ damage**	NETs	Breast cancer and insulinoma *In vivo:* MMTV-PyMT and RIP1-Tag2 transgenic models	Cancer cell-derived G-CSF induces systemic NETosis. NETs occlude kidney and heart vessels, inducing irregular blood flow, increased endothelial cell activation with upregulated expression of proinflammatory mediators, ICAM1, VCAM1, E-selectin, IL1β, IL6, and CXCL1.	(98)
**Poor prognosis and therapeutic resistance**	NETs	Bladder cancer *In vitro:* MB49, UM-UC3 cells *In vivo:* syngeneic heterotopic MB49 bladder cancer model *Ex vivo:* human bladder tumor	Radiation induces HMGB1 release in tumor microenvironment, triggering NETosis through TLR4; NETs enhance resistance to radiotherapy by suppressing CD8+ T cell infiltration.	(111)
	NETs, METs	*Ex vivo:* human pancreatic neuroendocrine tumors	Poor prognosis and postoperative recurrence of resected tumors.	(200)
	NETs,EETs	*Ex vivo:* human classic Hodgkin lymphoma, nodular sclerosis subtype	Eosinophilia and detection of NETs and EETs in lymph tumor tissues. Correlation between NET formation and fibrosis High expression of PAR-2 and nuclear p-ERK in cancer cells. Enhanced TF expression and procoagulancy in tumor-associated endothelium.	(160)

EMT, epithelial-mesenchymal transition; DEN-HFCD, diethylnitrosamine + choline-deficient, high-fat diet; STAM, Stelic Animal Model; MMTV-PyMT, mouse mammary tumor virus-polyoma middle tumor-antigen.

## Author contributions

MM and EM-B wrote the manuscript. AP, CH and AB contributed to the writing and drafted the figures. H-JA and TG critically reviewed the manuscript and contributed to the writing. All authors contributed to the article and approved the submitted version.

## Funding

This work was supported by the Bayerisches Landesamt für Gesundheit und Lebensmittelsicherheit (BLGL), project number 15-25, Deutsche Forschungsgemeinschaft, CRC TRR152/P15, AN372/14-4 and AN372/30-1 and Förderprogramm für Forschung und Lehre (FöFoLe), LMU, Munich, Germany. MM, AP and CH were recipients of a fellowship from FöFoLe, 2020-2022, BLGL and CSC (China Scholarship Council), respectively.

## Conflict of Interest

The authors declare that the research was conducted in the absence of any commercial or financial relationships that could be construed as a potential conflict of interest.

## Publisher’s Note

All claims expressed in this article are solely those of the authors and do not necessarily represent those of their affiliated organizations, or those of the publisher, the editors and the reviewers. Any product that may be evaluated in this article, or claim that may be made by its manufacturer, is not guaranteed or endorsed by the publisher.

## References

[1] Mandel P Metais P. Nuclear Acids In Human Blood Plasma. C R Seances Soc Biol Fil (1948) 142(3-4):241–3. 18875018

[2] Thierry AR El Messaoudi S Gahan PB Anker P Stroun M. Origins, Structures, and Functions of Circulating DNA in Oncology. Cancer Metastasis Rev (2016) 35(3):347–76. doi: 10.1007/s10555-016-9629-x PMC5035665 27392603

[3] Leon SA Shapiro B Sklaroff DM Yaros MJ. Free DNA in the Serum of Cancer Patients and the Effect of Therapy. Cancer Res (1977) 37(3):646–50. 837366

[4] Stroun M Anker P Lyautey J Lederrey C Maurice PA. Isolation and Characterization of DNA From the Plasma of Cancer Patients. Eur J Cancer Clin Oncol (1987) 23(6):707–12. doi: 10.1016/0277-5379(87)90266-5 3653190

[5] Bettegowda C Sausen M Leary RJ Kinde I Wang Y Agrawal N. Detection of Circulating Tumor DNA in Early- and Late-Stage Human Malignancies. Sci Transl Med (2014) 6(224):224ra24. doi: 10.1158/1538-7445.AM2014-5606 PMC4017867 24553385

[6] Haber DA Velculescu VE. Blood-Based Analyses of Cancer: Circulating Tumor Cells and Circulating Tumor DNA. Cancer Discov (2014) 4(6):650–61. doi: 10.1158/2159-8290.CD-13-1014 PMC4433544 24801577

[7] Fernandez-Mercado M Manterola L Larrea E Goicoechea I Arestin M Armesto M. The Circulating Transcriptome as a Source of non-Invasive Cancer Biomarkers: Concepts and Controversies of non-Coding and Coding RNA in Body Fluids. J Cell Mol Med (2015) 19(10):2307–23. doi: 10.1111/jcmm.12625 PMC4594673 26119132

[8] Zhou B Xu K Zheng X Chen T Wang J Song Y. Application of Exosomes as Liquid Biopsy in Clinical Diagnosis. Signal Transduct Target Ther (2020) 5(1):144. doi: 10.1038/s41392-020-00258-9 32747657 PMC7400738

[9] Aucamp J Bronkhorst AJ Badenhorst CPS Pretorius PJ. The Diverse Origins of Circulating Cell-Free DNA in the Human Body: A Critical Re-Evaluation of the Literature. Biol Rev Camb Philos Soc (2018) 93(3):1649–83. doi: 10.1111/brv.12413 29654714

[10] Jahr S Hentze H Englisch S Hardt D Fackelmayer FO Hesch RD. DNA Fragments in the Blood Plasma of Cancer Patients: Quantitations and Evidence for Their Origin From Apoptotic and Necrotic Cells. Cancer Res (2001) 61(4):1659–65. 11245480

[11] Schwarzenbach H Hoon DS Pantel K. Cell-Free Nucleic Acids as Biomarkers in Cancer Patients. Nat Rev Cancer (2011) 11(6):426–37. doi: 10.1038/nrc3066 21562580

[12] Brinkmann V Reichard U Goosmann C Fauler B Uhlemann Y Weiss DS. Neutrophil Extracellular Traps Kill Bacteria. Science (2004) 303(5663):1532–5. doi: 10.1126/science.1092385 15001782

[13] Papayannopoulos V. Neutrophil Extracellular Traps in Immunity and Disease. Nat Rev Immunol (2018) 18(2):134–47. doi: 10.1038/nri.2017.105 28990587

[14] Park J Wysocki RW Amoozgar Z Maiorino L Fein MR Jorns J. Cancer Cells Induce Metastasis-Supporting Neutrophil Extracellular DNA Traps. Sci Transl Med (2016) 8(361):361ra138. doi: 10.1126/scitranslmed.aag1711 PMC5550900 27798263

[15] Seo JD Gu JY Jung HS Kim YJ Kim HK. Contact System Activation and Neutrophil Extracellular Trap Markers: Risk Factors for Portal Vein Thrombosis in Patients With Hepatocellular Carcinoma. Clin Appl Thromb Hemost (2019) 25:1076029618825310. doi: 10.1177/1076029618825310 30808222 PMC6715110

[16] Jung HS Gu J Kim JE Nam Y Song JW Kim HK. Cancer Cell-Induced Neutrophil Extracellular Traps Promote Both Hypercoagulability and Cancer Progression. PloS One (2019) 14(4):e0216055. doi: 10.1371/journal.pone.0216055 31034495 PMC6488070

[17] Li Y Yang Y Gan T Zhou J Hu F Hao N. Extracellular RNAs From Lung Cancer Cells Activate Epithelial Cells and Induce Neutrophil Extracellular Traps. Int J Oncol (2019) 55(1):69–80. doi: 10.3892/ijo.2019.4808 31115506 PMC6561626

[18] Yang L Liu Q Zhang X Liu X Zhou B Chen J. DNA of Neutrophil Extracellular Traps Promotes Cancer Metastasis *via* CCDC25. Nature (2020) 583(7814):133–8. doi: 10.1038/s41586-020-2394-6 32528174

[19] Cools-Lartigue J Spicer J Najmeh S Ferri L. Neutrophil Extracellular Traps in Cancer Progression. Cell Mol Life Sci (2014) 71(21):4179–94. doi: 10.1007/s00018-014-1683-3 PMC7096049 25070012

[20] Lee W Ko SY Mohamed MS Kenny HA Lengyel E Naora H. Neutrophils Facilitate Ovarian Cancer Premetastatic Niche Formation in the Omentum. J Exp Med (2019) 216(1):176–94. doi: 10.1084/jem.20181170 PMC6314534 30567719

[21] Daniel C Leppkes M Munoz LE Schley G Schett G Herrmann M. Extracellular DNA Traps in Inflammation, Injury and Healing. Nat Rev Nephrol (2019) 15(9):559–75. doi: 10.1038/s41581-019-0163-2 31213698

[22] Conceicao-Silva F Reis CSM De Luca PM Leite-Silva J Santiago MA Morrot A. The Immune System Throws Its Traps: Cells and Their Extracellular Traps in Disease and Protection. Cells (2021) 10(8):1891. doi: 10.3390/cells10081891 34440659 PMC8391883

[23] Mortaz E Alipoor SD Adcock IM Mumby S Koenderman L. Update on Neutrophil Function in Severe Inflammation. Front Immunol (2018) 9:2171. doi: 10.3389/fimmu.2018.02171 30356867 PMC6190891

[24] Borregaard N. Neutrophils, From Marrow to Microbes. Immunity (2010) 33(5):657–70. doi: 10.1016/j.immuni.2010.11.011 21094463

[25] Haus E Smolensky MH. Biologic Rhythms in the Immune System. Chronobiol Int (1999) 16(5):581–622. doi: 10.3109/07420529908998730 10513884

[26] Nathan C. Neutrophils and Immunity: Challenges and Opportunities. Nat Rev Immunol (2006) 6(3):173–82. doi: 10.1038/nri1785 16498448

[27] Rabinovitch M. Professional and non-Professional Phagocytes: An Introduction. Trends Cell Biol (1995) 5(3):85–7. doi: 10.1016/S0962-8924(00)88955-2 14732160

[28] Cowland JB Borregaard N. Granulopoiesis and Granules of Human Neutrophils. Immunol Rev (2016) 273(1):11–28. doi: 10.1111/imr.12440 27558325

[29] Paoliello-Paschoalato AB Marchi LF de Andrade MF Kabeya LM Donadi EA Lucisano-Valim YM. Fcgamma and Complement Receptors and Complement Proteins in Neutrophil Activation in Rheumatoid Arthritis: Contribution to Pathogenesis and Progression and Modulation by Natural Products. Evid Based Complement Alternat Med (2015) 2015:429878. doi: 10.1155/2015/429878 26346244 PMC4540990

[30] Futosi K Fodor S Mocsai A. Neutrophil Cell Surface Receptors and Their Intracellular Signal Transduction Pathways. Int Immunopharmacol (2013) 17(3):638–50. doi: 10.1016/j.intimp.2013.06.034 PMC3827506 23994464

[31] Campbell MS Lovell MA Gorbsky GJ. Stability of Nuclear Segments in Human Neutrophils and Evidence Against a Role for Microfilaments or Microtubules in Their Genesis During Differentiation of HL60 Myelocytes. J Leukoc Biol (1995) 58(6):659–66. doi: 10.1002/jlb.58.6.659 7499963

[32] Manley HR Keightley MC Lieschke GJ. The Neutrophil Nucleus: An Important Influence on Neutrophil Migration and Function. Front Immunol (2018) 9:2867. doi: 10.3389/fimmu.2018.02867 30564248 PMC6288403

[33] Borregaard N Cowland JB. Granules of the Human Neutrophilic Polymorphonuclear Leukocyte. Blood (1997) 89(10):3503–21. doi: 10.1182/blood.V89.10.3503.3503_3503_3521 9160655

[34] Spitznagel JK Dalldorf FG Leffell MS Folds JD Welsh IR Cooney MH. Character of Azurophil and Specific Granules Purified From Human Polymorphonuclear Leukocytes. Lab Invest (1974) 30(6):774–85. 4134220

[35] Evans TJ Buttery LD Carpenter A Springall DR Polak JM Cohen J. Cytokine-Treated Human Neutrophils Contain Inducible Nitric Oxide Synthase That Produces Nitration of Ingested Bacteria. Proc Natl Acad Sci USA (1996) 93(18):9553–8. doi: 10.1073/pnas.93.18.9553 PMC38466 8790368

[36] Murphy G Reynolds JJ Bretz U Baggiolini M. Collagenase Is a Component of the Specific Granules of Human Neutrophil Leucocytes. Biochem J (1977) 162(1):195–7. doi: 10.1042/bj1620195 PMC1164583 192209

[37] Murphy G Bretz U Baggiolini M Reynolds JJ. The Latent Collagenase and Gelatinase of Human Polymorphonuclear Neutrophil Leucocytes. Biochem J (1980) 192(2):517–25. doi: 10.1042/bj1920517 PMC1162366 6263256

[38] Cowland JB Johnsen AH Borregaard N. hCAP-18, a Cathelin/Pro-Bactenecin-Like Protein of Human Neutrophil Specific Granules. FEBS Lett (1995) 368(1):173–6. doi: 10.1016/0014-5793(95)00634-L 7615076

[39] Wright HL Moots RJ Bucknall RC Edwards SW. Neutrophil Function in Inflammation and Inflammatory Diseases. Rheumatol (Oxford) (2010) 49(9):1618–31. doi: 10.1093/rheumatology/keq045 20338884

[40] Lukasova E Koristek Z Klabusay M Ondrej V Grigoryev S Bacikova A. Granulocyte Maturation Determines Ability to Release Chromatin NETs and Loss of DNA Damage Response; These Properties Are Absent in Immature AML Granulocytes. Biochim Biophys Acta (2013) 1833(3):767–79. doi: 10.1016/j.bbamcr.2012.12.012 23269287

[41] Steinberg BE Grinstein S. Unconventional Roles of the NADPH Oxidase: Signaling, Ion Homeostasis, and Cell Death. Sci STKE (2007) 2007(379):pe11. doi: 10.1126/stke.3792007pe11 17392241

[42] Guimaraes-Costa AB Nascimento MT Froment GS Soares RP Morgado FN Conceicao-Silva F. Leishmania Amazonensis Promastigotes Induce and are Killed by Neutrophil Extracellular Traps. Proc Natl Acad Sci USA (2009) 106(16):6748–53. doi: 10.1073/pnas.0900226106 PMC2672475 19346483

[43] Kobayashi SD Malachowa N DeLeo FR. Neutrophils and Bacterial Immune Evasion. J Innate Immun (2018) 10(5-6):432–41. doi: 10.1159/000487756 PMC6784029 29642066

[44] Gupta AK Joshi MB Philippova M Erne P Hasler P Hahn S. Activated Endothelial Cells Induce Neutrophil Extracellular Traps and are Susceptible to NETosis-Mediated Cell Death. FEBS Lett (2010) 584(14):3193–7. doi: 10.1016/j.febslet.2010.06.006 20541553

[45] Margraf S Logters T Reipen J Altrichter J Scholz M Windolf J. Neutrophil-Derived Circulating Free DNA (Cf-DNA/NETs): A Potential Prognostic Marker for Posttraumatic Development of Inflammatory Second Hit and Sepsis. Shock (2008) 30(4):352–8. doi: 10.1097/SHK.0b013e31816a6bb1 18317404

[46] Lefrancais E Mallavia B Zhuo H Calfee CS Looney MR. Maladaptive Role of Neutrophil Extracellular Traps in Pathogen-Induced Lung Injury. JCI Insight (2018) 3(3):e98178. doi: 10.1172/jci.insight.98178 PMC5821185 29415887

[47] Maruchi Y Tsuda M Mori H Takenaka N Gocho T Huq MA. Plasma Myeloperoxidase-Conjugated DNA Level Predicts Outcomes and Organ Dysfunction in Patients With Septic Shock. Crit Care (2018) 22(1):176. doi: 10.1186/s13054-018-2109-7 30005596 PMC6045839

[48] Clark SR Ma AC Tavener SA McDonald B Goodarzi Z Kelly MM. Platelet TLR4 Activates Neutrophil Extracellular Traps to Ensnare Bacteria in Septic Blood. Nat Med (2007) 13(4):463–9. doi: 10.1038/nm1565 17384648

[49] Fuchs TA Abed U Goosmann C Hurwitz R Schulze I Wahn V. Novel Cell Death Program Leads to Neutrophil Extracellular Traps. J Cell Biol (2007) 176(2):231–41. doi: 10.1083/jcb.200606027 PMC2063942 17210947

[50] Chow OA von Kockritz-Blickwede M Bright AT Hensler ME Zinkernagel AS Cogen AL. Statins Enhance Formation of Phagocyte Extracellular Traps. Cell Host Microbe (2010) 8(5):445–54. doi: 10.1016/j.chom.2010.10.005 PMC3008410 21075355

[51] Kessenbrock K Krumbholz M Schonermarck U Back W Gross WL Werb Z. Netting Neutrophils in Autoimmune Small-Vessel Vasculitis. Nat Med (2009) 15(6):623–5. doi: 10.1038/nm.1959 PMC2760083 19448636

[52] Yousefi S Mihalache C Kozlowski E Schmid I Simon HU. Viable Neutrophils Release Mitochondrial DNA to Form Neutrophil Extracellular Traps. Cell Death Differ (2009) 16(11):1438–44. doi: 10.1038/cdd.2009.96 19609275

[53] Pilsczek FH Salina D Poon KK Fahey C Yipp BG Sibley CD. A Novel Mechanism of Rapid Nuclear Neutrophil Extracellular Trap Formation in Response to Staphylococcus Aureus. J Immunol (2010) 185(12):7413–25. doi: 10.4049/jimmunol.1000675 21098229

[54] Yipp BG Petri B Salina D Jenne CN Scott BN Zbytnuik LD. Infection-Induced NETosis Is a Dynamic Process Involving Neutrophil Multitasking *In Vivo* . Nat Med (2012) 18(9):1386–93. doi: 10.1038/nm.2847 PMC4529131 22922410

[55] Hakkim A Fuchs TA Martinez NE Hess S Prinz H Zychlinsky A. Activation of the Raf-MEK-ERK Pathway Is Required for Neutrophil Extracellular Trap Formation. Nat Chem Biol (2011) 7(2):75–7. doi: 10.1038/nchembio.496 21170021

[56] Awasthi D Nagarkoti S Kumar A Dubey M Singh AK Pathak P. Oxidized LDL Induced Extracellular Trap Formation in Human Neutrophils *via* TLR-PKC-IRAK-MAPK and NADPH-Oxidase Activation. Free Radic Biol Med (2016) 93:190–203. doi: 10.1016/j.freeradbiomed.2016.01.004 26774674

[57] Neeli I Radic M. Opposition Between PKC Isoforms Regulates Histone Deimination and Neutrophil Extracellular Chromatin Release. Front Immunol (2013) 4:38. doi: 10.3389/fimmu.2013.00038 23430963 PMC3576869

[58] DeSouza-Vieira T Guimaraes-Costa A Rochael NC Lira MN Nascimento MT Lima-Gomez PS. Neutrophil Extracellular Traps Release Induced by Leishmania: Role of PI3Kgamma, ERK, PI3Ksigma, PKC, and [Ca2+]. J Leukoc Biol (2016) 100(4):801–10. doi: 10.1189/jlb.4A0615-261RR PMC5014744 27154356

[59] Douda DN Yip L Khan MA Grasemann H Palaniyar N. Akt is Essential to Induce NADPH-Dependent NETosis and to Switch the Neutrophil Death to Apoptosis. Blood (2014) 123(4):597–600. doi: 10.1182/blood-2013-09-526707 24458280

[60] Martinelli S Urosevic M Daryadel A Oberholzer PA Baumann C Fey MF. Induction of Genes Mediating Interferon-Dependent Extracellular Trap Formation During Neutrophil Differentiation. J Biol Chem (2004) 279(42):44123–32. doi: 10.1074/jbc.M405883200 15302890

[61] Keshari RS Jyoti A Dubey M Kothari N Kohli M Bogra J. Cytokines Induced Neutrophil Extracellular Traps Formation: Implication for the Inflammatory Disease Condition. PloS One (2012) 7(10):e48111. doi: 10.1371/journal.pone.0048111 23110185 PMC3482178

[62] Remijsen Q Vanden Berghe T Wirawan E Asselbergh B Parthoens E De Rycke R. Neutrophil Extracellular Trap Cell Death Requires Both Autophagy and Superoxide Generation. Cell Res (2011) 21(2):290–304. doi: 10.1038/cr.2010.150 21060338 PMC3193439

[63] Urban CF Reichard U Brinkmann V Zychlinsky A. Neutrophil Extracellular Traps Capture and Kill Candida Albicans Yeast and Hyphal Forms. Cell Microbiol (2006) 8(4):668–76. doi: 10.1111/j.1462-5822.2005.00659.x 16548892

[64] Ramos-Kichik V Mondragon-Flores R Mondragon-Castelan M Gonzalez-Pozos S Muniz-Hernandez S Rojas-Espinosa O. Neutrophil Extracellular Traps Are Induced by Mycobacterium Tuberculosis. Tuberculosis (Edinb) (2009) 89(1):29–37. doi: 10.1016/j.tube.2008.09.009 19056316

[65] Chen K Nishi H Travers R Tsuboi N Martinod K Wagner DD. Endocytosis of Soluble Immune Complexes Leads to Their Clearance by FcgammaRIIIB But Induces Neutrophil Extracellular Traps *via* FcgammaRIIA *In Vivo* . Blood (2012) 120(22):4421–31. doi: 10.1182/blood-2011-12-401133 PMC3507149 22955924

[66] Behnen M Leschczyk C Moller S Batel T Klinger M Solbach W. Immobilized Immune Complexes Induce Neutrophil Extracellular Trap Release by Human Neutrophil Granulocytes *via* FcgammaRIIIB and Mac-1. J Immunol (2014) 193(4):1954–65. doi: 10.4049/jimmunol.1400478 25024378

[67] Amulic B Knackstedt SL Abu Abed U Deigendesch N Harbort CJ Caffrey BE. Cell-Cycle Proteins Control Production of Neutrophil Extracellular Traps. Dev Cell (2017) 43(4):449–62.e5. doi: 10.1016/j.devcel.2017.10.013 29103955

[68] Touyz RM Chen X Tabet F Yao G He G Quinn MT. Expression of a Functionally Active Gp91phox-Containing Neutrophil-Type NAD(P)H Oxidase in Smooth Muscle Cells From Human Resistance Arteries: Regulation by Angiotensin II. Circ Res (2002) 90(11):1205–13. doi: 10.1161/01.RES.0000020404.01971.2F 12065324

[69] Panday A Sahoo MK Osorio D Batra S. NADPH Oxidases: An Overview From Structure to Innate Immunity-Associated Pathologies. Cell Mol Immunol (2015) 12(1):5–23. doi: 10.1038/cmi.2014.89 25263488 PMC4654378

[70] Khan MA Philip LM Cheung G Vadakepeedika S Grasemann H Sweezey N. Regulating NETosis: Increasing pH Promotes NADPH Oxidase-Dependent NETosis. Front Med (Lausanne) (2018) 5:19. doi: 10.3389/fmed.2018.00019 29487850 PMC5816902

[71] Metzler KD Fuchs TA Nauseef WM Reumaux D Roesler J Schulze I. Myeloperoxidase is Required for Neutrophil Extracellular Trap Formation: Implications for Innate Immunity. Blood (2011) 117(3):953–9. doi: 10.1182/blood-2010-06-290171 PMC3035083 20974672

[72] Metzler KD Goosmann C Lubojemska A Zychlinsky A Papayannopoulos V. A Myeloperoxidase-Containing Complex Regulates Neutrophil Elastase Release and Actin Dynamics During NETosis. Cell Rep (2014) 8(3):883–96. doi: 10.1016/j.celrep.2014.06.044 PMC4471680 25066128

[73] Papayannopoulos V Metzler KD Hakkim A Zychlinsky A. Neutrophil Elastase and Myeloperoxidase Regulate the Formation of Neutrophil Extracellular Traps. J Cell Biol (2010) 191(3):677–91. doi: 10.1083/jcb.201006052 PMC3003309 20974816

[74] Sollberger G Tilley DO Zychlinsky A. Neutrophil Extracellular Traps: The Biology of Chromatin Externalization. Dev Cell (2018) 44(5):542–53. doi: 10.1016/j.devcel.2018.01.019 29533770

[75] Desai J Kumar SV Mulay SR Konrad L Romoli S Schauer C. PMA and Crystal-Induced Neutrophil Extracellular Trap Formation Involves RIPK1-RIPK3-MLKL Signaling. Eur J Immunol (2016) 46(1):223–9. doi: 10.1002/eji.201545605 26531064

[76] Desai J Mulay SR Nakazawa D Anders HJ. Matters of Life and Death. How Neutrophils Die or Survive Along NET Release and is “NETosis” = Necroptosis? Cell Mol Life Sci (2016) 73(11-12):2211–9. doi: 10.1007/s00018-016-2195-0 PMC11108262 27048811

[77] Desai J Foresto-Neto O Honarpisheh M Steiger S Nakazawa D Popper B. Particles of Different Sizes and Shapes Induce Neutrophil Necroptosis Followed by the Release of Neutrophil Extracellular Trap-Like Chromatin. Sci Rep (2017) 7(1):15003. doi: 10.1038/s41598-017-15106-0 29101355 PMC5670218

[78] Khan MA Palaniyar N. Transcriptional firing helps to drive NETosis. Sci Rep (2017) 7:41749. doi: 10.1038/srep417497:41749 28176807 PMC5296899

[79] Yang H Biermann MH Brauner JM Liu Y Zhao Y Herrmann M. New Insights into Neutrophil Extracellular Traps: Mechanisms of Formation and Role in Inflammation. Front Immunol (2016) 7(302). doi: 10.3389/fimmu.2016.00302. PMC4981595 27570525

[80] Parker H Dragunow M Hampton MB Kettle AJ Winterbourn CC. Requirements for NADPH Oxidase and Myeloperoxidase in Neutrophil Extracellular Trap Formation Differ Depending on the Stimulus. J Leukoc Biol (2012) 92(4):841–9. doi: 10.1189/jlb.1211601 22802447

[81] Vorobjeva N Galkin I Pletjushkina O Golyshev S Zinovkin R Prikhodko A. Mitochondrial Permeability Transition Pore is Involved in Oxidative Burst and NETosis of Human Neutrophils. Biochim Biophys Acta Mol Basis Dis (2020) 1866(5):165664. doi: 10.1016/j.bbadis.2020.165664 31926265

[82] Douda DN Khan MA Grasemann H Palaniyar N. SK3 Channel and Mitochondrial ROS Mediate NADPH Oxidase-Independent NETosis Induced by Calcium Influx. Proc Natl Acad Sci USA (2015) 112(9):2817–22. doi: 10.1073/pnas.1414055112 PMC4352781 25730848

[83] Berger-Achituv S Brinkmann V Abed UA Kuhn LI Ben-Ezra J Elhasid R. A Proposed Role for Neutrophil Extracellular Traps in Cancer Immunoediting. Front Immunol (2013) 4:48. doi: 10.3389/fimmu.2013.00048 23508552 PMC3589747

[84] Oklu R Sheth RA Wong KHK Jahromi AH Albadawi H. Neutrophil Extracellular Traps Are Increased in Cancer Patients But Does Not Associate With Venous Thrombosis. Cardiovasc Diagn Ther (2017) 7(Suppl 3):S140–S9. doi: 10.21037/cdt.2017.08.01 PMC5778521 29399517

[85] Rayes RF Mouhanna JG Nicolau I Bourdeau F Giannias B Rousseau S. Primary Tumors Induce Neutrophil Extracellular Traps With Targetable Metastasis Promoting Effects. JCI Insight (2019) 5(16):e128008. doi: 10.1172/jci.insight.128008 PMC6777835 31343990

[86] Martins-Cardoso K Almeida VH Bagri KM Rossi MID Mermelstein CS Konig S. Neutrophil Extracellular Traps (NETs) Promote Pro-Metastatic Phenotype in Human Breast Cancer Cells Through Epithelial-Mesenchymal Transition. Cancers (Basel) (2020) 12(6):1542. doi: 10.3390/cancers12061542 PMC7352979 32545405

[87] Zhu T Zou X Yang C Li L Wang B Li R. Neutrophil Extracellular Traps Promote Gastric Cancer Metastasis by Inducing Epithelialmesenchymal Transition. Int J Mol Med (2021) 48(1):127. doi: 10.3892/ijmm.2021.4960 34013374 PMC8128417

[88] Jin W Yin H Li H Yu XJ Xu HX Liu L. Neutrophil Extracellular DNA Traps Promote Pancreatic Cancer Cells Migration and Invasion by Activating EGFR/ERK Pathway. J Cell Mol Med (2021) 25(12):5443–56. doi: 10.1111/jcmm.16555 PMC8184670 33955688

[89] Dvorak HF. Tumors: wounds that do not heal. Similarities between tumor stroma generation and wound healing. N Engl J Med (1986) 315(26):1650–9. doi: 10.1056/NEJM198612253152606 3537791

[90] Tohme S Yazdani HO Al-Khafaji AB Chidi AP Loughran P Mowen K. Neutrophil Extracellular Traps Promote the Development and Progression of Liver Metastases after Surgical Stress. Cancer Res (2016) 76(6):1367–80. doi: 10.1158/0008-5472.CAN-15-1591 PMC4794393 26759232

[91] Mahiddine K Blaisdell A Ma S Crequer-Grandhomme A Lowell CA Erlebacher A. Relief of Tumor Hypoxia Unleashes the Tumoricidal Potential of Neutrophils. J Clin Invest (2020) 130(1):389–403. doi: 10.1172/JCI130952 31600172 PMC6934192

[92] Kong T Eltzschig HK Karhausen J Colgan SP Shelley CS. Leukocyte Adhesion During Hypoxia is Mediated by HIF-1-Dependent Induction of Beta2 Integrin Gene Expression. Proc Natl Acad Sci USA (2004) 101(28):10440–5. doi: 10.1073/pnas.0401339101 PMC478589 15235127

[93] McInturff AM Cody MJ Elliott EA Glenn JW Rowley JW Rondina MT. Mammalian Target of Rapamycin Regulates Neutrophil Extracellular Trap Formation *via* Induction of Hypoxia-Inducible Factor 1 Alpha. Blood (2012) 120(15):3118–25. doi: 10.1182/blood-2012-01-405993 PMC3471519 22919032

[94] Triner D Xue X Schwartz AJ Jung I Colacino JA Shah YM. Epithelial Hypoxia-Inducible Factor 2alpha Facilitates the Progression of Colon Tumors Through Recruiting Neutrophils. Mol Cell Biol (2017) 37(5):e00481–16. doi: 10.1128/MCB.00481-16 PMC5311236 27956697

[95] Xiao Y Cong M Li J He D Wu Q Tian P. Cathepsin C Promotes Breast Cancer Lung Metastasis by Modulating Neutrophil Infiltration and Neutrophil Extracellular Trap Formation. Cancer Cell (2021) 39(3):423–37 e7. doi: 10.1016/j.ccell.2020.12.012 33450198

[96] Shang A Gu C Zhou C Yang Y Chen C Zeng B. Exosomal KRAS Mutation Promotes the Formation of Tumor-Associated Neutrophil Extracellular Traps and Causes Deterioration of Colorectal Cancer by Inducing IL-8 Expression. Cell Commun Signal (2020) 18(1):52. doi: 10.1186/s12964-020-0517-1 32228650 PMC7106821

[97] Demers M Krause DS Schatzberg D Martinod K Voorhees JR Fuchs TA. Cancers Predispose Neutrophils to Release Extracellular DNA Traps That Contribute to Cancer-Associated Thrombosis. Proc Natl Acad Sci USA (2012) 109(32):13076–81. doi: 10.1073/pnas.1200419109 PMC3420209 22826226

[98] Cedervall J Zhang Y Huang H Zhang L Femel J Dimberg A. Neutrophil Extracellular Traps Accumulate in Peripheral Blood Vessels and Compromise Organ Function in Tumor-Bearing Animals. Cancer Res (2015) 75(13):2653–62. doi: 10.1158/0008-5472.CAN-14-3299 26071254

[99] Nierodzik ML Plotkin A Kajumo F Karpatkin S. Thrombin Stimulates Tumor-Platelet Adhesion *In Vitro* and Metastasis *In Vivo* . J Clin Invest (1991) 87(1):229–36. doi: 10.1172/JCI114976 PMC295033 1845869

[100] Klepfish A Greco MA Karpatkin S. Thrombin Stimulates Melanoma Tumor-Cell Binding to Endothelial Cells and Subendothelial Matrix. Int J Cancer (1993) 53(6):978–82. doi: 10.1002/ijc.2910530620 8473056

[101] Huang YQ Li JJ Hu L Lee M Karpatkin S. Thrombin Induces Increased Expression and Secretion of VEGF From Human FS4 Fibroblasts, DU145 Prostate Cells and CHRF Megakaryocytes. Thromb Haemost (2001) 86(4):1094–8. doi: 10.1055/s-0037-1616538 11686329

[102] Najmeh S Cools-Lartigue J Rayes RF Gowing S Vourtzoumis P Bourdeau F. Neutrophil Extracellular Traps Sequester Circulating Tumor Cells *via* Beta1-Integrin Mediated Interactions. Int J Cancer (2017) 140(10):2321–30. doi: 10.1002/ijc.30635 28177522

[103] Monti M De Rosa V Iommelli F Carriero MV Terlizzi C Camerlingo R. Neutrophil Extracellular Traps as an Adhesion Substrate for Different Tumor Cells Expressing RGD-Binding Integrins. Int J Mol Sci (2018) 19(8):2350. doi: 10.3390/ijms19082350 PMC6121671 30096958

[104] Deryugina E Carre A Ardi V Muramatsu T Schmidt J Pham C. Neutrophil Elastase Facilitates Tumor Cell Intravasation and Early Metastatic Events. iScience (2020) 23(12):101799. doi: 10.1016/j.isci.2020.101799 33299970 PMC7702017

[105] Teijeira A Garasa S Gato M Alfaro C Migueliz I Cirella A. CXCR1 and CXCR2 Chemokine Receptor Agonists Produced by Tumors Induce Neutrophil Extracellular Traps That Interfere With Immune Cytotoxicity. Immunity (2020) 52(5):856–71 e8. doi: 10.1016/j.immuni.2020.03.001 32289253

[106] Yang D Liu J. Neutrophil Extracellular Traps: A New Player in Cancer Metastasis and Therapeutic Target. J Exp Clin Cancer Res (2021) 40(1):233. doi: 10.1186/s13046-021-02013-6 34271947 PMC8283906

[107] Yang LY Luo Q Lu L Zhu WW Sun HT Wei R. Increased Neutrophil Extracellular Traps Promote Metastasis Potential of Hepatocellular Carcinoma *via* Provoking Tumorous Inflammatory Response. J Hematol Oncol (2020) 13(1):3. doi: 10.1186/s13045-019-0836-0 31907001 PMC6945602

[108] Albrengues J Shields MA Ng D Park CG Ambrico A Poindexter ME. Neutrophil Extracellular Traps Produced During Inflammation Awaken Dormant Cancer Cells in Mice. Science (2018) 361(6409):eaao4227. doi: 10.1126/science.aao4227 30262472 PMC6777850

[109] Kaltenmeier C Yazdani HO Morder K Geller DA Simmons RL Tohme S. Neutrophil Extracellular Traps Promote T Cell Exhaustion in the Tumor Microenvironment. Front Immunol (2021) 12:785222. doi: 10.3389/fimmu.2021.785222 34899751 PMC8652262

[110] Wang H Zhang H Wang Y Brown ZJ Xia Y Huang Z. Regulatory T-Cell and Neutrophil Extracellular Trap Interaction Contributes to Carcinogenesis in Non-Alcoholic Steatohepatitis. J Hepatol (2021) 75(6):1271–83. doi: 10.1016/j.jhep.2021.07.032 PMC12888775 34363921

[111] Shinde-Jadhav S Mansure JJ Rayes RF Marcq G Ayoub M Skowronski R. Role of Neutrophil Extracellular Traps in Radiation Resistance of Invasive Bladder Cancer. Nat Commun (2021) 12(1):2776. doi: 10.1038/s41467-021-23086-z 33986291 PMC8119713

[112] Fuchs TA Brill A Duerschmied D Schatzberg D Monestier M Myers DD Jr. Extracellular DNA Traps Promote Thrombosis. Proc Natl Acad Sci USA (2010) 107(36):15880–5. doi: 10.1073/pnas.1005743107 PMC2936604 20798043

[113] Frampton G Perl S Bennett A Cameron JS. Platelet-Associated DNA and Anti-DNA Antibody in Systemic Lupus Erythematosus With Nephritis. Clin Exp Immunol (1986) 63(3):621–8. PMC1577544 3486735

[114] Dvorak HF Van DeWater L Bitzer AM Dvorak AM Anderson D Harvey VS. Procoagulant Activity Associated With Plasma Membrane Vesicles Shed by Cultured Tumor Cells. Cancer Res (1983) 43(9):4434–42. 6347372

[115] Bastida E Ordinas A Escolar G Jamieson GA. Tissue Factor in Microvesicles Shed From U87MG Human Glioblastoma Cells Induces Coagulation, Platelet Aggregation, and Thrombogenesis. Blood (1984) 64(1):177–84. doi: 10.1182/blood.V64.1.177.177 6733271

[116] Etulain J Martinod K Wong SL Cifuni SM Schattner M Wagner DD. P-Selectin Promotes Neutrophil Extracellular Trap Formation in Mice. Blood (2015) 126(2):242–6. doi: 10.1182/blood-2015-01-624023 PMC4497964 25979951

[117] Carestia A Kaufman T Rivadeneyra L Landoni VI Pozner RG Negrotto S. Mediators and Molecular Pathways Involved in the Regulation of Neutrophil Extracellular Trap Formation Mediated by Activated Platelets. J Leukoc Biol (2016) 99(1):153–62. doi: 10.1189/jlb.3A0415-161R 26320263

[118] Maugeri N Campana L Gavina M Covino C De Metrio M Panciroli C. Activated Platelets Present High Mobility Group Box 1 to Neutrophils, Inducing Autophagy and Promoting the Extrusion of Neutrophil Extracellular Traps. J Thromb Haemost (2014) 12(12):2074–88. doi: 10.1111/jth.12710 25163512

[119] Ay C Simanek R Vormittag R Dunkler D Alguel G Koder S. High Plasma Levels of Soluble P-Selectin Are Predictive of Venous Thromboembolism in Cancer Patients: Results From the Vienna Cancer and Thrombosis Study (CATS). Blood (2008) 112(7):2703–8. doi: 10.1182/blood-2008-02-142422 18539899

[120] Dyer MR Chen Q Haldeman S Yazdani H Hoffman R Loughran P. Deep Vein Thrombosis in Mice Is Regulated by Platelet HMGB1 Through Release of Neutrophil-Extracellular Traps and DNA. Sci Rep (2018) 8(1):2068. doi: 10.1038/s41598-018-20479-x 29391442 PMC5794752

[121] Nakazawa D Desai J Steiger S Muller S Devarapu SK Mulay SR. Activated Platelets Induce MLKL-Driven Neutrophil Necroptosis and Release of Neutrophil Extracellular Traps in Venous Thrombosis. Cell Death Discovery (2018) 4:6. doi: 10.1038/s41420-018-0073-2 PMC6060161 30062055

[122] Labelle M Begum S Hynes RO. Platelets Guide the Formation of Early Metastatic Niches. Proc Natl Acad Sci USA (2014) 111(30):E3053–61. doi: 10.1073/pnas.1411082111 PMC4121772 25024172

[123] Chen Q Zhang L Li X Zhuo W. Neutrophil Extracellular Traps in Tumor Metastasis: Pathological Functions and Clinical Applications. Cancers (Basel) (2021) 13(11):2832. doi: 10.3390/cancers13112832. 34204148 PMC8200981

[124] Mauracher LM Posch F Martinod K Grilz E Daullary T Hell L. Citrullinated Histone H3, a Biomarker of Neutrophil Extracellular Trap Formation, Predicts the Risk of Venous Thromboembolism in Cancer Patients. J Thromb Haemost (2018) 16(3):508–18. doi: 10.1111/jth.13951 PMC6294121 29325226

[125] Zhang S Guo M Liu Q Liu J Cui Y. Neutrophil Extracellular Traps Induce a Hypercoagulable State in Glioma. Immun Inflammation Dis (2021) 9(4):1383–93. doi: 10.1002/iid3.488 PMC8589396 34288521

[126] Gould TJ Vu TT Swystun LL Dwivedi DJ Mai SH Weitz JI. Neutrophil Extracellular Traps Promote Thrombin Generation Through Platelet-Dependent and Platelet-Independent Mechanisms. Arterioscler Thromb Vasc Biol (2014) 34(9):1977–84. doi: 10.1161/ATVBAHA.114.304114 25012129

[127] Boone BA Murthy P Miller-Ocuin J Doerfler WR Ellis JT Liang X. Chloroquine Reduces Hypercoagulability in Pancreatic Cancer Through Inhibition of Neutrophil Extracellular Traps. BMC Cancer (2018) 18(1):678. doi: 10.1186/s12885-018-4584-2 29929491 PMC6013899

[128] Abdol Razak N Elaskalani O Metharom P. Pancreatic Cancer-Induced Neutrophil Extracellular Traps: A Potential Contributor to Cancer-Associated Thrombosis. Int J Mol Sci (2017) 18(3):487. doi: 10.3390/ijms18030487 PMC5372503 28245569

[129] Gillis S Furie BC Furie B. Interactions of Neutrophils and Coagulation Proteins. Semin Hematol (1997) 34(4):336–42. 9347584

[130] Guglietta S Chiavelli A Zagato E Krieg C Gandini S Ravenda PS. Coagulation Induced by C3aR-Dependent NETosis Drives Protumorigenic Neutrophils During Small Intestinal Tumorigenesis. Nat Commun (2016) 7:11037. doi: 10.1038/ncomms11037 26996437 PMC4802169

[131] Moser AR Pitot HC Dove WF. A Dominant Mutation That Predisposes to Multiple Intestinal Neoplasia in the Mouse. Science (1990) 247(4940):322–4. doi: 10.1126/science.2296722 2296722

[132] Wolach O Sellar RS Martinod K Cherpokova D McConkey M Chappell RJ. Increased Neutrophil Extracellular Trap Formation Promotes Thrombosis in Myeloproliferative Neoplasms. Sci Transl Med (2018) 10(436):eaan8292. doi: 10.1126/scitranslmed.aan8292 29643232 PMC6442466

[133] Gomes T Varady CBS Lourenco AL Mizurini DM Rondon AMR Leal AC. IL-1beta Blockade Attenuates Thrombosis in a Neutrophil Extracellular Trap-Dependent Breast Cancer Model. Front Immunol (2019) 10:2088. doi: 10.3389/fimmu.2019.02088 31552036 PMC6737452

[134] Thalin C Demers M Blomgren B Wong SL von Arbin M von Heijne A. NETosis Promotes Cancer-Associated Arterial Microthrombosis Presenting as Ischemic Stroke With Troponin Elevation. Thromb Res (2016) 139:56–64. doi: 10.1016/j.thromres.2016.01.009 26916297 PMC4769435

[135] Perdomo J Leung HHL Ahmadi Z Yan F Chong JJH Passam FH. Neutrophil Activation and NETosis are the Major Drivers of Thrombosis in Heparin-Induced Thrombocytopenia. Nat Commun (2019) 10(1):1322. doi: 10.1038/s41467-019-09160-7 30899022 PMC6428879

[136] Mysore V Cullere X Mears J Rosetti F Okubo K Liew PX. FcgammaR Engagement Reprograms Neutrophils Into Antigen Cross-Presenting Cells That Elicit Acquired Anti-Tumor Immunity. Nat Commun (2021) 12(1):4791. doi: 10.1038/s41467-021-24591-x 34373452 PMC8352912

[137] Ackermann M Anders HJ Bilyy R Bowlin GL Daniel C De Lorenzo R. Patients With COVID-19: In the Dark-NETs of Neutrophils. Cell Death Differ (2021) 28(11):3125–39. doi: 10.1038/s41418-021-00805-z PMC8142290 34031543

[138] Arcanjo A Logullo J Menezes CCB de Souza Carvalho Giangiarulo TC Dos Reis MC de Castro GMM. The Emerging Role of Neutrophil Extracellular Traps in Severe Acute Respiratory Syndrome Coronavirus 2 (COVID-19). Sci Rep (2020) 10(1):19630. doi: 10.1038/s41598-020-76781-0 33184506 PMC7665044

[139] Uribe Echevarria L Leimgruber C Garcia Gonzalez J Nevado A Alvarez R Garcia LN. Evidence of Eosinophil Extracellular Trap Cell Death in COPD: Does it Represent the Trigger That Switches on the Disease? Int J Chron Obstruct Pulmon Dis (2017) 12:885–96. doi: 10.2147/COPD.S115969 PMC5359000 28352169

[140] Simon D Hoesli S Roth N Staedler S Yousefi S Simon HU. Eosinophil Extracellular DNA Traps in Skin Diseases. J Allergy Clin Immunol (2011) 127(1):194–9. doi: 10.1016/j.jaci.2010.11.002 21211654

[141] Marx C Novotny J Salbeck D Zellner KR Nicolai L Pekayvaz K. Eosinophil-Platelet Interactions Promote Atherosclerosis and Stabilize Thrombosis With Eosinophil Extracellular Traps. Blood (2019) 134(21):1859–72. doi: 10.1182/blood.2019000518 PMC6908806 31481482

[142] Pertiwi KR de Boer OJ Mackaaij C Pabittei DR de Winter RJ Li X. Extracellular Traps Derived From Macrophages, Mast Cells, Eosinophils and Neutrophils are Generated in a Time-Dependent Manner During Atherothrombosis. J Pathol (2019) 247(4):505–12. doi: 10.1002/path.5212 PMC6590313 30506885

[143] Muniz VS Silva JC Braga YAV Melo RCN Ueki S Takeda M. Eosinophils Release Extracellular DNA Traps in Response to Aspergillus Fumigatus. J Allergy Clin Immunol (2018) 141(2):571–85.e7. doi: 10.1016/j.jaci.2017.07.048 28943470

[144] Yousefi S Gold JA Andina N Lee JJ Kelly AM Kozlowski E. Catapult-Like Release of Mitochondrial DNA by Eosinophils Contributes to Antibacterial Defense. Nat Med (2008) 14(9):949–53. doi: 10.1038/nm.1855 18690244

[145] Morshed M Yousefi S Stockle C Simon HU Simon D. Thymic Stromal Lymphopoietin Stimulates the Formation of Eosinophil Extracellular Traps. Allergy (2012) 67(9):1127–37. doi: 10.1111/j.1398-9995.2012.02868.x 22764833

[146] Germic N Stojkov D Oberson K Yousefi S Simon HU. Neither Eosinophils Nor Neutrophils Require ATG5-Dependent Autophagy for Extracellular DNA Trap Formation. Immunology (2017) 152(3):517–25. doi: 10.1111/imm.12790 PMC5629432 28703297

[147] Ueki S Melo RC Ghiran I Spencer LA Dvorak AM Weller PF. Eosinophil Extracellular DNA Trap Cell Death Mediates Lytic Release of Free Secretion-Competent Eosinophil Granules in Humans. Blood (2013) 121(11):2074–83. doi: 10.1182/blood-2012-05-432088 PMC3596967 23303825

[148] Ueki S Konno Y Takeda M Moritoki Y Hirokawa M Matsuwaki Y. Eosinophil Extracellular Trap Cell Death-Derived DNA Traps: Their Presence in Secretions and Functional Attributes. J Allergy Clin Immunol (2016) 137(1):258–67. doi: 10.1016/j.jaci.2015.04.041 PMC4674385 26070883

[149] Kim HJ Sim MS Lee DH Kim C Choi Y Park HS. Lysophosphatidylserine Induces Eosinophil Extracellular Trap Formation and Degranulation: Implications in Severe Asthma. Allergy (2020) 75(12):3159–70. doi: 10.1111/all.14450 32535937

[150] Rothenberg ME Hogan SP. The Eosinophil. Annu Rev Immunol (2006) 24:147–74. doi: 10.1146/annurev.immunol.24.021605.090720 16551246

[151] Choi Y Le Pham D Lee DH Lee SH Kim SH Park HS. Biological Function of Eosinophil Extracellular Traps in Patients With Severe Eosinophilic Asthma. Exp Mol Med (2018) 50(8):1–8. doi: 10.1038/s12276-018-0136-8 PMC6095846 30115903

[152] Palframan RT Collins PD Severs NJ Rothery S Williams TJ Rankin SM. Mechanisms of Acute Eosinophil Mobilization From the Bone Marrow Stimulated by Interleukin 5: The Role of Specific Adhesion Molecules and Phosphatidylinositol 3-Kinase. J Exp Med (1998) 188(9):1621–32. doi: 10.1084/jem.188.9.1621 PMC2212511 9802974

[153] Lundahl J Sehmi R Hayes L Howie K Denburg JA. Selective Upregulation of a Functional Beta7 Integrin on Differentiating Eosinophils. Allergy (2000) 55(9):865–72. doi: 10.1034/j.1398-9995.2000.00574.x 11003451

[154] Lundahl J Sehmi R Moshfegh A Hayes L Howie K Upham J. Distinct Phenotypic Adhesion Molecule Expression on Human Cord Blood Progenitors During Early Eosinophilic Commitment: Upregulation of Beta(7) Integrins. Scand J Immunol (2002) 56(2):161–7. doi: 10.1046/j.1365-3083.2002.01117.x 12121435

[155] Gaspar-Elsas MI Queto T Vasconcelos Z Jones CP Lannes-Vieira J Xavier-Elsas P. Evidence for a Regulatory Role of Alpha 4-Integrins in the Maturation of Eosinophils Generated From the Bone Marrow in the Presence of Dexamethasone. Clin Exp Allergy (2009) 39(8):1187–98. doi: 10.1111/j.1365-2222.2009.03289.x 19508325

[156] Chu DK Jimenez-Saiz R Verschoor CP Walker TD Goncharova S Llop-Guevara A. Indigenous Enteric Eosinophils Control DCs to Initiate a Primary Th2 Immune Response *In Vivo* . J Exp Med (2014) 211(8):1657–72. doi: 10.1084/jem.20131800 PMC4113937 25071163

[157] Mukherjee M Bulir DC Radford K Kjarsgaard M Huang CM Jacobsen EA. Sputum Autoantibodies in Patients With Severe Eosinophilic Asthma. J Allergy Clin Immunol (2018) 141(4):1269–79. doi: 10.1016/j.jaci.2017.06.033 28751233

[158] Rohrbach MS Wheatley CL Slifman NR Gleich GJ. Activation of Platelets by Eosinophil Granule Proteins. J Exp Med (1990) 172(4):1271–4. doi: 10.1084/jem.172.4.1271 PMC2188607 2212954

[159] Moosbauer C Morgenstern E Cuvelier SL Manukyan D Bidzhekov K Albrecht S. Eosinophils Are a Major Intravascular Location for Tissue Factor Storage and Exposure. Blood (2007) 109(3):995–1002. doi: 10.1182/blood-2006-02-004945 17003379

[160] Francischetti IMB Alejo JC Sivanandham R Davies-Hill T Fetsch P Pandrea I. Neutrophil and Eosinophil Extracellular Traps in Hodgkin Lymphoma. Hemasphere (2021) 5(9):e633. doi: 10.1097/HS9.0000000000000633 34485830 PMC8410234

[161] Enblad G Sundstrom C Glimelius B. Infiltration of Eosinophils in Hodgkin’s Disease Involved Lymph Nodes Predicts Prognosis. Hematol Oncol (1993) 11(4):187–93. doi: 10.1002/hon.2900110404 8144133

[162] Sakkal S Miller S Apostolopoulos V Nurgali K. Eosinophils in Cancer: Favourable or Unfavourable? Curr Med Chem (2016) 23(7):650–66. doi: 10.2174/0929867323666160119094313 26785997

[163] Reichman H Karo-Atar D Munitz A. Emerging Roles for Eosinophils in the Tumor Microenvironment. Trends Cancer (2016) 2(11):664–75. doi: 10.1016/j.trecan.2016.10.002 28741505

[164] Menzies-Gow A Ying S Sabroe I Stubbs VL Soler D Williams TJ. Eotaxin (CCL11) and Eotaxin-2 (CCL24) Induce Recruitment of Eosinophils, Basophils, Neutrophils, and Macrophages as Well as Features of Early- and Late-Phase Allergic Reactions Following Cutaneous Injection in Human Atopic and Nonatopic Volunteers. J Immunol (2002) 169(5):2712–8. doi: 10.4049/jimmunol.169.5.2712 12193745

[165] Cho H Lim SJ Won KY Bae GE Kim GY Min JW. Eosinophils in Colorectal Neoplasms Associated With Expression of CCL11 and CCL24. J Pathol Transl Med (2016) 50(1):45–51. doi: 10.4132/jptm.2015.10.16 26657310 PMC4734969

[166] Diny NL Hou X Barin JG Chen G Talor MV Schaub J. Macrophages and Cardiac Fibroblasts are the Main Producers of Eotaxins and Regulate Eosinophil Trafficking to the Heart. Eur J Immunol (2016) 46(12):2749–60. doi: 10.1002/eji.201646557 PMC5404278 27621211

[167] Aldinucci D Lorenzon D Cattaruzza L Pinto A Gloghini A Carbone A. Expression of CCR5 Receptors on Reed-Sternberg Cells and Hodgkin Lymphoma Cell Lines: Involvement of CCL5/Rantes in Tumor Cell Growth and Microenvironmental Interactions. Int J Cancer (2008) 122(4):769–76. doi: 10.1002/ijc.23119 17935139

[168] da Silva JM Moreira Dos Santos TP Sobral LM Queiroz-Junior CM Rachid MA Proudfoot AEI. Relevance of CCL3/CCR5 Axis in Oral Carcinogenesis. Oncotarget (2017) 8(31):51024–36. doi: 10.18632/oncotarget.16882 PMC5584227 28881626

[169] Cherry WB Yoon J Bartemes KR Iijima K Kita H. A Novel IL-1 Family Cytokine, IL-33, Potently Activates Human Eosinophils. J Allergy Clin Immunol (2008) 121(6):1484–90. doi: 10.1016/j.jaci.2008.04.005 PMC2821937 18539196

[170] Curran CS Bertics PJ. Human Eosinophils Express RAGE, Produce RAGE Ligands, Exhibit PKC-Delta Phosphorylation and Enhanced Viability in Response to the RAGE Ligand, S100B. Int Immunol (2011) 23(12):713–28. doi: 10.1093/intimm/dxr083 PMC3226529 22025532

[171] Lucarini V Ziccheddu G Macchia I La Sorsa V Peschiaroli F Buccione C. IL-33 Restricts Tumor Growth and Inhibits Pulmonary Metastasis in Melanoma-Bearing Mice Through Eosinophils. Oncoimmunology (2017) 6(6):e1317420. doi: 10.1080/2162402X.2017.1317420 28680750 PMC5486175

[172] Dennis KL Wang Y Blatner NR Wang S Saadalla A Trudeau E. Adenomatous Polyps are Driven by Microbe-Instigated Focal Inflammation and are Controlled by IL-10-Producing T Cells. Cancer Res (2013) 73(19):5905–13. doi: 10.1158/0008-5472.CAN-13-1511 PMC4322779 23955389

[173] Loures FV Rohm M Lee CK Santos E Wang JP Specht CA. Recognition of Aspergillus Fumigatus Hyphae by Human Plasmacytoid Dendritic Cells Is Mediated by Dectin-2 and Results in Formation of Extracellular Traps. PloS Pathog (2015) 11(2):e1004643. doi: 10.1371/journal.ppat.1004643 25659141 PMC4450068

[174] Garcia-Romo GS Caielli S Vega B Connolly J Allantaz F Xu Z. Netting Neutrophils are Major Inducers of Type I IFN Production in Pediatric Systemic Lupus Erythematosus. Sci Transl Med (2011) 3(73):73ra20. doi: 10.1126/scitranslmed.3001201 PMC3143837 21389264

[175] Fu C Jiang A. Dendritic Cells and CD8 T Cell Immunity in Tumor Microenvironment. Front Immunol (2018) 9:3059. doi: 10.3389/fimmu.2018.03059 30619378 PMC6306491

[176] Parackova Z Zentsova I Vrabcova P Klocperk A Sumnik Z Pruhova S. Neutrophil Extracellular Trap Induced Dendritic Cell Activation Leads to Th1 Polarization in Type 1 Diabetes. Front Immunol (2020) 11:661. doi: 10.3389/fimmu.2020.00661 32346380 PMC7172866

[177] Parihar A Eubank TD Doseff AI. Monocytes and Macrophages Regulate Immunity Through Dynamic Networks of Survival and Cell Death. J Innate Immun (2010) 2(3):204–15. doi: 10.1159/000296507 PMC2956013 20375558

[178] Aulik NA Hellenbrand KM Czuprynski CJ. Mannheimia Haemolytica and its Leukotoxin Cause Macrophage Extracellular Trap Formation by Bovine Macrophages. Infect Immun (2012) 80(5):1923–33. doi: 10.1128/IAI.06120-11 PMC3347434 22354029

[179] Hellenbrand KM Forsythe KM Rivera-Rivas JJ Czuprynski CJ Aulik NA. Histophilus Somni Causes Extracellular Trap Formation by Bovine Neutrophils and Macrophages. Microb Pathog (2013) 54:67–75. doi: 10.1016/j.micpath.2012.09.007 23022668 PMC7125803

[180] Granger V Faille D Marani V Noel B Gallais Y Szely N. Human Blood Monocytes Are Able to Form Extracellular Traps. J Leukoc Biol (2017) 102(3):775–81. doi: 10.1189/jlb.3MA0916-411R 28465447

[181] Jonsson BE Bylund J Johansson BR Telemo E Wold AE. Cord-Forming Mycobacteria Induce DNA Meshwork Formation by Human Peripheral Blood Mononuclear Cells. Pathog Dis (2013) 67(1):54–66. doi: 10.1111/2049-632X.12007 23620120

[182] Wong KW Jacobs WR Jr. Mycobacterium Tuberculosis Exploits Human Interferon Gamma to Stimulate Macrophage Extracellular Trap Formation and Necrosis. J Infect Dis (2013) 208(1):109–19. doi: 10.1093/infdis/jit097 PMC3666134 23475311

[183] Agrawal I Sharma N Saxena S Arvind S Chakraborty D Chakraborty DB. Dopamine Induces Functional Extracellular Traps in Microglia. iScience (2021) 24(1):101968. doi: 10.1016/j.isci.2020.101968 33458617 PMC7797945

[184] Doster RS Sutton JA Rogers LM Aronoff DM Gaddy JA. Streptococcus Agalactiae Induces Placental Macrophages To Release Extracellular Traps Loaded With Tissue Remodeling Enzymes *via* an Oxidative Burst-Dependent Mechanism. mBio (2018) 9(6):e02084–18. doi: 10.1128/mBio.02084-18. PMC6247082 30459195

[185] Okubo K Kurosawa M Kamiya M Urano Y Suzuki A Yamamoto K. Macrophage Extracellular Trap Formation Promoted by Platelet Activation Is a Key Mediator of Rhabdomyolysis-Induced Acute Kidney Injury. Nat Med (2018) 24(2):232–8. doi: 10.1038/nm.4462 29309057

[186] O’Sullivan KM Lo CY Summers SA Elgass KD McMillan PJ Longano A. Renal Participation of Myeloperoxidase in Antineutrophil Cytoplasmic Antibody (ANCA)-Associated Glomerulonephritis. Kidney Int (2015) 88(5):1030–46. doi: 10.1038/ki.2015.202 26176828

[187] Liu P Wu X Liao C Liu X Du J Shi H. Escherichia Coli and Candida Albicans Induced Macrophage Extracellular Trap-Like Structures With Limited Microbicidal Activity. PloS One (2014) 9(2):e90042. doi: 10.1371/journal.pone.0090042 24587206 PMC3934966

[188] Munoz-Caro T Silva LM Ritter C Taubert A Hermosilla C. Besnoitia Besnoiti Tachyzoites Induce Monocyte Extracellular Trap Formation. Parasitol Res (2014) 113(11):4189–97. doi: 10.1007/s00436-014-4094-3 25193048

[189] Perez D Munoz MC Molina JM Munoz-Caro T Silva LM Taubert A. Eimeria Ninakohlyakimovae Induces NADPH Oxidase-Dependent Monocyte Extracellular Trap Formation and Upregulates IL-12 and TNF-Alpha, IL-6 and CCL2 Gene Transcription. Vet Parasitol (2016) 227:143–50. doi: 10.1016/j.vetpar.2016.07.028 27523951

[190] King PT Sharma R O’Sullivan KM Callaghan J Dousha L Thomas B. Deoxyribonuclease 1 Reduces Pathogenic Effects of Cigarette Smoke Exposure in the Lung. Sci Rep (2017) 7(1):12128. doi: 10.1038/s41598-017-12474-5 28935869 PMC5608940

[191] Graca-Souza AV Arruda MA de Freitas MS Barja-Fidalgo C Oliveira PL. Neutrophil Activation by Heme: Implications for Inflammatory Processes. Blood (2002) 99(11):4160–5. doi: 10.1182/blood.V99.11.4160 12010821

[192] Bourne JH Colicchia M Di Y Martin E Slater A Roumenina LT. Heme Induces Human and Mouse Platelet Activation Through C-Type-Lectin-Like Receptor-2. Haematologica (2021) 106(2):626–9. doi: 10.3324/haematol.2020.246488 PMC7849553 32354867

[193] Oishi S Tsukiji N Otake S Oishi N Sasaki T Shirai T. Heme Activates Platelets and Exacerbates Rhabdomyolysis-Induced Acute Kidney Injury *via* CLEC-2 and GPVI/FcRgamma. Blood Adv (2021) 5(7):2017–26. doi: 10.1182/bloodadvances.2020001698 PMC8045506 33843987

[194] Weisberg SP McCann D Desai M Rosenbaum M Leibel RL Ferrante AW Jr. Obesity is Associated With Macrophage Accumulation in Adipose Tissue. J Clin Invest (2003) 112(12):1796–808. doi: 10.1172/JCI200319246 PMC296995 14679176

[195] Cinti S Mitchell G Barbatelli G Murano I Ceresi E Faloia E. Adipocyte Death Defines Macrophage Localization and Function in Adipose Tissue of Obese Mice and Humans. J Lipid Res (2005) 46(11):2347–55. doi: 10.1194/jlr.M500294-JLR200 16150820

[196] Suganami T Nishida J Ogawa Y. A Paracrine Loop Between Adipocytes and Macrophages Aggravates Inflammatory Changes: Role of Free Fatty Acids and Tumor Necrosis Factor Alpha. Arterioscler Thromb Vasc Biol (2005) 25(10):2062–8. doi: 10.1161/01.ATV.0000183883.72263.13 16123319

[197] Berthou F Ceppo F Dumas K Massa F Vergoni B Alemany S. The Tpl2 Kinase Regulates the COX-2/Prostaglandin E2 Axis in Adipocytes in Inflammatory Conditions. Mol Endocrinol (2015) 29(7):1025–36. doi: 10.1210/me.2015-1027 PMC5414708 26020725

[198] Mohanan S Horibata S McElwee JL Dannenberg AJ Coonrod SA. Identification of Macrophage Extracellular Trap-Like Structures in Mammary Gland Adipose Tissue: A Preliminary Study. Front Immunol (2013) 4:67. doi: 10.3389/fimmu.2013.00067 23508122 PMC3600535

[199] Qian BZ Pollard JW. Macrophage Diversity Enhances Tumor Progression and Metastasis. Cell (2010) 141(1):39–51. doi: 10.1016/j.cell.2010.03.014 20371344 PMC4994190

[200] Xu SS Li H Li TJ Li S Xia HY Long J. Neutrophil Extracellular Traps and Macrophage Extracellular Traps Predict Postoperative Recurrence in Resectable Nonfunctional Pancreatic Neuroendocrine Tumors. Front Immunol (2021) 12:577517. doi: 10.3389/fimmu.2021.577517 34084158 PMC8168461

[201] Chen T Wang Y Nan Z Wu J Li A Zhang T. Interaction Between Macrophage Extracellular Traps and Colon Cancer Cells Promotes Colon Cancer Invasion and Correlates With Unfavorable Prognosis. Front Immunol (2021) 12:779325. doi: 10.3389/fimmu.2021.779325 34925357 PMC8671452

[202] Halder LD Abdelfatah MA Jo EA Jacobsen ID Westermann M Beyersdorf N. Factor H Binds to Extracellular DNA Traps Released From Human Blood Monocytes in Response to Candida Albicans. Front Immunol (2016) 7:671. doi: 10.3389/fimmu.2016.00671 28133459 PMC5233719

[203] Je S Quan H Yoon Y Na Y Kim BJ Seok SH. Mycobacterium Massiliense Induces Macrophage Extracellular Traps With Facilitating Bacterial Growth. PloS One (2016) 11(5):e0155685. doi: 10.1371/journal.pone.0155685 27191593 PMC4871462

[204] Schulz M Zambrano F Schuppe HC Wagenlehner F Taubert A Gaertner U. Monocyte-Derived Extracellular Trap (MET) Formation Induces Aggregation and Affects Motility of Human Spermatozoa *In Vitro* . Syst Biol Reprod Med (2019) 65(5):357–66. doi: 10.1080/19396368.2019.1624873 31208212

[205] Kim T de Oliveira Silva Lautenschlager S Ma Q Eller K Pollheimer MJ Lazarin-Bidoia D. Drug Crystal-Related Gastrointestinal Complications Involve Crystal-Induced Release of Neutrophil and Monocyte Extracellular Traps. Cells (2020) 9(11):2481. doi: 10.3390/cells9112481. PMC7697008 33203124

[206] Barbara G Barbaro MR Fuschi D Palombo M Falangone F Cremon C. Inflammatory and Microbiota-Related Regulation of the Intestinal Epithelial Barrier. Front Nutr (2021) 8:718356. doi: 10.3389/fnut.2021.718356 34589512 PMC8475765

[207] Wernersson S Pejler G. Mast Cell Secretory Granules: Armed for Battle. Nat Rev Immunol (2014) 14(7):478–94. doi: 10.1038/nri3690 24903914

[208] Moon TC Befus AD Kulka M. Mast Cell Mediators: Their Differential Release and the Secretory Pathways Involved. Front Immunol (2014) 5:569. doi: 10.3389/fimmu.2014.00569 25452755 PMC4231949

[209] von Kockritz-Blickwede M Goldmann O Thulin P Heinemann K Norrby-Teglund A Rohde M. Phagocytosis-Independent Antimicrobial Activity of Mast Cells by Means of Extracellular Trap Formation. Blood (2008) 111(6):3070–80. doi: 10.1182/blood-2007-07-104018 18182576

[210] Branitzki-Heinemann K Okumura CY Vollger L Kawakami Y Kawakami T Naim HY. A Novel Role for the Transcription Factor HIF-1alpha in the Formation of Mast Cell Extracellular Traps. Biochem J (2012) 446(1):159–63. doi: 10.1042/BJ20120658 PMC3606900 22738198

[211] Jeong HJ Oh HA Nam SY Han NR Kim YS Kim JH. The Critical Role of Mast Cell-Derived Hypoxia-Inducible Factor-1alpha in Human and Mice Melanoma Growth. Int J Cancer (2013) 132(11):2492–501. doi: 10.1002/ijc.27937 23161568

[212] Coussens LM Raymond WW Bergers G Laig-Webster M Behrendtsen O Werb Z. Inflammatory Mast Cells Up-Regulate Angiogenesis During Squamous Epithelial Carcinogenesis. Genes Dev (1999) 13(11):1382–97. doi: 10.1101/gad.13.11.1382 PMC316772 10364156

[213] Lilla JN Werb Z. Mast Cells Contribute to the Stromal Microenvironment in Mammary Gland Branching Morphogenesis. Dev Biol (2010) 337(1):124–33. doi: 10.1016/j.ydbio.2009.10.021 PMC2787992 19850030

[214] Mangia A Malfettone A Rossi R Paradiso A Ranieri G Simone G. Tissue Remodelling in Breast Cancer: Human Mast Cell Tryptase as an Initiator of Myofibroblast Differentiation. Histopathology (2011) 58(7):1096–106. doi: 10.1111/j.1365-2559.2011.03842.x 21707711

[215] Marone G Borriello F Varricchi G Genovese A Granata F. Basophils: Historical Reflections and Perspectives. Chem Immunol Allergy (2014) 100:172–92. doi: 10.1159/000358734 24925398

[216] Schroeder JT Bieneman AP. Activation of Human Basophils by A549 Lung Epithelial Cells Reveals a Novel IgE-Dependent Response Independent of Allergen. J Immunol (2017) 199(3):855–65. doi: 10.4049/jimmunol.1700055 PMC5541892 28652400

[217] Brunner T de Weck AL Dahinden CA. Platelet-Activating Factor Induces Mediator Release by Human Basophils Primed With IL-3, Granulocyte-Macrophage Colony-Stimulating Factor, or IL-5. J Immunol (1991) 147(1):237–42. 1711077

[218] Yousefi S Morshed M Amini P Stojkov D Simon D von Gunten S. Basophils Exhibit Antibacterial Activity Through Extracellular Trap Formation. Allergy (2015) 70(9):1184–8. doi: 10.1111/all.12662 26043360

[219] Marone G Gambardella AR Mattei F Mancini J Schiavoni G Varricchi G. Basophils in Tumor Microenvironment and Surroundings. Adv Exp Med Biol (2020) 1224:21–34. doi: 10.1007/978-3-030-35723-8_2 32036602

[220] Cohen M Giladi A Gorki AD Solodkin DG Zada M Hladik A. Lung Single-Cell Signaling Interaction Map Reveals Basophil Role in Macrophage Imprinting. Cell (2018) 175(4):1031–44 e18. doi: 10.1016/j.cell.2018.09.009 30318149

[221] Sektioglu IM Carretero R Bulbuc N Bald T Tuting T Rudensky AY. Basophils Promote Tumor Rejection *via* Chemotaxis and Infiltration of CD8+ T Cells. Cancer Res (2017) 77(2):291–302. doi: 10.1158/0008-5472.CAN-16-0993 27879269

[222] Damsker JM Hansen AM Caspi RR. Th1 and Th17 Cells: Adversaries and Collaborators. Ann N Y Acad Sci (2010) 1183:211–21. doi: 10.1111/j.1749-6632.2009.05133.x PMC2914500 20146717

[223] Bailey SR Nelson MH Himes RA Li Z Mehrotra S Paulos CM. Th17 Cells in Cancer: The Ultimate Identity Crisis. Front Immunol (2014) 5:276. doi: 10.3389/fimmu.2014.00276 24987392 PMC4060300

[224] Ye J Livergood RS Peng G. The Role and Regulation of Human Th17 Cells in Tumor Immunity. Am J Pathol (2013) 182(1):10–20. doi: 10.1016/j.ajpath.2012.08.041 23159950 PMC3532708

[225] Do Thi VA Park SM Lee H Kim YS. The Membrane-Bound Form of IL-17a Promotes the Growth and Tumorigenicity of Colon Cancer Cells. Mol Cells (2016) 39(7):536–42. doi: 10.14348/molcells.2016.0048 PMC4959018 27378226

[226] Curtis MM Way SS. Interleukin-17 in Host Defence Against Bacterial, Mycobacterial and Fungal Pathogens. Immunology (2009) 126(2):177–85. doi: 10.1111/j.1365-2567.2008.03017.x PMC2632692 19125888

[227] Agak GW Mouton A Teles RM Weston T Morselli M Andrade PR. Extracellular Traps Released by Antimicrobial TH17 Cells Contribute to Host Defense. J Clin Invest (2021) 131(2):e141594. doi: 10.1172/JCI141594 PMC7810473 33211671

[228] Rocha Arrieta YC Rojas M Vasquez G Lopez J. The Lymphocytes Stimulation Induced DNA Release, a Phenomenon Similar to NETosis. Scand J Immunol (2017) 86(4):229–38. doi: 10.1111/sji.12592 28805301

[229] Koh CC Wardini AB Vieira M Passos LSA Martinelli PM Neves EGA. Human CD8+ T Cells Release Extracellular Traps Co-Localized With Cytotoxic Vesicles That Are Associated With Lesion Progression and Severity in Human Leishmaniasis. Front Immunol (2020) 11:594581. doi: 10.3389/fimmu.2020.594581 33117407 PMC7578246

[230] Conceição-Silva F Reis CSM De Luca PM Leite-Silva J Santiago MA Morrot A. The Immune System Throws Its Traps. Cells (2021) 10(8):1891. doi: 10.3390/cells10081891 34440659 PMC8391883

[231] Shi C Kim T Steiger S Mulay SR Klinkhammer BM Bauerle T. Crystal Clots as Therapeutic Target in Cholesterol Crystal Embolism. Circ Res (2020) 126(8):e37–52. doi: 10.1161/CIRCRESAHA.119.315625 32089086

[232] Ghanem F Vodnala D Kalavakunta JK Durga S Thormeier N Subramaniyam P. Cholesterol Crystal Embolization Following Plaque Rupture: A Systemic Disease With Unusual Features. J BioMed Res (2017) 31(2):82–94. doi: 10.7555/JBR.31.20160100 28808190 PMC5445211

[233] McDonald DM Baluk P. Significance of Blood Vessel Leakiness in Cancer. Cancer Res (2002) 62(18):5381–5. 12235011

[234] Strilic B Yang L Albarran-Juarez J Wachsmuth L Han K Muller UC. Tumour-Cell-Induced Endothelial Cell Necroptosis *via* Death Receptor 6 Promotes Metastasis. Nature (2016) 536(7615):215–8. doi: 10.1038/nature19076 27487218

[235] Melchinger H Jain K Tyagi T Hwa J. Role of Platelet Mitochondria: Life in a Nucleus-Free Zone. Front Cardiovasc Med (2019) 6:153. doi: 10.3389/fcvm.2019.00153 31737646 PMC6828734

[236] Boudreau LH Duchez AC Cloutier N Soulet D Martin N Bollinger J. Platelets Release Mitochondria Serving as Substrate for Bactericidal Group IIA-Secreted Phospholipase A2 to Promote Inflammation. Blood (2014) 124(14):2173–83. doi: 10.1182/blood-2014-05-573543 PMC4260364 25082876

[237] Melki I Allaeys I Tessandier N Levesque T Cloutier N Laroche A. Platelets Release Mitochondrial Antigens in Systemic Lupus Erythematosus. Sci Transl Med (2021) 13(581):eaav5928. doi: 10.1126/scitranslmed.aav5928. 33597264

[238] Finke D Heckmann MB Frey N Lehmann LH. Cancer-A Major Cardiac Comorbidity With Implications on Cardiovascular Metabolism. Front Physiol (2021) 12:729713. doi: 10.3389/fphys.2021.729713 34899373 PMC8662519

[239] Fearon K Strasser F Anker SD Bosaeus I Bruera E Fainsinger RL. Definition and Classification of Cancer Cachexia: An International Consensus. Lancet Oncol (2011) 12(5):489–95. doi: 10.1016/S1470-2045(10)70218-7 21296615

[240] Shimony A Zahger D Gilutz H Goldstein H Orlov G Merkin M. Cell Free DNA Detected by a Novel Method in Acute ST-Elevation Myocardial Infarction Patients. Acute Card Care (2010) 12(3):109–11. doi: 10.3109/17482941.2010.513732 20712451

[241] Khan RS Martinez MD Sy JC Pendergrass KD Che PL Brown ME. Targeting Extracellular DNA to Deliver IGF-1 to the Injured Heart. Sci Rep (2014) 4:4257. doi: 10.1038/srep04257 24604065 PMC3945489

[242] Waldenstrom A Genneback N Hellman U Ronquist G. Cardiomyocyte Microvesicles Contain DNA/RNA and Convey Biological Messages to Target Cells. PloS One (2012) 7(4):e34653. doi: 10.1371/journal.pone.0034653 22506041 PMC3323564

[243] Stroun M Anker P Maurice P Lyautey J Lederrey C Beljanski M. Neoplastic Characteristics of the DNA Found in the Plasma of Cancer Patients. Oncology (1989) 46(5):318–22. doi: 10.1159/000226740 2779946

[244] Rykova EY Laktionov PP Skvortsova TE Starikov AV Kuznetsova NP Vlassov VV. Extracellular DNA in Breast Cancer: Cell-Surface-Bound, Tumor-Derived Extracellular DNA in Blood of Patients With Breast Cancer and Nonmalignant Tumors. Ann N Y Acad Sci (2004) 1022:217–20. doi: 10.1196/annals.1318.033 15251963

[245] Skvortsova TE Vlassov VV Laktionov PP. Binding and Penetration of Methylated DNA Into Primary and Transformed Human Cells. Ann N Y Acad Sci (2008) 1137:36–40. doi: 10.1196/annals.1448.033 18837922

[246] Koyama M Kurumizaka H. Structural Diversity of the Nucleosome. J Biochem (2018) 163(2):85–95. doi: 10.1093/jb/mvx081 29161414

[247] Gardner WD Haselby JA Hoch SO. Identification of a Major Serum DNA-Binding Protein as Factor B of the Alternative Complement Pathway. J Immunol (1980) 124(6):2800–6. 6989908

[248] Gardner WD White PJ Hoch SO. Identification of a Major Human Serum DNA-Binding Protein as Beta 1H of the Alternative Pathway of Complement Activation. Biochem Biophys Res Commun (1980) 94(1):61–7. doi: 10.1016/s0006-291x(80)80187-2 6248066

[249] Kahlert C Melo SA Protopopov A Tang J Seth S Koch M. Identification of Double-Stranded Genomic DNA Spanning All Chromosomes With Mutated KRAS and P53 DNA in the Serum Exosomes of Patients With Pancreatic Cancer. J Biol Chem (2014) 289(7):3869–75. doi: 10.1074/jbc.C113.532267 PMC3924256 24398677

[250] Lazaro-Ibanez E Sanz-Garcia A Visakorpi T Escobedo-Lucea C Siljander P Ayuso-Sacido A. Different gDNA Content in the Subpopulations of Prostate Cancer Extracellular Vesicles: Apoptotic Bodies, Microvesicles, and Exosomes. Prostate (2014) 74(14):1379–90. doi: 10.1002/pros.22853 PMC4312964 25111183

[251] Vagner T Spinelli C Minciacchi VR Balaj L Zandian M Conley A. Large Extracellular Vesicles Carry Most of the Tumour DNA Circulating in Prostate Cancer Patient Plasma. J Extracell Vesicles (2018) 7(1):1505403. doi: 10.1080/20013078.2018.1505403 30108686 PMC6084494

[252] Cai J Wu G Tan X Han Y Chen C Li C. Transferred BCR/ABL DNA From K562 Extracellular Vesicles Causes Chronic Myeloid Leukemia in Immunodeficient Mice. PloS One (2014) 9(8):e105200. doi: 10.1371/journal.pone.0105200 25133686 PMC4136837

[253] Cai J Han Y Ren H Chen C He D Zhou L. Extracellular Vesicle-Mediated Transfer of Donor Genomic DNA to Recipient Cells is a Novel Mechanism for Genetic Influence Between Cells. J Mol Cell Biol (2013) 5(4):227–38. doi: 10.1093/jmcb/mjt011 PMC3733418 23580760

[254] Cai J Guan W Tan X Chen C Li L Wang N. SRY Gene Transferred by Extracellular Vesicles Accelerates Atherosclerosis by Promotion of Leucocyte Adherence to Endothelial Cells. Clin Sci (Lond) (2015) 129(3):259–69. doi: 10.1042/CS20140826 25783200

[255] Keyel PA. Dnases in Health and Disease. Dev Biol (2017) 429(1):1–11. doi: 10.1016/j.ydbio.2017.06.028 28666955 PMC11492367

[256] Cho MS Park CH Lee S Park HS. Clinicopathological Parameters for Circulating Tumor DNA Shedding in Surgically Resected non-Small Cell Lung Cancer With EGFR or KRAS Mutation. PloS One (2020) 15(3):e0230622. doi: 10.1371/journal.pone.0230622 32196518 PMC7083310

[257] Economidou-Karaoglou A Lans M Taper H Michaux JL Roberfroid M. Variations in Serum Alkaline DNase Activity: A New Means to Assess Early Detection of Relapse in Patients Treated for Acute Nonlymphoblastic Leukemia. Blood (1989) 74(8):2730–2. doi: 10.1182/blood.V74.8.2730.bloodjournal7482730 2819243

[258] Patel PS Patel BP Rawal RM Raval GN Patel MM Patel JB. Evaluation of Serum Alkaline DNase Activity in Treatment Monitoring of Head and Neck Cancer Patients. Tumour Biol (2000) 21(2):82–9. doi: 10.1159/000030113 10686537

[259] Wroblewski F Bodansky O. Presence of Desoxyribonuclease Activity in Human Serum. Proc Soc Exp Biol Med (1950) 74(2):443–5. doi: 10.3181/00379727-74-17933 15440848

[260] Tamkovich SN Cherepanova AV Kolesnikova EV Rykova EY Pyshnyi DV Vlassov VV. Circulating DNA and DNase Activity in Human Blood. Ann NY Acad Sci (2006) 1075:191–6. doi: 10.1196/annals.1368.026 17108211

[261] Cherepanova AV Tamkovich SN Bryzgunova OE Vlassov VV Laktionov PP. Deoxyribonuclease Activity and Circulating DNA Concentration in Blood Plasma of Patients With Prostate Tumors. Ann NY Acad Sci (2008) 1137:218–21. doi: 10.1196/annals.1448.016 18837950

[262] Funakoshi A Wakasugi H Ibayashi H. Clinical Investigation of Serum Deoxyribonuclease: II. Clinical Studies of Serum Deoxyribonuclease Activity in Pancreatic Disease. Gastroenterol Jpn (1979) 14(5):436–40. doi: 10.1007/BF02773731 520766

[263] Economidou-Karaoglou A Lans M Taper HS Michaux JL Roberfroid M. Variations in Serum Alkaline DNase Activity. A New Means for Therapeutic Monitoring of Malignant Lymphomas. Cancer (1988) 61(9):1838–43. doi: 10.1002/1097-0142(19880501)61:9<1838::AID-CNCR2820610920>3.0.CO;2-R 3355977

[264] Ramandanis G Agnantis N Garas J Spandidos DA. Correlation Between Serum and Tissue Deoxyribonuclease Levels in Breast Cancer Patients. Anticancer Res (1982) 2(4):213–8. 7149651

[265] Jimenez-Alcazar M Rangaswamy C Panda R Bitterling J Simsek YJ Long AT. Host DNases Prevent Vascular Occlusion by Neutrophil Extracellular Traps. Science (2017) 358(6367):1202–6. doi: 10.1126/science.aam8897 29191910

[266] Yasutomo K Horiuchi T Kagami S Tsukamoto H Hashimura C Urushihara M. Mutation of DNASE1 in People With Systemic Lupus Erythematosus. Nat Genet (2001) 28(4):313–4. doi: 10.1038/91070 11479590

[267] Grimm M Schmitt S Teriete P Biegner T Stenzl A Hennenlotter J. A Biomarker Based Detection and Characterization of Carcinomas Exploiting Two Fundamental Biophysical Mechanisms in Mammalian Cells. BMC Cancer (2013) 13:569. doi: 10.1186/1471-2407-13-569 24304513 PMC4235042

[268] Rosner K. DNase1: A New Personalized Therapy for Cancer? Expert Rev Anticancer Ther (2011) 11(7):981–4. doi: 10.1586/era.11.90 21806320

[269] Alekseeva L Mironova N. Role of Cell-Free DNA and Deoxyribonucleases in Tumor Progression. Int J Mol Sci (2021) 22(22):12246. doi: 10.3390/ijms222212246 34830126 PMC8625144

[270] Xia Y He J Zhang H Wang H Tetz G Maguire CA. AAV-Mediated Gene Transfer of DNase I in the Liver of Mice With Colorectal Cancer Reduces Liver Metastasis and Restores Local Innate and Adaptive Immune Response. Mol Oncol (2020) 14(11):2920–35. doi: 10.1002/1878-0261.12787 PMC7607180 32813937

[271] De Lamirande G. Action of Deoxyribonuclease and Ribonuclease on the Growth of Ehrlich Ascites Carcinoma in Mice. Nature (1961) 192:52–4. doi: 10.1038/192052a0 13884299

[272] Shklyaeva OA Mironova NL Malkova EM Taranov OS Ryabchikova EI Zenkova MA. Cancer-Suppressive Effect of RNase A and DNase I. Dokl Biochem Biophys (2008) 420:108–11. doi: 10.1134/S1607672908030034 18680903

[273] Patutina OA Mironova NL Ryabchikova EI Popova NA Nikolin VP Kaledin VI. Tumoricidal Activity of RNase A and DNase I. Acta Naturae (2010) 2(1):88–94. doi: 10.32607/actanaturae.10770 PMC3347544 22649632

[274] Patutina O Mironova N Ryabchikova E Popova N Nikolin V Kaledin V. Inhibition of Metastasis Development by Daily Administration of Ultralow Doses of RNase A and DNase I. Biochimie (2011) 93(4):689–96. doi: 10.1016/j.biochi.2010.12.011 21194552

[275] Alekseeva LA Sen’kova AV Zenkova MA Mironova NL. Targeting Circulating SINEs and LINEs With DNase I Provides Metastases Inhibition in Experimental Tumor Models. Mol Ther Nucleic Acids (2020) 20:50–61. doi: 10.1016/j.omtn.2020.01.035 32146418 PMC7058713

[276] Alekseeva LA Mironova NL Brenner EV Kurilshikov AM Patutina OA Zenkova MA. Alteration of the exDNA Profile in Blood Serum of LLC-Bearing Mice Under the Decrease of Tumour Invasion Potential by Bovine Pancreatic DNase I Treatment. PloS One (2017) 12(2):e0171988. doi: 10.1371/journal.pone.0171988 28222152 PMC5319761

[277] Alexeeva LA Patutina OA Sen’kova AV Zenkova MA Mironova NL. Inhibition of Invasive Properties of Murine Melanoma by Bovine Pancreatic DNase I *In Vitro* and *In Vivo* . Mol Biol (Mosk) (2017) 51(4):637–46. doi: 10.1134/S0026893317040021 28900082

[278] Alekseeva L Sen’kova A Savin I Zenkova M Mironova N. Human Recombinant DNase I (Pulmozyme((R))) Inhibits Lung Metastases in Murine Metastatic B16 Melanoma Model That Correlates With Restoration of the DNase Activity and the Decrease SINE/LINE and C-Myc Fragments in Blood Cell-Free DNA. Int J Mol Sci (2021) 22(21):12074. doi: 10.3390/ijms222112074 34769514 PMC8585023

[279] Sugihara S Yamamoto T Tanaka H Kambara T Hiraoka T Miyauchi Y. Deoxyribonuclease Treatment Prevents Blood-Borne Liver Metastasis of Cutaneously Transplanted Tumour Cells in Mice. Br J Cancer (1993) 67(1):66–70. doi: 10.1038/bjc.1993.10 8427781 PMC1968215

[280] Trejo-Becerril C Perez-Cardenas E Gutierrez-Diaz B de la Cruz-Siguenza D Taja-Chayeb L Gonzalez-Ballesteros M. Antitumor Effects of Systemic DNAse I and Proteases in an *In Vivo* Model. Integr Cancer Ther (2016) 15(4):NP35–43. doi: 10.1177/1534735416631102 27146129 PMC5739158

[281] Amundadottir LT. Pancreatic Cancer Genetics. Int J Biol Sci (2016) 12(3):314–25. doi: 10.7150/ijbs.15001 PMC4753160 26929738

[282] Feig C Gopinathan A Neesse A Chan DS Cook N Tuveson DA. The Pancreas Cancer Microenvironment. Clin Cancer Res (2012) 18(16):4266–76. doi: 10.1158/1078-0432.CCR-11-3114 PMC3442232 22896693

[283] Hisada Y Mackman N. Cancer-Associated Pathways and Biomarkers of Venous Thrombosis. Blood (2017) 130(13):1499–506. doi: 10.1182/blood-2017-03-743211 PMC5620413 28807983

[284] Padoan A Plebani M Basso D. Inflammation and Pancreatic Cancer: Focus on Metabolism, Cytokines, and Immunity. Int J Mol Sci (2019) 20(3):676. doi: 10.3390/ijms20030676 PMC6387440 30764482

[285] Wen F Shen A Choi A Gerner EW Shi J. Extracellular DNA in Pancreatic Cancer Promotes Cell Invasion and Metastasis. Cancer Res (2013) 73(14):4256–66. doi: 10.1158/0008-5472.CAN-12-3287 PMC3777608 23722544

[286] Alvarez de Haro N Van AP Robb CT Rossi AG Desbois AP. Release of Chromatin Extracellular Traps by Phagocytes of Atlantic Salmon, Salmo Salar (Linnaeus, 1758). Fish Shellfish Immunol (2021) 119:209–19. doi: 10.1016/j.fsi.2021.08.023 PMC8653909 34438058

[287] Hisada Y Grover SP Maqsood A Houston R Ay C Noubouossie DF. Neutrophils and Neutrophil Extracellular Traps Enhance Venous Thrombosis in Mice Bearing Human Pancreatic Tumors. Haematologica (2020) 105(1):218–25. doi: 10.3324/haematol.2019.217083 PMC6939515 31048354

[288] Carminita E Crescence L Brouilly N Altie A Panicot-Dubois L Dubois C. DNAse-Dependent, NET-Independent Pathway of Thrombus Formation *In Vivo* . Proc Natl Acad Sci USA (2021) 118(28):e2100561118. doi: 10.1073/pnas.2100561118 34260389 PMC8285961

[289] Darbousset R Thomas GM Mezouar S Frere C Bonier R Mackman N. Tissue Factor-Positive Neutrophils Bind to Injured Endothelial Wall and Initiate Thrombus Formation. Blood (2012) 120(10):2133–43. doi: 10.1182/blood-2012-06-437772 22837532

[290] Morrissey JH Choi SH Smith SA. Polyphosphate: An Ancient Molecule That Links Platelets, Coagulation, and Inflammation. Blood (2012) 119(25):5972–9. doi: 10.1182/blood-2012-03-306605 PMC3383012 22517894

[291] Gomez-Garcia MR Kornberg A. Formation of an Actin-Like Filament Concurrent With the Enzymatic Synthesis of Inorganic Polyphosphate. Proc Natl Acad Sci USA (2004) 101(45):15876–80. doi: 10.1073/pnas.0406923101 PMC528760 15496465

[292] Thalin C Hisada Y Lundstrom S Mackman N Wallen H. Neutrophil Extracellular Traps: Villains and Targets in Arterial, Venous, and Cancer-Associated Thrombosis. Arterioscler Thromb Vasc Biol (2019) 39(9):1724–38. doi: 10.1161/ATVBAHA.119.312463 PMC6703916 31315434

[293] Shak S Capon DJ Hellmiss R Marsters SA Baker CL. Recombinant Human DNase I Reduces the Viscosity of Cystic Fibrosis Sputum. Proc Natl Acad Sci USA (1990) 87(23):9188–92. doi: 10.1073/pnas.87.23.9188 PMC55129 2251263

[294] Fuchs HJ Borowitz DS Christiansen DH Morris EM Nash ML Ramsey BW. Effect of Aerosolized Recombinant Human DNase on Exacerbations of Respiratory Symptoms and on Pulmonary Function in Patients With Cystic Fibrosis. Pulmozyme Study Group N Engl J Med (1994) 331(10):637–42. doi: 10.1056/NEJM199409083311003 7503821

[295] Wang D Dubois RN. Eicosanoids and Cancer. Nat Rev Cancer (2010) 10(3):181–93. doi: 10.1038/nrc2809 PMC2898136 20168319

[296] Vane JR Botting RM. The Mechanism of Action of Aspirin. Thromb Res (2003) 110(5-6):255–8. doi: 10.1016/S0049-3848(03)00379-7 14592543

[297] Caudrillier A Kessenbrock K Gilliss BM Nguyen JX Marques MB Monestier M. Platelets Induce Neutrophil Extracellular Traps in Transfusion-Related Acute Lung Injury. J Clin Invest (2012) 122(7):2661–71. doi: 10.1172/JCI61303 PMC3386815 22684106

[298] Lapponi MJ Carestia A Landoni VI Rivadeneyra L Etulain J Negrotto S. Regulation of Neutrophil Extracellular Trap Formation by Anti-Inflammatory Drugs. J Pharmacol Exp Ther (2013) 345(3):430–7. doi: 10.1124/jpet.112.202879 23536315

[299] Shishikura K Horiuchi T Sakata N Trinh DA Shirakawa R Kimura T. Prostaglandin E2 Inhibits Neutrophil Extracellular Trap Formation Through Production of Cyclic AMP. Br J Pharmacol (2016) 173(2):319–31. doi: 10.1111/bph.13373 PMC5341226 26505736

[300] Domingo-Gonzalez R Martinez-Colon GJ Smith AJ Smith CK Ballinger MN Xia M. Inhibition of Neutrophil Extracellular Trap Formation After Stem Cell Transplant by Prostaglandin E2. Am J Respir Crit Care Med (2016) 193(2):186–97. doi: 10.1164/rccm.201501-0161OC PMC4731709 26417909

[301] Kimura T Takabatake Y Takahashi A Isaka Y. Chloroquine in Cancer Therapy: A Double-Edged Sword of Autophagy. Cancer Res (2013) 73(1):3–7. doi: 10.1158/0008-5472.CAN-12-2464 23288916

[302] Chargui A Cesaro A Mimouna S Fareh M Brest P Naquet P. Subversion of Autophagy in Adherent Invasive Escherichia Coli-Infected Neutrophils Induces Inflammation and Cell Death. PloS One (2012) 7(12):e51727. doi: 10.1371/journal.pone.0051727 23272151 PMC3522719

[303] Itakura A McCarty OJ. Pivotal Role for the mTOR Pathway in the Formation of Neutrophil Extracellular Traps *via* Regulation of Autophagy. Am J Physiol Cell Physiol (2013) 305(3):C348–54. doi: 10.1152/ajpcell.00108.2013 PMC3742850 23720022

[304] Park SY Shrestha S Youn YJ Kim JK Kim SY Kim HJ. Autophagy Primes Neutrophils for Neutrophil Extracellular Trap Formation During Sepsis. Am J Respir Crit Care Med (2017) 196(5):577–89. doi: 10.1164/rccm.201603-0596OC 28358992

[305] Murthy P Singhi AD Ross MA Loughran P Paragomi P Papachristou GI. Enhanced Neutrophil Extracellular Trap Formation in Acute Pancreatitis Contributes to Disease Severity and Is Reduced by Chloroquine. Front Immunol (2019) 10:28. doi: 10.3389/fimmu.2019.00028 30733719 PMC6353831

[306] Munir H Jones JO Janowitz T Hoffmann M Euler M Martins CP. Stromal-Driven and Amyloid Beta-Dependent Induction of Neutrophil Extracellular Traps Modulates Tumor Growth. Nat Commun (2021) 12(1):683. doi: 10.1038/s41467-021-20982-2 33514748 PMC7846803

[307] Kuznik A Bencina M Svajger U Jeras M Rozman B Jerala R. Mechanism of Endosomal TLR Inhibition by Antimalarial Drugs and Imidazoquinolines. J Immunol (2011) 186(8):4794–804. doi: 10.4049/jimmunol.1000702 21398612

[308] Zhou W Wang H Yang Y Chen ZS Zou C Zhang J. Chloroquine Against Malaria, Cancers and Viral Diseases. Drug Discov Today (2020) 25(11):2012–22. doi: 10.1016/j.drudis.2020.09.010 PMC7492153 32947043

[309] Cai Y Cai J Ma Q Xu Y Zou J Xu L. Chloroquine Affects Autophagy to Achieve an Anticancer Effect in EC109 Esophageal Carcinoma Cells *In Vitro* . Oncol Lett (2018) 15(1):1143–8. doi: 10.3892/ol.2017.7415 PMC5772993 29422973

[310] Thammavongsa V Missiakas DM Schneewind O. Staphylococcus Aureus Degrades Neutrophil Extracellular Traps to Promote Immune Cell Death. Science (2013) 342(6160):863–6. doi: 10.1126/science.1242255 PMC4026193 24233725

[311] Collen D Lijnen HR. Staphylokinase, a Fibrin-Specific Plasminogen Activator With Therapeutic Potential? Blood (1994) 84(3):680–6. doi: 10.1182/blood.V84.3.680.680 7519069

[312] Qi F Qi J Hu C Shen L Yu W Hu T. Conjugation of Staphylokinase With the Arabinogalactan-PEG Conjugate: Study on the Immunogenicity, *In Vitro* Bioactivity and Pharmacokinetics. Int J Biol Macromol (2019) 131:896–904. doi: 10.1016/j.ijbiomac.2019.03.046 30914374

[313] Vossenaar ER Zendman AJ van Venrooij WJ Pruijn GJ. PAD, a Growing Family of Citrullinating Enzymes: Genes, Features and Involvement in Disease. Bioessays (2003) 25(11):1106–18. doi: 10.1002/bies.10357 14579251

[314] Witalison EE Thompson PR Hofseth LJ. Protein Arginine Deiminases and Associated Citrullination: Physiological Functions and Diseases Associated With Dysregulation. Curr Drug Targets (2015) 16(7):700–10. doi: 10.2174/1389450116666150202160954 PMC4520219 25642720

[315] Wang L Song G Zhang X Feng T Pan J Chen W. PADI2-Mediated Citrullination Promotes Prostate Cancer Progression. Cancer Res (2017) 77(21):5755–68. doi: 10.1158/0008-5472.CAN-17-0150 28819028

[316] Cherrington BD Morency E Struble AM Coonrod SA Wakshlag JJ. Potential Role for Peptidylarginine Deiminase 2 (PAD2) in Citrullination of Canine Mammary Epithelial Cell Histones. PloS One (2010) 5(7):e11768. doi: 10.1371/journal.pone.0011768 20668670 PMC2909897

[317] Khan SA Edwards BS Muth A Thompson PR Cherrington BD Navratil AM. GnRH Stimulates Peptidylarginine Deiminase Catalyzed Histone Citrullination in Gonadotrope Cells. Mol Endocrinol (2016) 30(10):1081–91. doi: 10.1210/me.2016-1085 PMC5045497 27603413

[318] Wu Z Deng Q Pan B Alam HB Tian Y Bhatti UF. Inhibition of PAD2 Improves Survival in a Mouse Model of Lethal LPS-Induced Endotoxic Shock. Inflammation (2020) 43(4):1436–45. doi: 10.1007/s10753-020-01221-0 PMC7384922 32239392

[319] Li P Li M Lindberg MR Kennett MJ Xiong N Wang Y. PAD4 Is Essential for Antibacterial Innate Immunity Mediated by Neutrophil Extracellular Traps. J Exp Med (2010) 207(9):1853–62. doi: 10.1084/jem.20100239 PMC2931169 20733033

[320] Tanikawa C Espinosa M Suzuki A Masuda K Yamamoto K Tsuchiya E. Regulation of Histone Modification and Chromatin Structure by the P53-PADI4 Pathway. Nat Commun (2012) 3:676. doi: 10.1038/ncomms1676 22334079

[321] Zheng Y Zhao G Xu B Liu C Li C Zhang X. PADI4 has Genetic Susceptibility to Gastric Carcinoma and Upregulates CXCR2, KRT14 and TNF-Alpha Expression Levels. Oncotarget (2016) 7(38):62159–76. doi: 10.18632/oncotarget.11398 PMC5308718 27556695

[322] Hawez A Al-Haidari A Madhi R Rahman M Thorlacius H. MiR-155 Regulates PAD4-Dependent Formation of Neutrophil Extracellular Traps. Front Immunol (2019) 10:2462. doi: 10.3389/fimmu.2019.02462 31736940 PMC6838784

[323] Knight JS Subramanian V O’Dell AA Yalavarthi S Zhao W Smith CK. Peptidylarginine Deiminase Inhibition Disrupts NET Formation and Protects Against Kidney, Skin and Vascular Disease in Lupus-Prone MRL/lpr Mice. Ann Rheum Dis (2015) 74(12):2199–206. doi: 10.1136/annrheumdis-2014-205365 PMC4320672 25104775

[324] Wong SL Demers M Martinod K Gallant M Wang Y Goldfine AB. Diabetes Primes Neutrophils to Undergo NETosis, Which Impairs Wound Healing. Nat Med (2015) 21(7):815–9. doi: 10.1038/nm.3887 PMC4631120 26076037

[325] Lewis HD Liddle J Coote JE Atkinson SJ Barker MD Bax BD. Inhibition of PAD4 Activity is Sufficient to Disrupt Mouse and Human NET Formation. Nat Chem Biol (2015) 11(3):189–91. doi: 10.1038/nchembio.1735 PMC4397581 25622091

[326] Cedervall J Dragomir A Saupe F Zhang Y Arnlov J Larsson E. Pharmacological Targeting of Peptidylarginine Deiminase 4 Prevents Cancer-Associated Kidney Injury in Mice. Oncoimmunology (2017) 6(8):e1320009. doi: 10.1080/2162402X.2017.1320009 28919990 PMC5593702

[327] Wei L Wang X Luo M Wang H Chen H Huang C. The PAD4 Inhibitor GSK484 Enhances the Radiosensitivity of Triple-Negative Breast Cancer. Hum Exp Toxicol (2021) 40(7):1074–83. doi: 10.1177/0960327120979028 33355008

[328] McDonald B Davis RP Kim SJ Tse M Esmon CT Kolaczkowska E. Platelets and Neutrophil Extracellular Traps Collaborate to Promote Intravascular Coagulation During Sepsis in Mice. Blood (2017) 129(10):1357–67. doi: 10.1182/blood-2016-09-741298 PMC5345735 28073784

[329] Jimenez-Alcazar M Napirei M Panda R Kohler EC Kremer Hovinga JA Mannherz HG. Impaired DNase1-Mediated Degradation of Neutrophil Extracellular Traps Is Associated With Acute Thrombotic Microangiopathies. J Thromb Haemost (2015) 13(5):732–42. doi: 10.1111/jth.12796 25418346

[330] Nachat R Mechin MC Takahara H Chavanas S Charveron M Serre G. Peptidylarginine Deiminase Isoforms 1-3 are Expressed in the Epidermis and Involved in the Deimination of K1 and Filaggrin. J Invest Dermatol (2005) 124(2):384–93. doi: 10.1111/j.0022-202X.2004.23568.x 15675958

[331] Yang L Tan D Piao H. Myelin Basic Protein Citrullination in Multiple Sclerosis: A Potential Therapeutic Target for the Pathology. Neurochem Res (2016) 41(8):1845–56. doi: 10.1007/s11064-016-1920-2 27097548

[332] Lee HJ Joo M Abdolrasulnia R Young DG Choi I Ware LB. Peptidylarginine Deiminase 2 Suppresses Inhibitory {Kappa}B Kinase Activity in Lipopolysaccharide-Stimulated RAW 264. 7 Macrophages J Biol Chem (2010) 285(51):39655–62. doi: 10.1074/jbc.M110.170290 PMC3000946 20937835

[333] Krishnamurthy A Ytterberg AJ Sun M Sakuraba K Steen J Joshua V. Citrullination Controls Dendritic Cell Transdifferentiation Into Osteoclasts. J Immunol (2019) 202(11):3143–50. doi: 10.4049/jimmunol.1800534 PMC6526390 31019059

[334] Jang B Kim MJ Lee YJ Ishigami A Kim YS Choi EK. Vimentin Citrullination Probed by a Novel Monoclonal Antibody Serves as a Specific Indicator for Reactive Astrocytes in Neurodegeneration. Neuropathol Appl Neurobiol (2020) 46(7):751–69. doi: 10.1111/nan.12620 32271944

[335] Hsu PC Liao YF Lin CL Lin WH Liu GY Hung HC. Vimentin is Involved in Peptidylarginine Deiminase 2-Induced Apoptosis of Activated Jurkat Cells. Mol Cells (2014) 37(5):426–34. doi: 10.14348/molcells.2014.2359 PMC4044315 24850148

[336] Kin P U Subramanian V Nicholas AP Thompson PR Ferretti P. Modulation of Calcium-Induced Cell Death in Human Neural Stem Cells by the Novel Peptidylarginine Deiminase-AIF Pathway. Biochim Biophys Acta (o 2014) 1843(6):1162–71. doi: 10.1016/j.bbamcr.2014.02.018 PMC3996523 24607566

[337] Zhai Q Wang L Zhao P Li T. Role of Citrullination Modification Catalyzed by Peptidylarginine Deiminase 4 in Gene Transcriptional Regulation. Acta Biochim Biophys Sin (Shanghai) (2017) 49(7):567–72. doi: 10.1093/abbs/gmx042 28472221

[338] Guo Q Bedford MT Fast W. Discovery of Peptidylarginine Deiminase-4 Substrates by Protein Array: Antagonistic Citrullination and Methylation of Human Ribosomal Protein S2. Mol Biosyst (2011) 7(7):2286–95. doi: 10.1039/c1mb05089c PMC3251905 21584310

[339] Sipila K Haag S Denessiouk K Kapyla J Peters EC Denesyuk A. Citrullination of Collagen II Affects Integrin-Mediated Cell Adhesion in a Receptor-Specific Manner. FASEB J (2014) 28(8):3758–68. doi: 10.1096/fj.13-247767 24823363

[340] Deplus R Denis H Putmans P Calonne E Fourrez M Yamamoto K. Citrullination of DNMT3A by PADI4 Regulates its Stability and Controls DNA Methylation. Nucleic Acids Res (2014) 42(13):8285–96. doi: 10.1093/nar/gku522 PMC4117755 24957603

[341] Survase SA Kagliwal LD Annapure US Singhal RS. Cyclosporin A–a Review on Fermentative Production, Downstream Processing and Pharmacological Applications. Biotechnol Adv (2011) 29(4):418–35. doi: 10.1016/j.biotechadv.2011.03.004 21447377

[342] Schreiber SL Crabtree GR. The Mechanism of Action of Cyclosporin A and FK506. Immunol Today (1992) 13(4):136–42. doi: 10.1016/0167-5699(92)90111-J 1374612

[343] Gupta AK Giaglis S Hasler P Hahn S. Efficient Neutrophil Extracellular Trap Induction Requires Mobilization of Both Intracellular and Extracellular Calcium Pools and is Modulated by Cyclosporine a. PloS One (2014) 9(5):e97088. doi: 10.1371/journal.pone.0097088 24819773 PMC4018253

[344] Onishi A St Ange K Dordick JS Linhardt RJ. Heparin and Anticoagulation. Front Biosci (Landmark Ed) (2016) 21:1372–92. doi: 10.2741/4462 27100512

[345] Ma SN Mao ZX Wu Y Liang MX Wang DD Chen X. The Anti-Cancer Properties of Heparin and Its Derivatives: A Review and Prospect. Cell Adh Migr (2020) 14(1):118–28. doi: 10.1080/19336918.2020.1767489 PMC7513850 32538273

[346] Gollomp K Kim M Johnston I Hayes V Welsh J Arepally GM. Neutrophil Accumulation and NET Release Contribute to Thrombosis in HIT. JCI Insight (2018) 3(18):e99445. doi: 10.1172/jci.insight.99445 PMC6237233 30232279

[347] Lelliott PM Momota M Shibahara T Lee MSJ Smith NI Ishii KJ. Heparin Induces Neutrophil Elastase-Dependent Vital and Lytic NET Formation. Int Immunol (2020) 32(5):359–68. doi: 10.1093/intimm/dxz084 31879779

[348] Artico M Rigano R Buttari B Profumo E Ionta B Bosco S. Protective Role of Parnaparin in Reducing Systemic Inflammation and Atherosclerotic Plaque Formation in ApoE-/- Mice. Int J Mol Med (2011) 27(4):561–5. doi: 10.3892/ijmm.2011.606 21279309

[349] Zhang Y Zhao Z Guan L Mao L Li S Guan X. N-Acetyl-Heparin Attenuates Acute Lung Injury Caused by Acid Aspiration Mainly by Antagonizing Histones in Mice. PloS One (2014) 9(5):e97074. doi: 10.1371/journal.pone.0097074 24816808 PMC4016230

[350] Wildhagen KC Garcia de Frutos P Reutelingsperger CP Schrijver R Areste C Ortega-Gomez A. Nonanticoagulant Heparin Prevents Histone-Mediated Cytotoxicity *In Vitro* and Improves Survival in Sepsis. Blood (2014) 123(7):1098–101. doi: 10.1182/blood-2013-07-514984 24264231

[351] Iba T Hashiguchi N Nagaoka I Tabe Y Kadota K Sato K. Heparins Attenuated Histone-Mediated Cytotoxicity *In Vitro* and Improved the Survival in a Rat Model of Histone-Induced Organ Dysfunction. Intensive Care Med Exp (2015) 3(1):36. doi: 10.1186/s40635-015-0072-z PMC4695463 26715580

[352] Jiang J Mu S Zhang F Qiao Y Wu Y Zhang Z. [Effect of Heparin Pretreatment on the Level of Neutrophil Extracellular Traps of Serum and Lung Tissue in Septic Mice]. Zhonghua Wei Zhong Bing Ji Jiu Yi Xue (2017) 29(4):337–41. doi: 10.3760/cma.j.issn.2095-4352.2017.04.010 28420468

[353] Nishioka J Goodin S. Low-Molecular-Weight Heparin in Cancer-Associated Thrombosis: Treatment, Secondary Prevention, and Survival. J Oncol Pharm Pract (2007) 13(2):85–97. doi: 10.1177/1078155207079169 17873108

[354] Wiernsperger NF Bailey CJ. The Antihyperglycaemic Effect of Metformin: Therapeutic and Cellular Mechanisms. Drugs (1999) 58(Suppl 1):31–9; discussion 75-82. doi: 10.2165/00003495-199958001-00009 10576523

[355] Fryer LG Parbu-Patel A Carling D. The Anti-Diabetic Drugs Rosiglitazone and Metformin Stimulate AMP-Activated Protein Kinase Through Distinct Signaling Pathways. J Biol Chem (2002) 277(28):25226–32. doi: 10.1074/jbc.M202489200 11994296

[356] Menegazzo L Ciciliot S Poncina N Mazzucato M Persano M Bonora B. NETosis Is Induced by High Glucose and Associated With Type 2 Diabetes. Acta Diabetol (2015) 52(3):497–503. doi: 10.1007/s00592-014-0676-x 25387570

[357] Menegazzo L Scattolini V Cappellari R Bonora BM Albiero M Bortolozzi M. The Antidiabetic Drug Metformin Blunts NETosis *In Vitro* and Reduces Circulating NETosis Biomarkers *In Vivo* . Acta Diabetol (2018) 55(6):593–601. doi: 10.1007/s00592-018-1129-8 29546579

[358] Zhang J Bao Y Zhou X Zheng L. Polycystic Ovary Syndrome and Mitochondrial Dysfunction. Reprod Biol Endocrinol (2019) 17(1):67. doi: 10.1186/s12958-019-0509-4 31420039 PMC6698037

[359] Orio F Jr. Palomba S Cascella T Di Biase S Manguso F Tauchmanova L. The Increase of Leukocytes as a New Putative Marker of Low-Grade Chronic Inflammation and Early Cardiovascular Risk in Polycystic Ovary Syndrome. J Clin Endocrinol Metab (2005) 90(1):2–5. doi: 10.1210/jc.2004-0628 15483098

[360] Ibanez L Jaramillo AM Ferrer A de Zegher F. High Neutrophil Count in Girls and Women With Hyperinsulinaemic Hyperandrogenism: Normalization With Metformin and Flutamide Overcomes the Aggravation by Oral Contraception. Hum Reprod (2005) 20(9):2457–62. doi: 10.1093/humrep/dei072 15905296

[361] Elgendy M Ciro M Hosseini A Weiszmann J Mazzarella L Ferrari E. Combination of Hypoglycemia and Metformin Impairs Tumor Metabolic Plasticity and Growth by Modulating the PP2A-GSK3beta-MCL-1 Axis. Cancer Cell (2019) 35(5):798–815 e5. doi: 10.1016/j.ccell.2019.03.007 31031016

[362] Liu B Fan Z Edgerton SM Deng XS Alimova IN Lind SE. Metformin Induces Unique Biological and Molecular Responses in Triple Negative Breast Cancer Cells. Cell Cycle (2009) 8(13):2031–40. doi: 10.4161/cc.8.13.8814 19440038

[363] Deng XS Wang S Deng A Liu B Edgerton SM Lind SE. Metformin Targets Stat3 to Inhibit Cell Growth and Induce Apoptosis in Triple-Negative Breast Cancers. Cell Cycle (2012) 11(2):367–76. doi: 10.4161/cc.11.2.18813 22189713

[364] Cai X Hu X Tan X Cheng W Wang Q Chen X. Metformin Induced AMPK Activation, G0/G1 Phase Cell Cycle Arrest and the Inhibition of Growth of Esophageal Squamous Cell Carcinomas *In Vitro* and *In Vivo* . PloS One (2015) 10(7):e0133349. doi: 10.1371/journal.pone.0133349 26196392 PMC4510392

[365] Vazquez-Martin A Oliveras-Ferraros C Menendez JA. The Antidiabetic Drug Metformin Suppresses HER2 (erbB-2) Oncoprotein Overexpression *via* Inhibition of the mTOR Effector P70s6k1 in Human Breast Carcinoma Cells. Cell Cycle (2009) 8(1):88–96. doi: 10.4161/cc.8.1.7499 19106626

[366] Isakovic A Harhaji L Stevanovic D Markovic Z Sumarac-Dumanovic M Starcevic V. Dual Antiglioma Action of Metformin: Cell Cycle Arrest and Mitochondria-Dependent Apoptosis. Cell Mol Life Sci (2007) 64(10):1290–302. doi: 10.1007/s00018-007-7080-4 PMC11136022 17447005

[367] Ben Sahra I Laurent K Loubat A Giorgetti-Peraldi S Colosetti P Auberger P. The Antidiabetic Drug Metformin Exerts an Antitumoral Effect *In Vitro* and *In Vivo* Through a Decrease of Cyclin D1 Level. Oncogene (2008) 27(25):3576–86. doi: 10.1038/sj.onc.1211024 18212742

[368] Cantrell LA Zhou C Mendivil A Malloy KM Gehrig PA Bae-Jump VL. Metformin is a Potent Inhibitor of Endometrial Cancer Cell Proliferation–Implications for a Novel Treatment Strategy. Gynecol Oncol (2010) 116(1):92–8. doi: 10.1016/j.ygyno.2009.09.024 PMC2789879 19822355

[369] Anisimov VN Berstein LM Egormin PA Piskunova TS Popovich IG Zabezhinski MA. Effect of Metformin on Life Span and on the Development of Spontaneous Mammary Tumors in HER-2/Neu Transgenic Mice. Exp Gerontol (2005) 40(8-9):685–93. doi: 10.1016/j.exger.2005.07.007 16125352

[370] Huang X Wullschleger S Shpiro N McGuire VA Sakamoto K Woods YL. Important Role of the LKB1-AMPK Pathway in Suppressing Tumorigenesis in PTEN-Deficient Mice. Biochem J (2008) 412(2):211–21. doi: 10.1042/BJ20080557 18387000

[371] Green AS Chapuis N Maciel TT Willems L Lambert M Arnoult C. The LKB1/AMPK Signaling Pathway has Tumor Suppressor Activity in Acute Myeloid Leukemia Through the Repression of mTOR-Dependent Oncogenic mRNA Translation. Blood (2010) 116(20):4262–73. doi: 10.1182/blood-2010-02-269837 20668229

[372] Shi WY Xiao D Wang L Dong LH Yan ZX Shen ZX. Therapeutic Metformin/AMPK Activation Blocked Lymphoma Cell Growth *via* Inhibition of mTOR Pathway and Induction of Autophagy. Cell Death Dis (2012) 3:e275. doi: 10.1038/cddis.2012.13 22378068 PMC3317343

[373] Cerezo M Tichet M Abbe P Ohanna M Lehraiki A Rouaud F. Metformin Blocks Melanoma Invasion and Metastasis Development in AMPK/p53-Dependent Manner. Mol Cancer Ther (2013) 12(8):1605–15. doi: 10.1158/1535-7163.MCT-12-1226-T 23741061

[374] Wang Y Xu W Yan Z Zhao W Mi J Li J. Metformin Induces Autophagy and G0/G1 Phase Cell Cycle Arrest in Myeloma by Targeting the AMPK/mTORC1 and Mtorc2 Pathways. J Exp Clin Cancer Res (2018) 37(1):63. doi: 10.1186/s13046-018-0731-5 29554968 PMC5859411

[375] Shi B Hu X He H Fang W. Metformin Suppresses Breast Cancer Growth *via* Inhibition of Cyclooxygenase-2. Oncol Lett (2021) 22(2):615. doi: 10.3892/ol.2021.12876 34257723 PMC8243079

[376] Tomimoto A Endo H Sugiyama M Fujisawa T Hosono K Takahashi H. Metformin Suppresses Intestinal Polyp Growth in ApcMin/+ Mice. Cancer Sci (2008) 99(11):2136–41. doi: 10.1111/j.1349-7006.2008.00933.x PMC11159964 18803638

[377] Martin FA Murphy RP Cummins PM. Thrombomodulin and the Vascular Endothelium: Insights Into Functional, Regulatory, and Therapeutic Aspects. Am J Physiol Heart Circ Physiol (2013) 304(12):H1585–97. doi: 10.1152/ajpheart.00096.2013 PMC7212260 23604713

[378] Delvaeye M Noris M De Vriese A Esmon CT Esmon NL Ferrell G. Thrombomodulin Mutations in Atypical Hemolytic-Uremic Syndrome. N Engl J Med (2009) 361(4):345–57. doi: 10.1056/NEJMoa0810739 PMC3530919 19625716

[379] Yoshihara M Uno K Tano S Mayama M Ukai M Kondo S. The Efficacy of Recombinant Human Soluble Thrombomodulin for Obstetric Disseminated Intravascular Coagulation: A Retrospective Study. Crit Care (2015) 19:369. doi: 10.1186/s13054-015-1086-3 26481315 PMC4617479

[380] Watanabe-Kusunoki K Nakazawa D Ishizu A Atsumi T. Thrombomodulin as a Physiological Modulator of Intravascular Injury. Front Immunol (2020) 11:575890. doi: 10.3389/fimmu.2020.575890 33042158 PMC7525002

[381] Shimomura Y Suga M Kuriyama N Nakamura T Sakai T Kato Y. Recombinant Human Thrombomodulin Inhibits Neutrophil Extracellular Trap Formation *In Vitro* . J Intensive Care (2016) 4:48. doi: 10.1186/s40560-016-0177-9 27453785 PMC4957921

[382] Helms J Clere-Jehl R Bianchini E Le Borgne P Burban M Zobairi F. Thrombomodulin Favors Leukocyte Microvesicle Fibrinolytic Activity, Reduces NETosis and Prevents Septic Shock-Induced Coagulopathy in Rats. Ann Intensive Care (2017) 7(1):118. doi: 10.1186/s13613-017-0340-z 29222696 PMC5722785

[383] Dahlback B Villoutreix BO. Regulation of Blood Coagulation by the Protein C Anticoagulant Pathway: Novel Insights Into Structure-Function Relationships and Molecular Recognition. Arterioscler Thromb Vasc Biol (2005) 25(7):1311–20. doi: 10.1161/01.ATV.0000168421.13467.82 15860736

[384] Kalil AC LaRosa SP. Effectiveness and Safety of Drotrecogin Alfa (Activated) for Severe Sepsis: A Meta-Analysis and Metaregression. Lancet Infect Dis (2012) 12(9):678–86. doi: 10.1016/S1473-3099(12)70157-3 22809883

[385] Feistritzer C Riewald M. Endothelial Barrier Protection by Activated Protein C Through PAR1-Dependent Sphingosine 1-Phosphate Receptor-1 Crossactivation. Blood (2005) 105(8):3178–84. doi: 10.1182/blood-2004-10-3985 15626732

[386] Van Sluis GL Niers TM Esmon CT Tigchelaar W Richel DJ Buller HR. Endogenous Activated Protein C Limits Cancer Cell Extravasation Through Sphingosine-1-Phosphate Receptor 1-Mediated Vascular Endothelial Barrier Enhancement. Blood (2009) 114(9):1968–73. doi: 10.1182/blood-2009-04-217679 PMC2738579 19571314

[387] Healy LD Puy C Fernandez JA Mitrugno A Keshari RS Taku NA. Activated Protein C Inhibits Neutrophil Extracellular Trap Formation *In Vitro* and Activation *In Vivo* . J Biol Chem (2017) 292(21):8616–29. doi: 10.1074/jbc.M116.768309 PMC5448091 28408624

[388] Uchiba M Okajima K Oike Y Ito Y Fukudome K Isobe H. Activated Protein C Induces Endothelial Cell Proliferation by Mitogen-Activated Protein Kinase Activation *In Vitro* and Angiogenesis *In Vivo* . Circ Res (2004) 95(1):34–41. doi: 10.1161/01.RES.0000133680.87668.FA 15166095

[389] Beaulieu LM Church FC. Activated Protein C Promotes Breast Cancer Cell Migration Through Interactions With EPCR and PAR-1. Exp Cell Res (2007) 313(4):677–87. doi: 10.1016/j.yexcr.2006.11.019 PMC4055951 17254565

[390] Holland PC Clark MG Bloxham DP Lardy HA. Mechanism of Action of the Hypoglycemic Agent Diphenyleneiodonium. J Biol Chem (1973) 248(17):6050–6. doi: 10.1016/S0021-9258(19)43506-0 4726296

[391] Pandey M Singh AK Thakare R Talwar S Karaulia P Dasgupta A. Diphenyleneiodonium Chloride (DPIC) Displays Broad-Spectrum Bactericidal Activity. Sci Rep (2017) 7(1):11521. doi: 10.1038/s41598-017-11575-5 28912539 PMC5599662

[392] Stuehr DJ Fasehun OA Kwon NS Gross SS Gonzalez JA Levi R. Inhibition of Macrophage and Endothelial Cell Nitric Oxide Synthase by Diphenyleneiodonium and its Analogs. FASEB J (1991) 5(1):98–103. doi: 10.1096/fasebj.5.1.1703974 1703974

[393] Sanders SA Eisenthal R Harrison R. NADH Oxidase Activity of Human Xanthine Oxidoreductase–Generation of Superoxide Anion. Eur J Biochem (1997) 245(3):541–8. doi: 10.1111/j.1432-1033.1997.00541.x 9182988

[394] Tew DG. Inhibition of Cytochrome P450 Reductase by the Diphenyliodonium Cation. Kinetic Analysis and Covalent Modifications. Biochemistry (1993) 32(38):10209–15. doi: 10.1021/bi00089a042 8399148

[395] Ozsvari B Bonuccelli G Sanchez-Alvarez R Foster R Sotgia F Lisanti MP. Targeting Flavin-Containing Enzymes Eliminates Cancer Stem Cells (CSCs), by Inhibiting Mitochondrial Respiration: Vitamin B2 (Riboflavin) in Cancer Therapy. Aging (Albany NY) (2017) 9(12):2610–28. doi: 10.18632/aging.101351 PMC5764395 29253841

[396] Piszczatowska K Przybylska D Sikora E Mosieniak G. Inhibition of NADPH Oxidases Activity by Diphenyleneiodonium Chloride as a Mechanism of Senescence Induction in Human Cancer Cells. Antioxid (Basel) (2020) 9(12):1248. doi: 10.3390/antiox9121248 PMC7764543 33302580

[397] Sabbione F Keitelman IA Iula L Ferrero M Giordano MN Baldi P. Neutrophil Extracellular Traps Stimulate Proinflammatory Responses in Human Airway Epithelial Cells. J Innate Immun (2017) 9(4):387–402. doi: 10.1159/000460293 28467984 PMC6738901

[398] Zhang H Qiu SL Tang QY Zhou X Zhang JQ He ZY. Erythromycin Suppresses Neutrophil Extracellular Traps in Smoking-Related Chronic Pulmonary Inflammation. Cell Death Dis (2019) 10(9):678. doi: 10.1038/s41419-019-1909-2 31515489 PMC6742640

[399] Mandke P Vasquez KM. Interactions of High Mobility Group Box Protein 1 (HMGB1) With Nucleic Acids: Implications in DNA Repair and Immune Responses. DNA Repair (Amst) (2019) 83:102701. doi: 10.1016/j.dnarep.2019.102701 31563843 PMC6906087

[400] Stark K Philippi V Stockhausen S Busse J Antonelli A Miller M. Disulfide HMGB1 Derived From Platelets Coordinates Venous Thrombosis in Mice. Blood (2016) 128(20):2435–49. doi: 10.1182/blood-2016-04-710632 PMC5147023 27574188

[401] Yang H Antoine DJ Andersson U Tracey KJ. The Many Faces of HMGB1: Molecular Structure-Functional Activity in Inflammation, Apoptosis, and Chemotaxis. J Leukoc Biol (2013) 93(6):865–73. doi: 10.1189/jlb.1212662 PMC4051189 23446148

[402] Su Z Zhang P Yu Y Lu H Liu Y Ni P. HMGB1 Facilitated Macrophage Reprogramming Towards a Proinflammatory M1-Like Phenotype in Experimental Autoimmune Myocarditis Development. Sci Rep (2016) 6:21884. doi: 10.1038/srep21884 26899795 PMC4761996

[403] Tadie JM Bae HB Jiang S Park DW Bell CP Yang H. HMGB1 Promotes Neutrophil Extracellular Trap Formation Through Interactions With Toll-Like Receptor 4. Am J Physiol Lung Cell Mol Physiol (2013) 304(5):L342–9. doi: 10.1152/ajplung.00151.2012 PMC3602738 23316068

[404] Horiuchi T Sakata N Narumi Y Kimura T Hayashi T Nagano K. Metformin Directly Binds the Alarmin HMGB1 and Inhibits its Proinflammatory Activity. J Biol Chem (2017) 292(20):8436–46. doi: 10.1074/jbc.M116.769380 PMC5437248 28373282

[405] Tsoyi K Jang HJ Nizamutdinova IT Kim YM Lee YS Kim HJ. Metformin Inhibits HMGB1 Release in LPS-Treated RAW 264.7 Cells and Increases Survival Rate of Endotoxaemic Mice. Br J Pharmacol (2011) 162(7):1498–508. doi: 10.1111/j.1476-5381.2010.01126.x PMC3057288 21091653

[406] Vogel S Bodenstein R Chen Q Feil S Feil R Rheinlaender J. Platelet-Derived HMGB1 Is a Critical Mediator of Thrombosis. J Clin Invest (2015) 125(12):4638–54. doi: 10.1172/JCI81660 PMC4665785 26551681

[407] Yu LX Yan L Yang W Wu FQ Ling Y Chen SZ. Platelets Promote Tumour Metastasis *via* Interaction Between TLR4 and Tumour Cell-Released High-Mobility Group Box1 Protein. Nat Commun (2014) 5:5256. doi: 10.1038/ncomms6256 25348021

[408] Hoste E Maueroder C van Hove L Catrysse L Vikkula HK Sze M. Epithelial HMGB1 Delays Skin Wound Healing and Drives Tumor Initiation by Priming Neutrophils for NET Formation. Cell Rep (2019) 29(9):2689–701.e4. doi: 10.1016/j.celrep.2019.10.104 31775038

[409] Angiolillo DJ Rollini F Storey RF Bhatt DL James S Schneider DJ. International Expert Consensus on Switching Platelet P2Y12 Receptor-Inhibiting Therapies. Circulation (2017) 136(20):1955–75. doi: 10.1161/CIRCULATIONAHA.117.031164 29084738

[410] Gachet C. ADP Receptors of Platelets and Their Inhibition. Thromb Haemost (2001) 86(1):222–32. doi: 10.1055/s-0037-1616220 11487010

[411] Elaskalani O Abdol Razak NB Metharom P. Neutrophil Extracellular Traps Induce Aggregation of Washed Human Platelets Independently of Extracellular DNA and Histones. Cell Commun Signal (2018) 16(1):24. doi: 10.1186/s12964-018-0235-0 29843771 PMC5975482

[412] Kaneider NC Egger P Dunzendorfer S Wiedermann CJ. Rho-GTPase-Dependent Platelet-Neutrophil Interaction Affected by HMG-CoA Reductase Inhibition With Altered Adenosine Nucleotide Release and Function. Arterioscler Thromb Vasc Biol (2002) 22(6):1029–35. doi: 10.1161/01.ATV.0000018306.68268.86 12067916

[413] Chrysanthopoulou A Kambas K Stakos D Mitroulis I Mitsios A Vidali V. Interferon Lambda1/IL-29 and Inorganic Polyphosphate are Novel Regulators of Neutrophil-Driven Thromboinflammation. J Pathol (2017) 243(1):111–22. doi: 10.1002/path.4935 28678391

[414] Stakos DA Kambas K Konstantinidis T Mitroulis I Apostolidou E Arelaki S. Expression of Functional Tissue Factor by Neutrophil Extracellular Traps in Culprit Artery of Acute Myocardial Infarction. Eur Heart J (2015) 36(22):1405–14. doi: 10.1093/eurheartj/ehv007 PMC4458286 25660055

[415] Mitsios A Chrysanthopoulou A Arampatzioglou A Angelidou I Vidali V Ritis K. Ticagrelor Exerts Immune-Modulatory Effect by Attenuating Neutrophil Extracellular Traps. Int J Mol Sci (2020) 21(10):3625. doi: 10.3390/ijms21103625 PMC7279443 32455533

[416] Jin L Kim HS Shi J. Neutrophil in the Pancreatic Tumor Microenvironment. Biomolecules (2021) 11(8):1170. doi: 10.3390/biom11081170 34439836 PMC8394314

[417] Palacios-Acedo AL Mezouar S Mege D Crescence L Dubois C Panicot-Dubois L. P2RY12-Inhibitors Reduce Cancer-Associated Thrombosis and Tumor Growth in Pancreatic Cancers. Front Oncol (2021) 11:704945. doi: 10.3389/fonc.2021.704945 34589424 PMC8475274

[418] Wright C Moore RD. Disulfiram Treatment of Alcoholism. Am J Med (1990) 88(6):647–55. doi: 10.1016/0002-9343(90)90534-K 2189310

[419] Hu JJ Liu X Xia S Zhang Z Zhang Y Zhao J. FDA-Approved Disulfiram Inhibits Pyroptosis by Blocking Gasdermin D Pore Formation. Nat Immunol (2020) 21(7):736–45. doi: 10.1038/s41590-020-0669-6 PMC7316630 32367036

[420] Silva CMS Wanderley CWS Veras FP Sonego F Nascimento DC Goncalves AV. Gasdermin D Inhibition Prevents Multiple Organ Dysfunction During Sepsis by Blocking NET Formation. Blood (2021) 138(25):2702–13. doi: 10.1182/blood.2021011525 PMC8703366 34407544

[421] Sborgi L Ruhl S Mulvihill E Pipercevic J Heilig R Stahlberg H. GSDMD Membrane Pore Formation Constitutes the Mechanism of Pyroptotic Cell Death. EMBO J (2016) 35(16):1766–78. doi: 10.15252/embj.201694696 PMC5010048 27418190

[422] Hagar JA Powell DA Aachoui Y Ernst RK Miao EA. Cytoplasmic LPS Activates Caspase-11: Implications in TLR4-Independent Endotoxic Shock. Science (2013) 341(6151):1250–3. doi: 10.1126/science.1240988 PMC3931427 24031018

[423] Kambara H Liu F Zhang X Liu P Bajrami B Teng Y. Gasdermin D Exerts Anti-Inflammatory Effects by Promoting Neutrophil Death. Cell Rep (2018) 22(11):2924–36. doi: 10.1016/j.celrep.2018.02.067 PMC5878047 29539421

[424] Maizels RM Denham DA. Diethylcarbamazine (DEC): Immunopharmacological Interactions of an Anti-Filarial Drug. Parasitology (1992) 105(Suppl):S49–60. doi: 10.1017/S0031182000075351 1308929

[425] Medina-De la Garza CE Guerrero-Ramirez G Garcia-Hernandez M Castro-Corona MA Torres-Lopez E Brattig NW. Immunomodulatory Activity of Diethylcarbamazine on Humoral, Cellular Cytokine Response and Respiratory Burst in BALB/c Mice. Immunopharmacol Immunotoxicol (2012) 34(3):477–83. doi: 10.3109/08923973.2011.630008 22564175

[426] Garcia-Hernandez M Castro-Corona MA Segoviano-Ramirez JC Brattig NW Medina-De la Garza CE. Immunomodulatory Effect of Diethylcarbamazine in Mice Infected With Nocardia Brasiliensis. Int Immunopharmacol (2014) 23(1):113–20. doi: 10.1016/j.intimp.2014.08.004 25150175

[427] Segoviano Ramirez JC de la Rosa Tamez S Garcia Juarez J de los Angeles Castro Corona M Medina de la Garza CE. Analysis of the Immunomodulatory Effect of Diethylcarbamazine (DEC) on New Mechanisms of Antiinfectious Response in Human Polymorphonuclear Cells, (Pilot Study). Histol Histopathol (2017) 32:50. doi: 10.1155/2020/4827641

[428] Segoviano-Ramirez JC Lopez-Altamirano DF Garcia-Juarez J Aguirre-Garza JES Cardenas-Estrada E Ancer-Rodriguez J. The Diethylcarbamazine Delays and Decreases the NETosis of Polymorphonuclear Cells of Humans With DM Type 2. J Diabetes Res (2020) 2020:4827641. doi: 10.1155/2020/4827641 32190698 PMC7072105

[429] da Silva BS Rodrigues GB Rocha SW Ribeiro EL Gomes FO AK ES. Inhibition of NF-kappaB Activation by Diethylcarbamazine Prevents Alcohol-Induced Liver Injury in C57BL/6 Mice. Tissue Cell (2014) 46(5):363–71. doi: 10.1016/j.tice.2014.06.008 25059110

[430] Monari C Bevilacqua S Piccioni M Pericolini E Perito S Calvitti M. A Microbial Polysaccharide Reduces the Severity of Rheumatoid Arthritis by Influencing Th17 Differentiation and Proinflammatory Cytokines Production. J Immunol (2009) 183(1):191–200. doi: 10.4049/jimmunol.0804144 19542430

[431] Rocha JD Nascimento MT Decote-Ricardo D Corte-Real S Morrot A Heise N. Capsular Polysaccharides From Cryptococcus Neoformans Modulate Production of Neutrophil Extracellular Traps (NETs) by Human Neutrophils. Sci Rep (2015) 5:8008. doi: 10.1038/srep08008 25620354 PMC4306120

[432] Szekanecz Z Soos L Szabo Z Fekete A Kapitany A Vegvari A. Anti-Citrullinated Protein Antibodies in Rheumatoid Arthritis: As Good as it Gets? Clin Rev Allergy Immunol (2008) 34(1):26–31. doi: 10.1007/s12016-007-8022-5 18270854

[433] Katayama H Kobayashi M Irajizad E Sevillarno A Patel N Mao X. Protein Citrullination as a Source of Cancer Neoantigens. J Immunother Cancer (2021) 9(6):e002549. doi: 10.1136/jitc-2021-002549 34112737 PMC8194337

[434] Chirivi RGS van Rosmalen JWG van der Linden M Euler M Schmets G Bogatkevich G. Therapeutic ACPA Inhibits NET Formation: A Potential Therapy for Neutrophil-Mediated Inflammatory Diseases. Cell Mol Immunol (2021) 18(6):1528–44. doi: 10.1038/s41423-020-0381-3 PMC8166830 32203195

